# Supplementary material for: Factors that influence vaccination decision-making among pregnant women: A systematic review and meta-analysis
Source: PLoS One. 2020 Jul 9;15(7):e0234827. doi: 10.1371/journal.pone.0234827 (PMC7347125; doi:10.1371/journal.pone.0234827)
Supplement: S2 File — (DOCX) [file pone.0234827.s003.docx]

**Supplementary Material**

Supplement to Kilich E, Dada S, Francis M, Tazare J, Chico RM, Paterson P, Larson H. Factors that influence vaccination decision-making among pregnant women: a systematic review and meta-analysis.

**Table of Contents**

[**Appendix 1. Population and Outcome of Interest** 2](#_Toc32761749)

[**Appendix 2. Search strategy** 3](#_Toc32761750)

[**Appendix 3. Inclusion/Exclusion criteria** 4](#_Toc32761751)

[**Appendix 4. Database search queries** 5](#_Toc32761752)

[**Appendix 5. Reasons for exclusion** 11](#_Toc32761753)

[**Appendix 6. PRISMA Checklist^91^** 16](#_Toc32761754)

[**Appendix 7. Expanded methodology** 18](#_Toc32761755)

[**Appendix 8. Joanna Briggs Institute (JBI) Quality Assessment** 21](#_Toc32761756)

[**Appendix 9. Distribution of included studies by country and vaccine** 33](#_Toc32761757)

[**Appendix 10. Broad theme definitions.** 38](#_Toc32761758)

[**Appendix 11. Coding process.** 40](#_Toc32761759)

[**Appendix 12. Meta-analysis sub-category definitions.** 41](#_Toc32761760)

[**Appendix 13. Pooled questions and responses for meta-analysis** 42](#_Toc32761761)

[**Appendix 14. Reasons for exclusion from meta-analysis** 44](#_Toc32761762)

[**Appendix 15. Authors contacted for further data where data was insufficient** 51](#_Toc32761763)

[**Appendix 16. Most common facilitator/barrier cited in quantitative papers excluded from meta-analysis** 52](#_Toc32761764)

[**Appendix 17. Funnel plot of HCP recommendation for seasonal influenza vaccination** 54](#_Toc32761765)

[**Appendix 18. Individual study forest plot for Pandemic Influenza and primary outcome of vaccination status** 55](#_Toc32761766)

[**Appendix 19. Individual study forest plot for Pertussis – primary outcome** 70](#_Toc32761767)

[**Appendix 20. Individual study forest plot for Seasonal Influenza – primary outcome** 71](#_Toc32761768)

[**Appendix 22. Table – Summary ORs from quality sensitivity analysis: meta-analyses investigating association between beliefs/experiences and vaccination in studies with JBI score > 10** 90](#_Toc32761769)

[**Appendix 23. Table – Summary ORs from secondary analysis: meta-analyses investigating association between beliefs/experiences and intention to vaccinate** 91](#_Toc32761770)

[**Appendix 24. Table – Summary ORs from secondary analysis: meta-analyses investigating association between beliefs/experiences and intention to vaccinate in studies with JBI score > 10** 92](#_Toc32761771)

[**Appendix 25: Table –OR from one study meta-analyses investigating association between beliefs/experiences and vaccination in studies** 93](#_Toc32761772)

[**Supplementary Material References** 94](#_Toc32761773)

# **Appendix 1. Population and Outcome of Interest**

Our population of interest was pregnant or recently pregnant women (within 2 years of delivery). We were interested in the beliefs and experiences of this population regarding vaccinations received during pregnancy. Two years was chosen The primary outcome of interest was vaccination status – some studies relied on self-reported vaccination status while other studies used medical records to verify status. Our secondary outcome was intention to vaccinate as not all studies reported actual status.

# **Appendix 2. Search strategy**

The search strategy used in this study was developed in a prior literature review published in 2015.^1^ The search strategy included an extensive list of keywords ([Table 1](https://www.sciencedirect.com/science/article/pii/S0264410X15011731?via%3Dihub#tbl0005)) and related MeSH/subject headings in an effort to capture the many dimensions and expressions of vaccine confidence, trust and hesitancy. Search by Title, Abstract and MESH, subject headings.

**Table 1. Keywords for search strategy for systematic review on vaccine hesitancy.**

| vaccin* OR immunis* OR immuniz*  AND  anxiety OR doubt* OR trust* OR intent* OR dilemma* OR attitude* OR distrust OR mistrust OR controvers* OR objector* OR awareness OR dropout* OR Perception* OR misconception* OR uptake OR behavi*r OR exemption* OR refus* OR misinformation OR barrier* OR belief* OR fear* OR reject* OR oppos* OR choice* OR criticis* OR hesitanc* OR rumo*r OR delay OR mandatory OR accept* OR concern* OR compulsory OR knowledge OR confiden* OR decision making OR anti-vaccin* OR “parent*confiden*”  AND  maternal OR antenatal OR prenatal OR pregnan* |
| --- |

No additional limits

**Search Databases:** Medline, Embase Classic & Embase, PsychInfo, CINAHL Plus, Web of Science, IBSS, LILACS, AfricaWideInfo, IMEMR, and Global Health.

# **Appendix 3. Inclusion/Exclusion criteria**

Inclusion criteria

Articles that include research on the following:

- Vaccine hesitancy, trust/distrust, perceptions, concerns, confidence, attitudes, beliefs about vaccines and vaccination programmes in pregnancy by

Location: any

Publication years: any

Vaccine: vaccines provided during pregnancy (Influenza, Tdap, DTaP/IPV/ tetanus vaccine)

Concerns: all concerns

Populations: pregnant women, women who have recently given birth (within 2 years)

Exclusion criteria

- Exclusively about experimental vaccines during pregnancy
- Non-peer reviewed articles such as editorials, letters, comment/opinion, conference articles pilot studies.
- Safety and vaccine development research
  - Immunogenicity and serological studies
  - Pre-clinical trial(s)
  - Efficacy trial(s)
- Cost-benefit analysis or cost-effectiveness studies.
- Views of the public or healthcare workers
- Animal vaccination
- Randomised control trials

# **Appendix 4. Database search queries**

| **21/11/2018-22/11/2018** | | **Databases search queries entered** | | |
| --- | --- | --- | --- | --- |
|  |  |  |  | |
| **Ovid MEDLINE(R) 1947 to 2018 November 21** | | | | |
|  |  |  |  | |
|  | 1 | (vaccin* or immunis* or immuniz*).ti,ab. | | 327,667 |
|  | 2 | (anxiety or doubt* or trust* or intent* or dilemma* or attitude* or distrust or mistrust or controvers* or objector* or awareness or dropout* or Perception* or misconception* or uptake or behavi#r or exemption* or refus* or misinformation or barrier* or belief* or fear* or reject* or oppos* or choice* or criticis* or hesitanc* or rumo?r or delay or mandatory or accept* or concern* or compulsory or knowledge or confiden* or decision making or anti-vaccin* or parent*-confiden*).ti,ab. | | 3,626,238 |
|  | 3 | (maternal or antenatal* or prenatal* or pregnan*).ti,ab. | | 585,707 |
|  | 4 | Vaccines/ | | 20,142 |
|  | 5 | immunizations/ | | 48,948 |
|  | 6 | vaccinations/ | | 75,407 |
|  | 7 | health attitudes/ | | 80,768 |
|  | 8 | health behavior/ | | 45,420 |
|  | 9 | Health Knowledge/ | | 99,248 |
|  | 10 | Decision making/ | | 85,764 |
|  | 11 | Treatment Refusal/ | | 11,469 |
|  | 12 | anxiety/ | | 72,016 |
|  | 13 | trust/ | | 8,325 |
|  | 14 | Attitude/ | | 44,851 |
|  | 15 | awareness/ | | 18,454 |
|  | 16 | perception/ | | 30,696 |
|  | 17 | Vaccination Refusal/ | | 133 |
|  | 18 | fear/ | | 28,775 |
|  | 19 | rejection/ | | 1,519 |
|  | 20 | Choice Behavior/ | | 29,889 |
|  | 21 | patient acceptance of health care/ | | 40,519 |
|  | 22 | Maternal Behavior/ | | 10,770 |
|  | 23 | Prenatal Care/ | | 24,875 |
|  | 24 | 1 or 4 or 5 or 6 | | 359,215 |
|  | 25 | 2 or 7 or 8 or 9 or 10 or 11 or 12 or 13 or 14 or 15 or 16 or 17 or 18 or 19 or 20 or 21 | | 3,810,965 |
|  | 26 | 3 or 22 or 23 | | 595,287 |
|  | 27 | 24 and 25 and 26 | | 3,729 |
|  |  |  |  | |
| **Embase Classic+Embase 1947 to 2018 November 19** | | | | |
|  | 1 | (vaccin* or immunis* or immuniz*).ti,ab. | | 452,339 |
|  | 2 | (anxiety or doubt* or trust* or intent* or dilemma* or attitude* or distrust or mistrust or controvers* or objector* or awareness or dropout* or Perception* or misconception* or uptake or behavi#r or exemption* or refus* or misinformation or barrier* or belief* or fear* or reject* or oppos* or choice* or criticis* or hesitanc* or rumo?r or delay or mandatory or accept* or concern* or compulsory or knowledge or confiden* or decision making or anti-vaccin* or parent*-confiden*).ti,ab. | | 5,598,059 |
|  | 3 | (maternal or antenatal* or prenatal* or pregnan*).ti,ab. | | 872,769 |
|  | 4 | Vaccines/ | | 62,392 |
|  | 5 | immunization/ | | 104,949 |
|  | 6 | vaccination/ | | 144,460 |
|  | 7 | attitude to health/ | | 104,462 |
|  | 8 | health behavior/ | | 60,056 |
|  | 9 | decision making/ | | 206,110 |
|  | 10 | treatment refusal/ | | 17,059 |
|  | 11 | anxiety/ | | 188,661 |
|  | 12 | trust/ | | 15,896 |
|  | 13 | attitude/ | | 60,598 |
|  | 14 | awareness/ | | 64,747 |
|  | 15 | perception/ | | 99,789 |
|  | 16 | vaccination refusal/ | | 196 |
|  | 17 | fear/ | | 59,630 |
|  | 18 | maternal behavior/ | | 12,729 |
|  | 19 | prenatal care/ | | 36,874 |
|  | 20 | 1 or 4 or 5 or 6 | | 503,289 |
|  | 21 | 2 or 7 or 8 or 9 or 10 or 11 or 12 or 13 or 14 or 15 or 16 or 17 | | 5,857,644 |
|  | 22 | 3 or 18 or 19 | | 884,369 |
|  | 23 | 20 and 21 and 22 | | 5,690 |
|  |  |  | |  |
| **# ▲PsycINFO 1806 to November Week 3 2018** | | | | |
|  | 1 | (vaccin* or immunis* or immuniz*).ti,ab. | | 8,094 |
|  | 2 | (anxiety or doubt* or trust* or intent* or dilemma* or attitude* or distrust or mistrust or controvers* or objector* or awareness or dropout* or Perception* or misconception* or uptake or behavi#r or exemption* or refus* or misinformation or barrier* or belief* or fear* or reject* or oppos* or choice* or criticis* or hesitanc* or rumo?r or delay or mandatory or accept* or concern* or compulsory or knowledge or confiden* or decision making or anti-vaccin* or parent*-confiden*).ti,ab. | | 1,983,114 |
|  | 3 | (maternal or antenatal* or prenatal* or pregnan*).ti,ab. | | 92,445 |
|  | 4 | IMMUNIZATION/ | | 4,227 |
|  | 5 | attitudes/ | | 25,344 |
|  | 6 | Health Behavior/ | | 25,692 |
|  | 7 | decision making/ | | 68,745 |
|  | 8 | treatment refusal/ | | 729 |
|  | 9 | ANXIETY/ | | 56,836 |
|  | 10 | "TRUST (SOCIAL BEHAVIOR)"/ | | 9,486 |
|  | 11 | ATTITUDES/ | | 25,344 |
|  | 12 | HEALTH ATTITUDES/ | | 9,790 |
|  | 13 | AWARENESS/ | | 12,914 |
|  | 14 | PERCEPTION/ | | 22,046 |
|  | 15 | Parental Attitudes/ | | 16,737 |
|  | 16 | Prenatal Care/ | | 1,698 |
|  | 17 | 1 or 4 | | 8,246 |
|  | 18 | 2 or 5 or 6 or 7 or 8 or 9 or 10 or 11 or 12 or 13 or 14 or 15 | | 2,021,487 |
|  | 19 | 3 or 16 | | 92,536 |
|  | 20 | 17 and 18 and 19 | | 342 |
|  |  |  | |  |
| **CINAHL Plus** | | | | |
|  | S19 | S16 AND S17 AND S18 | | 1,500 |
|  | S18 | S3 OR S6 OR S7 OR S8 | | 231,057 |
|  | S17 | S2 OR S9 OR S10 OR S11 OR S12 OR S13 OR S14 OR S15 | | 1,438,794 |
|  | S16 | S1 OR S4 OR S5 | | 53,906 |
|  | S15 | (MM "Decision Making") | | 18,965 |
|  | S14 | (MM "Knowledge") OR (MM "Health Knowledge") | | 16,227 |
|  | S13 | (MM "Trust") OR (MM "Antitrust") | | 3,214 |
|  | S12 | (MM "Fear") | | 4,455 |
|  | S11 | (MM "Anxiety") | | 15,536 |
|  | S10 | (MM "Confidence") | | 1,746 |
|  | S9 | (MM "Attitude") | | 8,356 |
|  | S8 | (MM "Pregnancy") | | 3,644 |
|  | S7 | (MM "Prenatal Care") | | 7,833 |
|  | S6 | (MM "Maternal Behavior") OR (MM "Maternal Attitudes") | | 5,551 |
|  | S5 | (MM "Vaccines") | | 4,087 |
|  | S4 | (MM "Immunization") | | 12,105 |
|  | S3 | (TI maternal or antenatal* or prenatal* or pregnan*) OR (AB maternal or antenatal* or prenatal* or pregnan*) | | 229,077 |
|  | S2 | (TI anxiety or doubt* or trust* or intent* or dilemma* or attitude* or distrust or mistrust or controvers* or objector* or awareness or dropout* or Perception* or misconception* or uptake or behavi#r or exemption* or refus* or misinformation or barrier* or belief* or fear* or reject* or oppos* or choice* or criticis* or hesitanc* or rumo?r or delay or mandatory or accept* or concern* or compulsory or knowledge or confiden* or decision making or anti-vaccin* or parent*-confiden*) OR (AB anxiety o ... | | 1,436,307 |
|  | S1 | (TI vaccin* OR immunis* OR immuniz*) OR (AB vaccin* OR immunis* OR immuniz*) | | 53,542 |
|  |  |  | |  |
| **Web Of Science 1970 – present** | | | | |
|  |  |  | |  |
|  | Set | Results | |  |
|  | # 10 | #9 AND #8 AND #7 | | 3,006 |
|  |  | Indexes=SCI-EXPANDED, SSCI, A&HCI, CPCI-S, CPCI-SSH, ESCI Timespan=All years | |  |
|  | # 9 | #6 OR #2 | | 7,949,855 |
|  |  | Indexes=SCI-EXPANDED, SSCI, A&HCI, CPCI-S, CPCI-SSH, ESCI Timespan=All years | |  |
|  | # 8 | #4 OR #3 | | 578,684 |
|  |  | Indexes=SCI-EXPANDED, SSCI, A&HCI, CPCI-S, CPCI-SSH, ESCI Timespan=All years | |  |
|  | # 7 | #5 OR #1 | | 319,654 |
|  |  | Indexes=SCI-EXPANDED, SSCI, A&HCI, CPCI-S, CPCI-SSH, ESCI Timespan=All years | |  |
|  | # 6 | AB=("anxiety" OR "doubt*" OR "trust*" OR "intent*" OR "dilemma*" OR "attitude*" OR "distrust" OR "mistrust" OR "controvers*" OR "objector*" OR "awareness" OR "dropout*" OR "Perception*" OR "misconception*" OR "uptake" OR "behavi$r" OR "exemption*" OR "refus*" OR "misinformation" OR "barrier*" OR "belief*" OR "fear*" OR "reject*" OR "oppos*" OR "choice*" OR "criticis*" OR "hesitanc*" OR "rumo$r" OR "delay" OR "mandatory" OR "accept*" OR "concern*" OR "compulsory" OR "knowledge" OR "confiden*" OR "decision making" OR "anti-vaccin*" OR "parent*-confiden*") | | 7,019,756 |
|  |  | Indexes=SCI-EXPANDED, SSCI, A&HCI, CPCI-S, CPCI-SSH, ESCI Timespan=All years | |  |
|  | # 5 | AB = (vaccin* OR immunis* OR immuniz*) | | 234,860 |
|  |  | Indexes=SCI-EXPANDED, SSCI, A&HCI, CPCI-S, CPCI-SSH, ESCI Timespan=All years | |  |
|  | # 4 | AB= (maternal or antenatal* or prenatal* or pregnan*) | | 409,608 |
|  |  | Indexes=SCI-EXPANDED, SSCI, A&HCI, CPCI-S, CPCI-SSH, ESCI Timespan=All years | |  |
|  | # 3 | TI= (maternal or antenatal* or prenatal* or pregnan*) | | 321,154 |
|  |  | Indexes=SCI-EXPANDED, SSCI, A&HCI, CPCI-S, CPCI-SSH, ESCI Timespan=All years | |  |
|  | # 2 | TI = ("anxiety" OR "doubt*" OR "trust*" OR "intent*" OR "dilemma*" OR "attitude*" OR "distrust" OR "mistrust" OR "controvers*" OR "objector*" OR "awareness" OR "dropout*" OR "Perception*" OR "misconception*" OR "uptake" OR "behavi$r" OR "exemption*" OR "refus*" OR "misinformation" OR "barrier*" OR "belief*" OR "fear*" OR "reject*" OR "oppos*" OR "choice*" OR "criticis*" OR "hesitanc*" OR "rumo$r" OR "delay" OR "mandatory" OR "accept*" OR "concern*" OR "compulsory" OR "knowledge" OR "confiden*" OR "decision making" OR "anti-vaccin*" OR "parent*-confiden*") | | 1,810,146 |
|  |  | Indexes=SCI-EXPANDED, SSCI, A&HCI, CPCI-S, CPCI-SSH, ESCI Timespan=All years | |  |
|  | # 1 | TI = (vaccin* OR immunis* OR immuniz*) | | 181,085 |
|  |  | Indexes=SCI-EXPANDED, SSCI, A&HCI, CPCI-S, CPCI-SSH, ESCI Timespan=All years | |  |
|  |  |  | |  |
| **International Bibliography of the Social Sciences (IBSS)** | | | | |
|  | Set | Search | | Results |
|  |  |  | |  |
|  |  | Databases: | |  |
|  |  | International Bibliography of the Social Sciences (IBSS) | |  |
|  |  | These databases are searched for part of your query. | |  |
|  | S4 | ab((maternal or antenatal* or prenatal* or pregnan*)) OR ti((maternal or antenatal* or prenatal* or pregnan*)) Limits applied | | 18,165 |
|  |  | Peer reviewed:  Peer reviewed | |  |
|  |  | ab(anxiety OR doubt* OR trust* OR intent* OR dilemma* OR attitude* OR distrust OR mistrust OR controvers* OR objector* OR awareness OR dropout* OR Perception* OR misconception* OR uptake OR behavi?r OR exemption* OR refus* OR misinformation OR barrier* OR belief* OR fear* OR reject* OR oppos* OR choice* OR criticis* OR hesitanc* OR rumo?r OR delay OR mandatory OR accept* OR concern* OR compulsory OR knowledge OR confiden* OR "decision making" OR "anti-vaccin*" OR "parent*confiden*") OR ti(anxiety OR doubt* OR trust* OR intent* OR dilemma* OR attitude* OR distrust OR mistrust OR controvers* OR objector* OR awareness OR dropout* OR Perception* OR misconception* OR uptake OR behavi?r OR exemption* OR refus* OR misinformation OR barrier* OR belief* OR fear* OR reject* OR oppos* OR choice* OR criticis* OR hesitanc* OR rumo?r OR delay OR mandatory OR accept* OR concern* OR compulsory OR knowledge OR confiden* OR "decision making" OR "anti-vaccin*" OR "parent*-confiden*")Limits applied | | 722,897 |
|  |  | Narrowed by: | |  |
|  |  | Peer reviewed:  Peer reviewed | |  |
|  | S7 | ab(vaccin* or immunis* or immuniz*) OR ti(vaccin* or immunis* or immuniz*) Limits applied | | 3,214 |
|  |  | Databases: | |  |
|  |  | International Bibliography of the Social Sciences (IBSS) | |  |
|  | S2 | Narrowed by: | | 116 |
|  |  | Peer reviewed:  Peer reviewed | |  |
|  |  | S2 AND S7 AND S4 | |  |
|  |  |  | |  |
| Africa Wide | | | | |
|  | S1 AND S2 AND S3 | | | COMBINE |
|  | S3 | ab((vaccin* or immunis* or immuniz*)) OR ti((vaccin* or immunis* or immuniz*) | | Limiters - Abstract Available |
|  |  | Search modes - Boolean/Phrase | | |
|  | S2 | ab((anxiety OR doubt* OR trust* OR intent* OR dilemma* OR attitude* OR distrust OR mistrust OR controvers* OR objector* OR awareness OR dropout* OR Perception* OR misconception* OR uptake OR behavi?r OR exemption* OR refus* OR misinformation OR barrier* OR belief* OR fear* OR reject* OR oppos* OR choice* OR criticis* OR hesitanc* OR rumo?r OR delay OR mandatory OR accept* OR concern* OR compulsory OR knowledge OR confiden* OR "decision making" OR "anti-vaccin*" OR "parent*confiden*")) OR ti((anx ... | | Limiters - Abstract Available |
|  |  | Search modes - Boolean/Phrase | | |
|  | S1 | ab((maternal or antenatal* or prenatal* or pregnan*)) OR ti((maternal or antenatal* or prenatal* or pregnan*)) | | Limiters - Abstract Available |
|  |  |  | |  |
| Global Health 1910 to 2018 Week 45 | | | | |
|  | 1 | (vaccin* or immunis* or immuniz*).ti,ab. | | 155,961 |
|  | 2 | (anxiety or doubt* or trust* or intent* or dilemma* or attitude* or distrust or mistrust or controvers* or objector* or awareness or dropout* or Perception* or misconception* or uptake or behavio?r or exemption* or refus* or misinformation or barrier* or belief* or fear* or reject* or oppos* or choice* or criticis* or hesitanc* or rumo?r or delay or mandatory or accept* or concern* or compulsory or knowledge or confiden* or decision making or anti-vaccin* or parent*-confiden*).ti,ab. | | 691,825 |
|  | 3 | (maternal or antenatal* or prenatal* or pregnan*).ti,ab. | | 138,945 |
|  | 4 | Vaccines/ | | 67,879 |
|  | 5 | immunization/ | | 76,240 |
|  | 6 | vaccination/ | | 71,932 |
|  | 7 | attitudes/ | | 46,098 |
|  | 8 | knowledge/ | | 21,039 |
|  | 9 | behaviour/ | | 74,415 |
|  | 10 | decision making/ | | 6,373 |
|  | 11 | anxiety/ | | 9,484 |
|  | 12 | attitudes/ | | 46,098 |
|  | 13 | awareness/ | | 6,015 |
|  | 14 | perception/ | | 3,174 |
|  | 15 | maternal behaviour/ | | 346 |
|  | 16 | 1 or 4 or 5 or 6 | | 164,031 |
|  | 17 | 2 or 7 or 8 or 9 or 10 or 11 or 12 or 13 or 14 | | 733,909 |
|  | 18 | 3 or 15 | | 139,011 |
|  | 19 | 16 and 17 and 18 | | 2,799 |
|  |  |  | |  |
| **Scopus - Not included - as covered by EMBASE AND MEDLINE** | | | | |
|  | History Count | Search Terms | | Results |
|  | 10 | ( ( TITLE ( vaccin*  OR  immunis*  OR  immuniz* )  OR  ABS ( vaccin*  OR  immunis*  OR  immuniz* ) ) )  AND  ( ( TITLE ( maternal  OR  antenatal  OR  prenatal  OR  pregnan* )  OR  ABS ( maternal  OR  antenatal  OR  prenatal  OR  pregnan* ) ) )  AND  ( ( TITLE-ABS-KEY ( belief*  OR  fear*  OR  reject*  OR  oppos*  OR  choice*  OR  criticis*  OR  hesitanc*  OR  rumo?r  OR  delay  OR  mandatory  OR  accept*  OR  concern*  OR  compulsory  OR  knowledge  OR  confiden*  OR  decision  AND making  OR  anti-vaccin*  OR  parent*-confiden* )  OR  TITLE-ABS KEY ( anxiety  OR  doubt*  OR  trust*  OR  intent*  OR  dilemma*  OR  attitude*  OR  distrust  OR  mistrust  OR  controvers*  OR  objector*  OR  awareness  OR  dropout*  OR  perception*  OR  misconception*  OR  uptake  OR  behavio?r  OR  exemption*  OR  refus*  OR  misinformation  OR  barrier* ) ) ) ...View More | | 2,467 document results |
|  | 9 | ( TITLE-ABS-KEY ( belief*  OR  fear*  OR  reject*  OR  oppos*  OR  choice*  OR  criticis*  OR  hesitanc*  OR  rumo?r  OR  delay  OR  mandatory  OR  accept*  OR  concern*  OR  compulsory  OR  knowledge  OR  confiden*  OR  decision  AND making  OR  anti-vaccin*  OR  parent*-confiden* )  OR  TITLE-ABS-KEY ( anxiety  OR  doubt*  OR  trust*  OR  intent*  OR  dilemma*  OR  attitude*  OR  distrust  OR  mistrust  OR  controvers*  OR  objector*  OR  awareness  OR  dropout*  OR  perception*  OR  misconception*  OR  uptake  OR  behavio?r  OR  exemption*  OR  refus*  OR  misinformation  OR  barrier* ) ) | | 5,640,859 document results |
|  | 5 | ( TITLE ( maternal  OR  antenatal  OR  prenatal  OR  pregnan* )  OR  ABS ( maternal  OR  antenatal  OR  prenatal  OR  pregnan* ) ) | | 795,267 document results |
|  | 4 | ( TITLE ( vaccin*  OR  immunis*  OR  immuniz* )  OR  ABS ( vaccin*  OR  immunis*  OR  immuniz* ) ) | | 432,362 document results |
|  |  |  | |  |
| **LILACS** | | (title abstract subject = tw) | |  |
| **VHL Regional Portal** | | (tw:(vaccin$ or immunis$ or immuniz$)) AND (tw:(maternal or antenatal$ or prenatal$ or pregnan$)) AND (mj:(attitude OR trust OR anxiety OR decision-making OR choice-behavior OR Knowledge)) | | 31 results |
|  |  |  | |  |
| **Information and Knowledge for Health** | | | |  |
|  |  |  | |  |
| **IMEMR** | | | | |
|  |  | vaccin$ OR immunis$ OR immuniz$ [KeyWords] and maternal [KeyWords] and confidence [KeyWords] | | 6 articles generated all articles excluded for lack of relevance |
|  |  | vaccin$ OR immunis$ OR immuniz$ [Subject] and anxiety OR doubt$ OR trust$ OR intent$ OR dilemma$ OR attitude$ OR distrust OR mistrust OR controvers$ OR objector* OR awareness OR dropout$ OR Perception$ OR misconception$ OR uptake OR behavior OR beahviour OR exemption$ OR refus$ OR misinformation OR barrier$ OR belief$ OR fear$ OR reject$ OR oppos$ OR choice$ OR criticis$ OR hesitanc$ OR rumour OR rumor OR rumours OR rumors OR delay OR mandatory OR accept$ OR concern$ OR compulsory OR knowledge OR confiden$ OR decision making OR anti-vaccin$ OR “parent*confiden$" [Subject] and maternal or pregnancy [Subject] | | 0 articles |

| **EXTRACTION INTO ENDNOTE OCCURRED 22.11.18 at 13:04 via RIS files** | | | |
| --- | --- | --- | --- |
|  | **ris documents from database** | **Data Inserted** | **Total In Database** |
| 13:04 | Web Of Science | 3006 | **3,006** |
| 13:05 | Africa-Wide | 23 | **3,029** |
| 13:08 | CINAHL-Plus | 1500 | **4,529** |
| 13:16 | Embase Classic & Embase | 5690 | **10,219** |
| 13:20 | Global Health | 2799 | **13,018** |
| 14:05 | IBSS | 116 | **13,134** |
| 14.07 | LILACS | 31 | **13,165** |
| 14:43 | Medline | 3729 | **16,894** |
| 15:15 | PscyhInfo | 342 | **17,236** |
| - | IMEMR | 0 | **17,236** |

# **Appendix 5. Reasons for exclusion**

After screening all articles by title and abstract, 209 articles were screened by full text. 89 articles were excluded. The following table documents the reason for exclusion.

| **Reviewed by full text** | **Exclude Rationale** | **Detail** |
| --- | --- | --- |
| Abbott D, Morton K. H1N1 vaccine in pregnancy: The response so far. Archives of Disease in Childhood: Fetal and Neonatal Edition. 2010;95(SUPPL. 1)^2^ | Abstract |  |
| Adeyemi AB, Enabor OO, Ugwu IA, Bello FA, Olayemi OO. Knowledge of hepatitis B virus infection, access to screening and vaccination among pregnant women in Ibadan, Nigeria. 2013. p. 155-9.^3^ | Not about beliefs or experiences | Knowledge only, not about beliefs or attitudes of vaccination |
| Ahluwalia IB, Singleton JA, Jamieson DJ, Rasmussen SA, Harrison L. Seasonal Influenza Vaccine Coverage Among Pregnant Women: Pregnancy Risk Assessment Monitoring System. JOURNAL OF WOMENS HEALTH. 2011 2011/05//;20(5):649-51.^4^ | Report |  |
| Armitage EP, Camara J, Bah S, Forster AS, Clarke E, Kampmann B, et al. Acceptability of intranasal live attenuated influenza vaccine, influenza knowledge and vaccine intent in The Gambia. Vaccine. 2018;36(13):1772-80.^5^ | Incorrect population |  |
| Arriola CS, Vasconez N, Thompson M, Mirza S, Moen AC, Bresee J, et al. Factors associated with a successful expansion of influenza vaccination among pregnant women in Nicaragua. VACCINE. 2016 2016/02/17/;34(8):1086-90.^6^ | Incorrect outcome | Correlated sociodemographic factors with uptake of vaccine |
| Ashby M, Roussos-Ross D, De Cesare HPJ, Vidrine S, Floy E. Barriers for Tdap vaccination in pregnancy. Obstetrics and Gynecology. 2017;129(Supplement 1):40S.^7^ | Abstract |  |
| Ashelby LC, Trinder J. Swine flu vaccination: Why won't women have it? Archives of Disease in Childhood: Fetal and Neonatal Edition. 2011;96(SUPPL. 1).^8^ | Abstract |  |
| Azizi FSM, Kew Y, Moy FM. Vaccine hesitancy among parents in a multi-ethnic country, Malaysia. VACCINE. 2017 2017/05/19/;35(22):2955-61.^9^ | Incorrect outcome | Pregnant women views on childhood vaccination |
| Ballas J, Sachs M, Lee W, Espinoza J, Ramin S, Aagaard K, et al. Predictors and barriers to influenza and tdap vaccine uptake among women receiving prenatal care within an urban county hospital system. AMERICAN JOURNAL OF OBSTETRICS AND GYNECOLOGY. 2015 2015/01//;212(1, S):S289-S90.^10^ | Abstract |  |
| Barrett T, McEntee E, O'Shea A, Cleary B, Drew R, Collins C, et al. Influenza vaccination during pregnancy: Prevalence and barriers to uptake? European Journal of General Practice. 2017;23(1):148.^11^ | Abstract |  |
| Beel ER, Rench MA, Montesinos DP, Mayes B, Healy CM. Knowledge and attitudes of postpartum women toward immunization during pregnancy and the peripartum period. HUMAN VACCINES & IMMUNOTHERAPEUTICS. 2013 2013/09//;9(9):1926-31.^12^ | Incorrect outcome | About attitudes and beliefs toward peripartum immunisations |
| Betsch C, Boedeker B, Schmid P, Wichmann O. How baby's first shot determines the development of maternal attitudes towards vaccination. VACCINE. 2018 2018/05/17/;36(21):3018-26.^13^ | Incorrect outcome | Response to childhood vaccines or vaccines in general |
| Blanchard-Rohner G, Meier S, Ryser J, Schaller D, Burton-Jeangros C, de Tejada MB, et al. A study on the acceptability of the influenza vaccine during pregnancy. SWISS MEDICAL WEEKLY. 2011 2011/08/13/;141(187):34S.^14^ | Incorrect Reference |  |
| Bushar JA, Kendrick JS, Ding H, Black CL, Greby SM. Text4baby Influenza Messaging and Influenza Vaccination Among Pregnant Women. AMERICAN JOURNAL OF PREVENTIVE MEDICINE. 2017 2017/12//;53(6):845-53.^15^ | Not about beliefs or experiences | Not about beliefs, just healthcare providor recommendation and coverage |
| Calvo A, Gonzalez R, Pascale JM, Hess-Holtz M, Kaydos-Daniels SC, Azziz-Baumgartner E, et al. Influenza vaccine knowledge and acceptability. American Journal of Tropical Medicine and Hygiene. 2017;97(5 Supplement 1):217.^16^ | Abstract |  |
| Chamberlain AT, Seib K, Ault KA, Rosenberg ES, Frew PM, Cortes M, et al. Impact of a multi-component antenatal vaccine promotion package on improving knowledge, attitudes and beliefs about influenza and Tdap vaccination during pregnancy. HUMAN VACCINES & IMMUNOTHERAPEUTICS. 2016 2016;12(8):2017-24.^17^ | Intervention Study | Bias in reports as already agreed to be part of an educational program |
| Chan OK, Lao TT, Suen SS, Lau TK, Leung TY. Knowledge on hepatitis B infection among pregnant women in a high endemicity area. Patient Education & Counseling. 2011;85(3):516-20.^18^ | Incorrect outcome | Belief that HepB can be prevented by vaccination but not maternal vaccination) |
| Chan O, Suen S, Lao T, Leung K, Yeung S, Leung T. Determinants of hepatitis B vaccine uptake among pregnant Chinese women in Hong Kong. International Journal of Gynecology & Obstetrics. 2009;106(3):232-5.^19^ | Not about beliefs or experiences |  |
| Corben P, Leask J. Vaccination hesitancy in the antenatal period: a cross-sectional survey. BMC PUBLIC HEALTH. 2018 2018/05/02/;18.^20^ | Incorrect outcome | Not about vaccines in pregnancy |
| Danchin MH, Costa-Pinto J, Attwell K, Willaby H, Wiley K, Hoq M, et al. Vaccine decision-making begins in pregnancy: Correlation between vaccine concerns, intentions and maternal vaccination with subsequent childhood vaccine uptake. Vaccine. 2018;36(44):6473-9.^21^ | Incorrect outcome | Childhood vaccine concerns with maternal vaccine uptake coverage |
| Dempsey AF, Pyrzanowski J, Donnelly M, Brewer S, Barnard J, Beaty BL, et al. Acceptability of a hypothetical group B strep vaccine among pregnant and recently delivered women. VACCINE. 2014 2014/05/01/;32(21):2463-8.^22^ | Incorrect outcome | One question on Vaccines are safe in pregnancy in general |
| Deshpande M, Samin K, Banu N, Armstrong M. Factors affecting uptake of the seasonal influenza vaccine in the pregnant women. BJOG-AN INTERNATIONAL JOURNAL OF OBSTETRICS AND GYNAECOLOGY. 2013 2013/06//;120(1, SI):417.^23^ | Abstract |  |
| Dickin KL, Binchan RK, Purdue SE, Obinya EA. Perceptions of Neonatal Tetanus and Immunization During Pregnancy: A report of Focus Group Discussions in Kaduna, Nigeria. International Quarterly of Community Health Education. 1990 01/01/;11(4):371-83.^24^ | Incorrect population | Does not specify date to last birth |
| Dow-Clarke RA, MacCalder L, Hessel PA. Health behaviours of pregnant women in Fort McMurray, Alberta. Canadian Journal of Public Health. 1994;85(1):33-6.^25^ | Not about beliefs or experiences | Not about beliefs of maternal vaccinations |
| Du Y, He H, Zhu X. Investigation and analysis on the willingness for receiving influenza A(H1N1) vaccine among focus groups in Qingyuan city. Journal of Tropical Medicine (Guangzhou). 2010;10(6):738-40.^26^ | Abstract |  |
| Edet EE, Ikpeme BM, Ndifon WO, Oyo-Ita AE. Factors associated with missed opportunities to immunise with tetanus toxoid at a tertiary health institution in Nigeria. The Central African journal of medicine. 1998;44(8):199-202.^27^ | Incorrect outcome | Vaccination before pregnancy |
| Fell DB, Yasseen AS, Sprague AE, Walker M, Wen SW, Liu N, et al. Characteristics of women who received influenza vaccination in a population-based cohort of pregnant women in ontario. American Journal of Epidemiology. 2011;173(SUPPL. 11):S42.^28^ | Abstract |  |
| Ferdinand A. Boosting uptake of flu vaccination in pregnant women. Practice Nurse. 2012;42(15):12-4.^29^ | Report | Not original research on attitudes to maternal vaccination |
| Ferndale D, Meuter RFI, Watson B, Gallois C. 'You don't know what's going on in there': a discursive analysis of midwifery hospital consultations. Health, Risk and Society. 2017;19(7-8):411-31.^30^ | Not about beliefs or experiences | Midwifes dissemination of vaccination knowledge |
| Ford AJ, Alwan NA. Use of social networking sites and women's decision to receive vaccinations during pregnancy: A cross-sectional study in the UK. VACCINE. 2018 2018/08/23/;36(35):5294-303.^31^ | Incorrect outcome | Not about beliefs just use of social media websites |
| Frew PM, Owens LE, Saint-Victor DS, Benedict S, Zhang S, Omer SB. Factors associated with maternal influenza immunization decision-making Evidence of immunization history and message framing effects. HUMAN VACCINES & IMMUNOTHERAPEUTICS. 2014 2014/09//;10(9):2576-83.^32^ | Intervention Study |  |
| Gazmararian JA, Orenstein W, Prill M, Hitzhusen HB, Coleman MS, Pazol K, et al. Maternal Knowledge and Attitudes Toward Influenza Vaccination: A Focus Group Study in Metropolitan Atlanta. CLINICAL PEDIATRICS. 2010 2010/11//;49(11):1018-25.^33^ | Incorrect population | Mothers of Children (not under <48 months) and Influenza vaccination in general |
| Haile Z, Chertok I, Teweldeberhan A. Determinants of Utilization of Sufficient Tetanus Toxoid Immunization During Pregnancy: Evidence from the Kenya Demographic and Health Survey, 2008-2009. Journal of Community Health. 2013;38(3):492-9.^34^ | Not about beliefs or experiences |  |
| Holbeach N, McCarthy E, Howat P. Influenza vaccination during pregnancy 2014: Cohort of new mothers in a Melbourne level II hospital. Journal of Paediatrics and Child Health. 2015;51(SUPPL. 1):122.^35^ | Abstract |  |
| Howland R, Lu E, Diop H. Influenza vaccination among pregnant women - Massachusetts, 2009-2010. Morbidity and Mortality Weekly Report. 2013;62(43):854-7.^36^ | Report |  |
| Hu Y, Chen Y, Wang Y, Song Q, Li Q. Prenatal vaccination education intervention improves both the mothers' knowledge and children's vaccination coverage: Evidence from randomized controlled trial from eastern China. HUMAN VACCINES & IMMUNOTHERAPEUTICS. 2017 2017;13(6):1477-84.^37^ | Intervention Study |  |
| Johm P, Jaiteh F, Clarke E, Grietens KP. Understanding maternal and neonatal vaccination acceptance in the Gambia. TROPICAL MEDICINE & INTERNATIONAL HEALTH. 2017 2017/10//;22(1, SI):342-3.^38^ | Abstract |  |
| Jones TF, Ingram LA, Craig AS, Schaffner W. Determinants of influenza vaccination, 2003-2004: Shortages, fallacies and disparities. CLINICAL INFECTIOUS DISEASES. 2004 2004/12/15/;39(12):1824-8.^39^ | Not about beliefs and experiences | Beliefs not stratified by pregnant participants just coverage |
| Kashiba Y. Cross sectional study on factors associating neonatal tetanus immunization among reproductive aged women in Kasur District of Punjab Province, Pakistan; mothers' perceived belief on neonatal tetanus and tetanus toxoid vaccination. Journal of the National Institute of Public Health. 2007;56(4):418-9.^40^ | Abstract |  |
| Kelly E, Power M, Samelson R, Schulkin J. Factors associated with influenza vaccination proportion during pregnancy. Obstetrics and Gynecology. 2016;127(Supplement 1):57S.^41^ | Abstract |  |
| Kennedy ED, Ahluwalia IB, Ding H, Lu PJ, Singleton JA, Bridges CB. Monitoring seasonal influenza vaccination coverage among pregnant women in the United States. American Journal of Obstetrics & Gynecology. 2012;207(3):S9-S16.^42^ | Not about beliefs or experiences |  |
| Kim A, Schiller A, Mellon M, Nelson AL, Stohl HE. Tdap vaccine knowledge and acceptance rates during pregnancy in a los angeles public teaching-hospital clinic. Obstetrics and Gynecology. 2018;131(Supplement 1):175S.^43^ | Abstract |  |
| King CL, Chow MY, Leask J, Wiley KE. Australian caregivers' perceptions of influenza vaccination in pregnancy: A mixed methods exploration. Women and Birth. 2018.^44^ | Wrong population | Exclude as age group of children undefined. Caregivers of children (unclear if >2 years) |
| Kowal SP, Jardine CG, Bubela TM. “If they tell me to get it, I'll get it. If they don't ... “: Immunization decision-making processes of immigrant mothers. CANADIAN JOURNAL OF PUBLIC HEALTH-REVUE CANADIENNE DE SANTE PUBLIQUE. 2015 2015/06//MAY;106(4):E230-E5.^45^ | Wrong population |  |
| Kumar A, Unnikrishnan B, Rekha T, Mithra P, Kumar N, Kulkarni V, et al. Awareness and Attitude Regarding Breastfeeding and Immunization Practices Among Primigravida Attending a Tertiary Care Hospital in Southern India. JOURNAL OF CLINICAL AND DIAGNOSTIC RESEARCH. 2015 2015/03//;9(3):LC01-LC5.^46^ | Wrong outcome | Wrong outcome |
| Lo CY, Bedford H, Winyard P, Palit V. Pregnant women's views and experiences of pertussis vaccination during pregnancy: A study in taiwan. Archives of Disease in Childhood. 2018;103(Supplement 1):A178.^47^ | Abstract |  |
| Lu AB, Halim AA, Dendle C, Kotsanas D, Giles ML, Wallace EM, et al. Influenza vaccination uptake amongst pregnant women and maternal care providers is suboptimal. 2012. p. 4055-9.^48^ | Not about beliefs or experiences | Not beliefs |
| Lupton D. 'The best thing for the baby': mothers' concepts and experiences related to promoting their infants' health and development. Health, Risk & Society. 2011;13(7/8):637-51.^49^ | Wrong outcome | Childhood vaccine |
| Mak DB, Joyce S, Regan AK, Saker R, Jones C, Gibbs R, et al. ANTENATAL INFLUENZA VACCINE UPTAKE INCREASES BY 60% BETWEEN 2012 AND 2013. INTERNAL MEDICINE JOURNAL. 2014 2014/05//;44(3, SI):36-7.^50^ | Incorrect Reference |  |
| Massimi A, Rosso A, Marzuillo C, Vacchio MR, De Vito C, Villari P. Vaccine hesitancy: old defeat or new challenge for public health? A survey on pregnant women in Rome. EUROPEAN JOURNAL OF PUBLIC HEALTH. 2017 2017/11//;27(3).^51^ | Abstract |  |
| McAuslane H, Andrews N, Coole L, Wensley A. A survey of maternal pertussis vaccine uptake in England. INTERNATIONAL JOURNAL OF INFECTIOUS DISEASES. 2014 2014/04//;21(1):434.^52^ | Abstract |  |
| McCarthy CM, Arya A. Swine flu vaccination: Uptake and determinants of vaccination in pregnant women and Hospital staff in Ireland. Archives of Disease in Childhood: Fetal and Neonatal Edition. 2010;95(SUPPL. 1).^53^ | Abstract |  |
| McCarthy EA, Tapper L, Pollock WE, Sommerville M. Influenza vaccination in pregnancy after the 2009 pandemic: Experience in a Melbourne teaching hospital 2010 to 2014. BJOG: An International Journal of Obstetrics and Gynaecology. 2015;122(SUPPL. 2):289. ^54^ | Abstract |  |
| McQuaid F, Jones C, Stevens Z, Plumb J, Hughes R, Bedford H, et al. Factors influencing women's attitudes towards antenatal vaccines, group B Streptococcus and clinical trial participation in pregnancy: an online survey. BMJ OPEN. 2016 2016;6(4).^55^ | Wrong population |  |
| McSwiney C, Gibson L. A Survey of the uptake of the pertussis (Tdap) vaccination and awareness among mothers of infants (0-24months) in Cork University Hospital (CUH), and among staff of an antenatal clinic in Cork University Maternity Hospital (CUMH). IRISH JOURNAL OF MEDICAL SCIENCE. 2017 2017/06//;186(6):S173-S4.^56^ | Abstract |  |
| Moniz MH, Vitek WS, Akers A, Meyn LA, Beigi RH. Perceptions and Acceptance of Immunization During Pregnancy. JOURNAL OF REPRODUCTIVE MEDICINE. 2013 2013/10//SEP;58(9-10):383-8.^57^ | Unavailable |  |
| Moukarram H, Nargund A, Photiou A, Kiran TSU. Awareness and acceptance of the pandemic influenza (H1N1v 2009) vaccination among antenatal patients in a district general hospital. JOURNAL OF OBSTETRICS AND GYNAECOLOGY. 2012 2012/08//;32(6):537-9.^58^ | Not about beliefs or experiences | Not about beliefs or attitudes |
| Murphy K, McGeer A, De Souza L, Yudin M, Moore S. Knowledge about H1N1 influenza and the vaccine impacts vaccination uptake among pregnant women. AMERICAN JOURNAL OF OBSTETRICS AND GYNECOLOGY. 2011 2011/01//;204(1):S255.^59^ | Abstract |  |
| Naidu MA, Krishnaswamy S, Wallace EM, Giles ML. Pregnant women's attitudes toward antenatal pertussis vaccination. AUSTRALIAN & NEW ZEALAND JOURNAL OF OBSTETRICS & GYNAECOLOGY. 2017 2017/04//;57(2):235.^60^ | Letter |  |
| Nassar A, Usta I, Awwad J, Ghulmiyyah L, Khoury S, Ghazeeri G. Attitudes of women towards influenza a (H1N1) vaccination during pregnancy. Journal of Maternal-Fetal and Neonatal Medicine. 2010;23(SUPPL. 1):389.^61^ | Abstract |  |
| Nawa N, Kogaki S, Takahashi K, Ishida H, Baden H, Katsuragi S, et al. Analysis of public concerns about influenza vaccinations by mining a massive online question dataset in Japan. VACCINE. 2016 2016/06/08/;34(27):3207-13.^62^ | Not about beliefs or experiences | Questions from women not their views |
| Odumosu MO, Odumosu MO. Mass media and immunization awareness of pregnant women in a Nigerian community. Canadian Journal of Public Health. 1982;73:105-8.^63^ | Wrong population | Not pregnant women |
| O'Leary ST, Pyrzanowski J, Brewer SE, Barnard J, Beaty B, Donnelly M, et al. Influenza and Pertussis Vaccination Among Pregnant Women and Their Infants' Close Contacts Reported Practices and Attitudes. PEDIATRIC INFECTIOUS DISEASE JOURNAL. 2015 2015/11//;34(11):1244-9.^64^ | Wrong outcome | Cocooning perceptions |
| Panda B, Stiller R, Panda A. Influenza vaccination during pregnancy and factors for lacking compliance with current CDC guidelines. JOURNAL OF MATERNAL-FETAL & NEONATAL MEDICINE. 2011 2011/03//;24(3):402-6.^65^ | Intervention Study | Part of intervention |
| Patwardhan M, Ponce E, Rios Driscoll C, Gonik B. Global diversity in vaccine acceptance during pregnancy. American Journal of Obstetrics and Gynecology. 2015;213(6):894.^66^ | Abstract |  |
| Payakachat N, Hadden KB, Hanner J, Ragland D. Maternal knowledge of pertussis and Tdap vaccine and the use of a vaccine information statement. HEALTH EDUCATION JOURNAL. 2018 2018/04//;77(3):322-31.^67^ | Not about beliefs and experiences | Intervention/health literacy not beliefs or attitudes |
| Perry J, Towers CV, Weitz B, Wolfe L. Patient reaction to Tdap vaccination in pregnancy. VACCINE. 2017 2017/05/25/;35(23):3064-6.^68^ | Wrong outcome | Patient response to vaccination - adverse effects |
| Phoxay C, Okumura J, Nakamura Y, Wakai S. Influence of women's knowledge on maternal health care utilization in Southern Laos. Asia-Pacific Journal of Public Health. 2001;13(1):13-9.^69^ | Wrong population | Mother of children <5 |
| Pollock W, Hay S, McCarthy E, Nolan T, McDonald S. Pandemic h1n1 influenza vaccination uptake in pregnancy. Journal of Paediatrics and Child Health. 2011;47(SUPPL. 1):45-6.^70^ | Abstract |  |
| Ragland D, Hadden K, Payakachat N. Factors Associated with Maternal Knowledge of Pertussis and the Tdap Vaccine. JOURNAL OF WOMENS HEALTH. 2016 2016/04//;25(4):A28.^71^ | Abstract |  |
| Rahman M. Tetanus toxoid vaccination coverage and differential between urban and rural areas of Bangladesh. East African journal of public health. 2009;6(1):26-31.^72^ | Wrong population | All women asked (not pregnant or recently pregnant |
| Roosihermiatie B, Nishiyama M, Nakae K. Factors associated with TT (tetanus toxoid) immunization among pregnant women, in Saparua, Maluku, Indonesia. The Southeast Asian journal of tropical medicine and public health. 2000;31(1):91-5.^73^ | Not about beliefs or experiences | About knowledge unrelated to beliefs or experience |
| Rupali R, Pragati C, Saini NK, Kannan AT. Assessment of immunization coverage among infants and pregnant women in Narela using Lot quality assurance technique. Indian Journal of Public Health Research and Development. 2014;5(1):169-73.^74^ | Not about beliefs or experiences | Not about beliefs or attitudes |
| Sakaguchi S, Weitzner B, Carey N, Bozzo P, Koren G, Einarson A. Pregnant women and receiving the H1N1 vaccine: Perception of risk and determinants of decision-making. Journal of Population Therapeutics and Clinical Pharmacology. 2010;17(1):e227.^75^ | Abstract |  |
| Sasaki TK, Yoshida A, Kotake K. Attitudes about the 2009 H1N1 influenza pandemic among pregnant japanese women and the use of the japanese municipality as a source of information. Southeast Asian Journal of Tropical Medicine and Public Health. 2013;44(3):388-99.^76^ | Not about beliefs or experiences | Not beliefs or attitudes |
| Sevilla ME. Vaccination in pregnancy: Tetanus diphtheria and acellular pertussis (Tdap) immunization strategy at Hospital General de Ninos Dr Pedro de Elizalde 2013. Tropical Medicine and International Health. 2015;20(SUPPL. 1):278.^77^ | Abstract |  |
| Shafiq Y, Khowaja AR, Yousafzai MT, Ali SA, Zaidi A, Saleem AF. Knowledge, attitudes and practices related to tetanus toxoid vaccination in women of childbearing age: A cross-sectional study in peri-urban settlements of Karachi, Pakistan. Journal of Infection Prevention. 2017;18(5):232-41.^78^ | Wrong population | Results not stratified by pregnancy |
| Steiner B, Swamy GK, Walter EB. Engaging Expectant Parents to Receive Tdap Vaccination. AMERICAN JOURNAL OF PERINATOLOGY. 2014 2014/05//;31(5):407-12.^79^ | Wrong population |  |
| Stringer M, Ratcliff SJ, Gross R. Acceptance of hepatitis B vaccination by pregnant adolescents. MCN: The American Journal of Maternal Child Nursing. 2006;31(1):54-60.^80^ | Not about beliefs or experiences | Not about beliefs or attitudes |
| Thind A. Determinants of tetanus toxoid immunization in pregnancy in rural Bihar. TROPICAL DOCTOR. 2005 2005/04//;35(2):75-7.^81^ | Not about beliefs or experiences | Not about beliefs and attitudes |
| Vilca Yengle LM, Campins Marti M, Cabero Roura L, Rodrigo Pendas JA, Martinez Gomez X, Hermosilla Perez E, et al. Influenza vaccination in pregnant women. Coverage, practices and knowledge among obstetricians. MEDICINA CLINICA. 2010 2010/02/13/;134(4):146-51.^82^ | Wrong population | Provider beliefs |
| Vitek WS, Akers A, Meyn LA, Switzer GE, Lee BY, Beigi RH. Vaccine eligibility and acceptance among ambulatory obstetric and gynecologic patients. VACCINE. 2011 2011/03/03/;29(11):2024-8.^83^ | Not about beliefs or experiences | Does not explore beliefs or attitudes of pregnant women |
| Wang X, Qin W, Song W, Yue L, Zhu R, Liu H. Influence of Shandong illegal vaccine selling event on vaccination willingness of pregnant women. China Tropical Medicine. 2018;18(1):63-8.^84^ | Incorrect outcome | Parental confidence in childhood vaccination |
| Wilson RJ, Larson H, Paterson P. Understanding factors influencing vaccination acceptance during pregnancy in Hackney, London. LANCET. 2016 2016/11//;388(2):112.^85^ | Abstract |  |
| Winslade CG, Heffernan CM, Atchison CJ. Experiences and perspectives of mothers of the pertussis vaccination programme in London. PUBLIC HEALTH. 2017 2017/05//;146:10-4.^86^ | Incorrect population | Did not define participants (<2 years old - attended clinic) |
| Wong CY, Thomas NJ, Clarke M, Boros C, Tuckerman J, Marshall HS. Maternal uptake of pertussis cocooning strategy and other pregnancy related recommended immunizations. HUMAN VACCINES & IMMUNOTHERAPEUTICS. 2015 2015/05/04/;11(5):1165-72.^87^ | Incorrect population | Pre-pregnancy or postpartum |
| Yeager DP, Toy EC, Baker IB. Influenza vaccination in pregnancy. American Journal of Perinatology. 1999;16(6):283-6.^88^ | Abstract |  |
| Yudin MH, Salaripour M, Sgro MD. Acceptability and Feasibility of Seasonal Influenza Vaccine Administration in an Antenatal Clinic Setting. Journal of Obstetrics and Gynaecology Canada. 2010;32(8):745-8.^89^ | Not about beliefs or experiences |  |
| Yudin MH, Salripour M, Sgro MD. Impact of Patient Education on Knowledge of Influenza and Vaccine Recommendations Among Pregnant Women. Journal of Obstetrics and Gynaecology Canada. 2010;32(3):232-7.^90^ | Intervention Study | Patient knowledge leaflet given |

# **Appendix 6. PRISMA Checklist^91^**

| **Section/topic** | **#** | **Checklist item** | **Reported on page #, Appendix (App p)** |
| --- | --- | --- | --- |
| **TITLE** | | |  |
| Title | 1 | Identify the report as a systematic review, meta-analysis, or both. | 1 |
| **ABSTRACT** | | |  |
| Structured summary | 2 | Provide a structured summary including, as applicable: background; objectives; data sources; study eligibility criteria, participants, and interventions; study appraisal and synthesis methods; results; limitations; conclusions and implications of key findings; systematic review registration number. | 2 |
| **INTRODUCTION** | | |  |
| Rationale | 3 | Describe the rationale for the review in the context of what is already known. | 3-4 |
| Objectives | 4 | Provide an explicit statement of questions being addressed with reference to participants, interventions, comparisons, outcomes, and study design (PICOS). | 3-4 |
| **METHODS** | | |  |
| Protocol and registration | 5 | Indicate if a review protocol exists, if and where it can be accessed (e.g., Web address), and, if available, provide registration information including registration number. | 5 |
| Eligibility criteria | 6 | Specify study characteristics (e.g., PICOS, length of follow-up) and report characteristics (e.g., years considered, language, publication status) used as criteria for eligibility, giving rationale. | 4-5 |
| Information sources | 7 | Describe all information sources (e.g., databases with dates of coverage, contact with study authors to identify additional studies) in the search and date last searched. | 4-5 |
| Search | 8 | Present full electronic search strategy for at least one database, including any limits used, such that it could be repeated. | App p3, app p5-10 |
| Study selection | 9 | State the process for selecting studies (i.e., screening, eligibility, included in systematic review, and, if applicable, included in the meta-analysis). | 4-5 |
| Data collection process | 10 | Describe method of data extraction from reports (e.g., piloted forms, independently, in duplicate) and any processes for obtaining and confirming data from investigators. | 4-5, App p37-40, app p48 |
| Data items | 11 | List and define all variables for which data were sought (e.g., PICOS, funding sources) and any assumptions and simplifications made. | 4-5, App p2 |
| Risk of bias in individual studies | 12 | Describe methods used for assessing risk of bias of individual studies (including specification of whether this was done at the study or outcome level), and how this information is to be used in any data synthesis. | 5, App p18 |
| Summary measures | 13 | State the principal summary measures (e.g., risk ratio, difference in means). | 5 |
| Synthesis of results | 14 | Describe the methods of handling data and combining results of studies, if done, including measures of consistency (e.g., I^2^) for each meta-analysis. | 5 |
| Risk of bias across studies | 15 | Specify any assessment of risk of bias that may affect the cumulative evidence (e.g., publication bias, selective reporting within studies). | 5 |
| Additional analyses | 16 | Describe methods of additional analyses (e.g., sensitivity or subgroup analyses, meta-regression), if done, indicating which were pre-specified. | 5 |
| **RESULTS** | | |  |
| Study selection | 17 | Give numbers of studies screened, assessed for eligibility, and included in the review, with reasons for exclusions at each stage, ideally with a flow diagram. | 5-16, Figure 1 |
| Study characteristics | 18 | For each study, present characteristics for which data were extracted (e.g., study size, PICOS, follow-up period) and provide the citations. | Table 1 |
| Risk of bias within studies | 19 | Present data on risk of bias of each study and, if available, any outcome level assessment (see item 12). | App p19-29 |
| Results of individual studies | 20 | For all outcomes considered (benefits or harms), present, for each study: (a) simple summary data for each intervention group (b) effect estimates and confidence intervals, ideally with a forest plot. | Figure 2, App p90-91 |
| Synthesis of results | 21 | Present results of each meta-analysis done, including confidence intervals and measures of consistency. | 8-20 App p55-94 |
| Risk of bias across studies | 22 | Present results of any assessment of risk of bias across studies (see Item 15). | 18, |
| Additional analysis | 23 | Give results of additional analyses, if done (e.g., sensitivity or subgroup analyses, meta-regression [see Item 16]). | 18 App p90-94 |
| **DISCUSSION** | | |  |
| Summary of evidence | 24 | Summarize the main findings including the strength of evidence for each main outcome; consider their relevance to key groups (e.g., healthcare providers, users, and policy makers). | 16-20 |
| Limitations | 25 | Discuss limitations at study and outcome level (e.g., risk of bias), and at review-level (e.g., incomplete retrieval of identified research, reporting bias). | 15-20 |
| Conclusions | 26 | Provide a general interpretation of the results in the context of other evidence, and implications for future research. | 16-20 |
| **FUNDING** | | |  |
| Funding | 27 | Describe sources of funding for the systematic review and other support (e.g., supply of data); role of funders for the systematic review. | 21 |

# **Appendix 7. Expanded methodology**

***Search strategy and selection criteria:***

We searched MEDLINE, Embase Classic and Embase, PsycINFO, CINAHL Plus, Web of Science, IBSS, LILACS, AfricaWideInfo, IMEMR, and Global Health for studies published by 22 November 2018 (appendix p3-10). Additional studies were identified by screening reference lists (EK, SD) of previous reviews and suggestions by experts in the field. Three additional sources were identified through this method.

A rationale for the study population is outlined on p2 Appendix. A detailed inclusion and exclusion criteria is outline on p4 Appendix. Two authors (EK, SD) independently screened records by title and abstract after duplicates were removed. The two authors (EK, SD) met and agreed upon articles to be assessed for eligibility by full-text. This was done by reviewing the ‘include’ list of each author and discussing any discrepancies. If one author had included a record the other author had excluded the authors explained their decisions evaluated. A decision was reached by reviewing the inclusion and exclusion criteria against the article title. A third author (PP) reviewed any disagreements. However, if there was uncertainty the authors proceeded to review the article by full text.

The two authors (EK, SD) then reviewed the articles by full-text to determine final inclusion. The authors met again to discuss reasons for the exclusion of 89 records (ranging from incorrect exposure, outcome or population to non-peer reviewed records). Articles were then assessed for content and validity assessment.

***Quality appraisal:***

Quality appraisal was performed (EK, SD) using the Joanna Briggs Institute quality assessment tool’s separate checklists for cross-sectional, cohort, and qualitative research studies (appendix p19-30). This tool was chosen because it had separate checklists for each type of study (Appendix p19-30). Studies were ranked based on a framework developed by the framework with an attributed quality score. Points were awarded to each mark of quality: Yes = 2 No = -2 Unclear =-1 N/A = 0. Authors decided on a scoring system (Appendix p18) to assess if the paper was on low quality and to therefore exclude. Where authors disagreed on final point allocation, the arbiter (PP) intervened to resolve the disagreement.

***Data analysis:***

Two authors (EK, SD) extracted the factors influencing maternal vaccination into Microsoft Excel and developed a broad themed coding template using grounded theory to categorise factors influencing maternal vaccination uptake. Any factor noted in the full text as a possible contribution to vaccination decision making was extracted from the full text. For example, papers made reference in both quantitative and qualitative papers to social media, blogs, friends, other pregnant women, family, and professionals influencing their decision to vaccinate. This was combined to provide a framework to generate a broad factor “social influences on vaccine use”. Other contextual factors were isolated from this theme.

While there are many existing frameworks relating to vaccination uptake, we used this approach instead in order to identify factors specific to maternal vaccinations mentioned in the literature. The SAGE framework is widely used, however we regarded this as too narrow in exploring the emotional factors and the perceptions of risk. The model divides the influencers into three broad main categories (vaccine and vaccination specific issues, individual social and group influences and contextual factors). Given we wished to focus on maternal vaccination specifically, we hypothesized there may be factors which are broadly more important or potentially unidentified from the childhood vaccination literature given that the unborn foetus was *not considered as a primary factor* during the conceptualisation of the SAGE Working Group model. Additionally, maternal vaccination is an advancing specialisation with increased availability to vaccines during outbreaks (Ebola) and pandemics (Influenza). Therefore, the factors at play are potentially different to routinely administered well established childhood vaccines. Two authors (EK, SD) independently coded all articles using the developed codes (Manuscript Table 2) in NVivo12 (Kappa score 0.76).

***Eight Broad Categories:***

accessibility and convenience

personal values and lifestyle

awareness of information

social influences

emotions

perceptions of vaccine risk

perceptions of vaccine benefit

personal vaccination history

Using the broad codes, authors developed a sub-categorization of these codes after joint discussion for qualitative analysis. For “social influences on vaccine use” this was sub-categorised into the minimum number of subcategories that could incorporate all the factors but provide nuanced information (Kappa score 0.88). e.g.:

***Subcategory for “Social Influences”***

Trust in HCP

HCP as a source of information

HCP [did/ didn’t] recommend

HCP [did/didn’t] offer vaccine

Family/ friends

Media

Two authors (EK, MF) assessed the quantitative studies for inclusion in the meta-analysis. To be included in the meta-analysis, studies had to report an estimated odds ratio (OR/ could be calculated from raw data) for the association between a specific factor (from the first-cycle broad codes) and vaccination status (excluding intention to be vaccinated). In order to include that maximum data in meta-analysis each paper was evaluated against each broad code and the specific question asked in the survey or questionnaire was recorded along with the reference (EK). The broad category “perception of vaccine risk” was divided into “side effects” and “vaccine harm.” The broad category risk of disease was divided into “disease susceptibility” and “disease severity.” Data could not be synthesized for “accessibility and convenience” or “personal values and lifestyle.” This generated the broad categories for meta-analysis data synthesis:

***Broad Categories for Meta-analysis:***

Awareness and Information

Disease Severity

Disease Susceptibility

Vaccine Side Effects

Vaccine Harm

Benefits of vaccination

Healthcare Professional Recommendation

Personal History of Vaccination

After recording all the questions from the studies that fitted one of the broad 8 categories (above), synthesis of the exact questions occurred (appendix 11 p39) A detailed outline examining which questions were synthesized for meta-analysis is available in the Appendix 12 p 40. Therefore, the two authors developed 23 sub-codes based on the exact exposures measured in quantitative studies. The two authors (EK, MF) independently extracted the Odds ratios (or raw data) which fitted these codes. Extracted data from both data were then compared. Error were corrected and agreed by two authors. No discrepancies could not be resolved. Based on data available to be pooled, 31 separate meta-analyses were conducted.

***Meta-analysis methodology***

A meta-analysis was undertaken to quantify the relationship between individual sentiments or experiences and maternal vaccination uptake. We performed a random-effects meta-analyses, which accounts for “within study” and “between study” variability. This was decided *a priori* given expected clinical (differences in participant characteristics by geographic location) and methodological (differences in study quality) heterogeneity between studies. We calculated the Q statistic and the I^2^ index to assess the degree of heterogeneity present in our meta-analyses. Funnel plots were investigated to assess the potential for publication bias.

All statistical and graphical analyses were conducted using Stata 15.

***Secondary analyses***

We conducted a secondary analysis that meta-analysed studies reporting an intention to vaccinate outcome. The aim was to compare results to our primary analysis and investigate whether intention to vaccinate is a surrogate of actual vaccination status.

Furthermore, we conducted a sensitivity analysis restricting to studies with a JBI > 0, therefore including studies with the lowest risk of bias.

Figure 1: Process map of methodology

# **Appendix 8. Joanna Briggs Institute (JBI) Quality Assessment**

*We chose the Joanna Briggs Institute (JBI) Critical Appraisal Tool to assess the quality of each included study. The JBI tool was adopted because it provided the capability to appraise cohort, cross-sectional and qualitative studies.^92^ Exclusion of articles based on quality assessment was used in our pre-defined sensitivity analysis.*

**Scoring System**

Authors decided on a scoring system to assess if the paper was on low quality and to therefore exclude. Points were awarded to each mark of quality: Yes = 2 No = -2 Unclear =-1 N/A = 0 (Note: confounding factors stated here was adapted to include potential confounding factors found, given exploratory analysis to look for confounding factors was assessed as improving the quality of the paper). If a paper identified a confounding factor which was not dealt with (stratification) it was completely excluded.

Papers score > or including 0 include

Papers score < 0 exclude

score < 0 = very low; 0 ≤ score < 5 = low; 5 ≤ score < 10 = moderate; score ≥ 10 = strong

**JBI Ratings – Cohort Studies**

| **Study ID** | **Reviewer** | **Population** | **Exposure Group** | **Exposure Measure** | **Confounding Factors Identified** | **Confounding Factors Strategies** | **Free of Outcome at Start** | **Outcome Measure** | **Follow Up Time** | **Follow Up Complete** | **Follow Up Strategies** | **Analysis** | **Total** | **Agreed** |
| --- | --- | --- | --- | --- | --- | --- | --- | --- | --- | --- | --- | --- | --- | --- |
| **Boedeker *et al.* (2015)^93^** | EK | -1 | 2 | 2 | 2 | -2 | 0 | -1 | 2 | 2 | -1 | 2 | 7 | 7 (SR) |
|  | SD | -1 | 2 | 2 | -1 | 2 | 0 | -1 | -1 | -1 | -1 | 2 | 2 |  |
| **Henninger *et al.* (2013)^94^** | EK | 2 | 2 | 2 | 2 | 2 | -2 | -2 | 2 | 2 | 0 | 2 | 12 | 12 |
|  | SD | 2 | 2 | 2 | 2 | 2 | -2 | -2 | 2 | 2 | 0 | 2 | 12 |  |
| **Henninger *et al.* (2015)^95^** | EK | 2 | 2 | 2 | 2 | 2 | -2 | -2 | 2 | 2 | 0 | 2 | 12 | 12 |
|  | SD | 2 | 2 | 2 | 2 | 2 | -2 | -1 | 2 | 2 | 0 | 2 | 13 |  |
| **Ugezu *et al.* (2018)^96^** | EK | 2 | 2 | -2 | -2 | -2 | 2 | -2 | 2 | -1 | 2 | -1 | 0 | 0 |
|  | SD | 2 | 2 | -2 | -2 | -2 | 2 | -2 | 2 | -1 | 2 | -1 | 0 |  |

Key:

*Population:* Were the two groups similar and recruited from the same population?

*Exposure Group:* Were the exposures measured similarly to assign people to both exposed and unexposed groups?

*Exposure Measure:* Was the exposure measured in a valid and reliable way?

*Confounding Factors Identified:* Were confounding factors identified?

*Confounding Factors Strategies:* Were strategies to deal with confounding factors stated?

*Free of Outcome at Start:* Were the groups/participants free of the outcome at the start of the study (or at the moment of exposure)?

*Outcome Measure:* Were the outcomes measured in a valid and reliable way?

*Follow Up Time:* Was the follow up time reported and sufficient to be long enough for outcomes to occur?

*Follow Up Complete:* Was follow up complete, and if not, were the reasons to loss to follow up described and explored?

*Follow Up Strategies:* Were strategies to address incomplete follow up utilized?

*Analysis:* Was appropriate statistical analysis used?

**JBI Ratings – Cross-Sectional Studies**

Key:

*Sample Inclusion:* Were the criteria for inclusion in the sample clearly defined?

*Study Objects:* Were the study subjects and the setting described in detail? (the study was assessed for if it described the trimester or post partum period, the dates and specific setting of the study)

*Exposure Measure:* Was the exposure (belief/reason) measured in a valid and reliable way? (using a survey or questionnaire with details of the nature in which the survey was applied and details of all questions should be present)

*Condition:* Were objective, standard criteria used for measurement of the condition? (pregnancy)

*Confounding Factors Identified:* Were confounding factors identified? (mentioned in text or analysis)

*Confounding Factors Strategies:* Were strategies to deal with confounding factors stated? (multiple linear regression present)

*Outcome Measure:* Were the outcomes measured in a valid and reliable way? (1 point deducted if vaccination status not verified by medical record)

*Statistical Analysis:* Was appropriate statistical analysis used?

| **Study ID** | **Reviewer** | **Sample Inclusion** | **Study Subjects** | **Exposure Measure** | **Condition** | **Confounding Factors Identified** | **Confounding Factors Strategies** | **Outcome Measure** | **Statistical Analysis** | **Total** | **Agreed** |
| --- | --- | --- | --- | --- | --- | --- | --- | --- | --- | --- | --- |
| **Abasi *et al.* (2015)^97^** | EK | -1 | 2 | 2 | 0 | -2 | -2 | -1 | 2 | 0 | 3 |
|  | SD | 2 | 2 | 2 | 0 | -2 | -2 | -1 | 2 | 3 |  |
| **Agricola *et al.* (2016)^98^** | EK | 2 | 2 | 2 | 0 | 2 | 2 | 2 | 2 | 14 | 14 |
|  | SD | 2 | 2 | 2 | 0 | 2 | 2 | -1 | 2 | 11 |  |
| **Arriola *et al.* (2018)^99^** | EK | 2 | 2 | 2 | 0 | 2 | 2 | 2 | 2 | 14 | 14 (SR) |
|  | SD | 2 | 2 | 2 | 0 | 2 | -2 | -2 | 2 | 6 |  |
| **Ashfaq *et al.* (2017)^100^** | EK | 2 | 2 | -1 | 0 | -2 | -2 | -1 | -2 | -4 | -4 |
|  | SD | 2 | 2 | -1 | 0 | -2 | -2 | -1 | -2 | -4 |  |
| **Barrett *et al.* (2018)^101^** | EK | 2 | 2 | 2 | 0 | 2 | 2 | 2 | 2 | 14 | 10 |
|  | SD | 2 | 2 | 2 | 0 | 2 | 2 | -1 | 2 | 11 |  |
| **Beigi *et al.* (2009)^102^** | EK | -2 | -2 | 2 | 0 | -2 | 2 | 2 | 2 | 2 | 8 (SR) |
|  | SD | -1 | 2 | 2 | 0 | 2 | 2 | 2 | 2 | 11 |  |
| **Ben Natan *et al.* (2017)^103^** | EK | -1 | 2 | -1 | 0 | 2 | -2 | -2 | 2 | 0 | 0 |
|  | SD | -1 | 2 | -1 | 0 | -1 | -2 | -2 | 2 | -3 |  |
| **Bettinger *et al.* (2016)^104^** | EK | -2 | -1 | 2 | 0 | -2 | -2 | 2 | 2 | -1 | -1 |
|  | SD | -2 | -1 | 2 | 0 | -2 | -2 | 2 | 2 | -1 |  |
| **Bhaskar *et al.* (2012)^105^** | EK | -1 | 2 | 2 | 0 | -2 | -2 | -2 | 2 | -1 | -1 |
|  | SD | -1 | 2 | 2 | 0 | -2 | -2 | -2 | 2 | -1 |  |
| **Blanchard-Rohner *et al.* (2012)^106^** | EK | 2 | 2 | 2 | 0 | 2 | 2 | 2 | 2 | 14 | 14 |
|  | SD | 2 | 2 | 2 | 0 | 2 | 2 | 2 | 2 | 14 |  |
| **Blondel *et al.* (2012)^107^** | EK | -2 | 2 | -1 | 0 | 2 | 2 | 2 | 2 | 7 | 7 |
|  | SD | 2 | 2 | -1 | 0 | 2 | 2 | 2 | 2 | 11 |  |
| **Boedeker *et al.* (2014)^108^** | EK | 2 | 2 | 2 | 0 | 2 | 2 | 2 | 2 | 14 | 14 |
|  | SD | 2 | 2 | 2 | 0 | 2 | 2 | 2 | 2 | 14 |  |
| **Campbell *et al.* (2015)^109^** | EK | 2 | 2 | 2 | 0 | -2 | -2 | 2 | 2 | 6 | 6 |
|  | SD | 2 | 2 | 2 | 0 | -2 | -2 | 2 | 2 | 6 |  |
| **Castro-Sanchez *et al.* (2018)^110^** | EK | 2 | 2 | 2 | 0 | -2 | 2 | 2 | 2 | 10 | 14 |
|  | SD | 2 | 2 | 2 | 0 | 2 | 2 | 2 | 2 | 14 |  |
| **Celikel *et al.* (2014)^111^** | EK | 2 | 2 | 2 | 0 | -2 | -2 | 2 | -1 | 3 | 3 |
|  | SD | 2 | 2 | 2 | 0 | -2 | -2 | 2 | -1 | 3 |  |
| **Chamberlain *et al.* (2015)^112^** | EK | 2 | 2 | 2 | 0 | -2 | -2 | 2 | 2 | 6 | 11 |
|  | SD | 2 | 2 | 2 | 0 | -2 | -2 | 2 | 2 | 6 |  |
| **Chamberlain *et al.* (2016)^113^** | EK | 2 | 2 | 2 | 0 | 2 | 2 | -1 | 2 | 11 | 11 |
|  | SD | 2 | 2 | 2 | 0 | 2 | 2 | -1 | 2 | 11 |  |
| **D'Alessandro *et al.* (2018)^114^** | EK | 2 | 2 | 2 | 0 | -2 | 2 | 2 | 2 | 10 | 10 |
|  | SD | -2 | 2 | 2 | 0 | 2 | 2 | 2 | 2 | 10 |  |
| **Dempsey *et al.* (2016)^115^** | EK | 2 | 2 | 2 | 0 | 2 | 2 | 2 | 2 | 14 | 8 (SR) |
|  | SD | 2 | 2 | 2 | 0 | -2 | -2 | 2 | 2 | 6 |  |
| **Ding *et al.* (2011)^116^** | EK | 2 | 2 | 2 | 0 | -2 | 2 | -1 | 2 | 7 | 7 |
|  | SD | 2 | 2 | 2 | 0 | -2 | 2 | -1 | 2 | 7 |  |
| **Ditsungneon *et al.* (2016)^117^** | EK | 2 | 2 | 2 | 0 | 2 | 2 | 2 | 2 | 14 | 14 |
|  | SD | 2 | 2 | 2 | 0 | 2 | 2 | 2 | 2 | 14 |  |
| **Dlugacz *et al.* (2012)^118^** | EK | -2 | 2 | 2 | 0 | -1 | 2 | -2 | 2 | 3 | 3 |
|  | SD | -2 | 2 | 2 | 0 | -1 | 2 | -2 | 2 | 3 |  |
| **Donaldson *et al.* (2015)^119^** | EK | 2 | 2 | -1 | 0 | -1 | -2 | -2 | 2 | 0 | 0 |
|  | SD | 2 | 2 | -1 | 0 | -1 | -2 | -2 | 2 | 0 |  |
| **Drees *et al.* (2012)^120^** | EK | 2 | 2 | 2 | 0 | 2 | 2 | 2 | 2 | 14 | 14 |
|  | SD | 2 | 2 | 2 | 0 | 2 | 2 | 2 | 2 | 14 |  |
| **Drees *et al.* (2013)^121^** | EK | 2 | 2 | 2 | 0 | -1 | 2 | -1 | 2 | 8 | 11 |
|  | SD | 2 | 2 | 2 | 0 | 2 | 2 | -1 | 2 | 11 |  |
| **Edmonds *et al.* (2011)^122^** | EK | 2 | 2 | 2 | 0 | 2 | 2 | 2 | 2 | 14 | 14 |
|  | SD | 2 | 2 | 2 | 0 | 2 | 2 | 2 | 2 | 14 |  |
| **Eppes *et al.* (2013)^123^** | EK | 2 | 2 | 2 | 0 | -1 | -2 | -2 | 2 | 3 | 2 |
|  | SD | 2 | 2 | 2 | 0 | -2 | -2 | -2 | 2 | 2 |  |
| **Fabry *et al.* (2011)^124^** | EK | 2 | 2 | 2 | 0 | 2 | 2 | -1 | 2 | 11 | 11 |
|  | SD | 2 | 2 | 2 | 0 | 2 | 2 | -1 | 2 | 11 |  |
| **Fisher *et al.* (2011)^125^** | EK | 2 | 2 | -1 | 0 | -2 | -2 | -2 | 2 | -1 | -1 |
|  | SD | 2 | 2 | -1 | 0 | -2 | -2 | -2 | 2 | -1 |  |
| **Fleming *et al.* (2018)^126^** | EK | 2 | 2 | -2 | 0 | -2 | -2 | -2 | -1 | -5 | -5 |
|  | SD | 2 | 2 | -2 | 0 | -2 | -2 | -2 | -1 | -5 |  |
| **Fridman *et al.* (2011)^127^** | EK | 2 | 2 | 2 | 0 | 2 | 2 | -2 | 2 | 10 | 10 |
|  | SD | 2 | 2 | 2 | 0 | 2 | 2 | -2 | 2 | 10 |  |
| **Gaudelus *et al.* (2016)^128^** | EK | 2 | 0 | 2 | 0 | -2 | -2 | 2 | 2 | 4 | 1 |
|  | SD | 2 | 0 | -1 | 0 | -2 | -2 | 2 | 2 | 1 |  |
| **Goldfarb *et al.* (2011)^129^** | EK | -2 | 2 | 2 | 0 | -2 | -2 | -1 | 2 | -1 | -1 |
|  | SD | -2 | 2 | 2 | 0 | -2 | -2 | -1 | 2 | -1 |  |
| **Gorman *et al.* (2012)^130^** | EK | -2 | 2 | 2 | 0 | -1 | 2 | -1 | 2 | 4 | 4 |
|  | SD | -2 | 2 | 2 | 0 | -1 | 2 | -1 | 2 | 4 |  |
| **Gul *et al.* (2016)^131^** | EK | -1 | -2 | -2 | 0 | -2 | -2 | -1 | 0 | -10 | -9 |
|  | SD | -1 | -1 | -2 | 0 | -2 | -2 | -1 | 0 | -9 |  |
| **Hallisey *et al.* (2018)^132^** | EK | 2 | 2 | -1 | 0 | -2 | -2 | -2 | 0 | -3 | -3 |
|  | SD | 2 | 2 | -1 | 0 | -2 | -2 | -2 | 0 | -3 |  |
| **Halperin *et al.* (2014)^133^** | EK | 2 | 2 | 2 | 0 | 2 | 2 | -1 | 2 | 11 | 11 |
|  | SD | 2 | 2 | 2 | 0 | 2 | 2 | -1 | 2 | 11 |  |
| **Hasnain & Sheikh (2017)^134^** | EK | 2 | 2 | 2 | 0 | -2 | -2 | 2 | 2 | 6 | 6 |
|  | SD | 2 | 2 | 2 | 0 | -2 | -2 | 2 | 2 | 6 |  |
| **Hassan *et al.* (2016)^135^** | EK | -2 | 2 | 2 | 0 | -2 | -2 | 2 | 2 | 2 | -1 |
|  | SD | -2 | 2 | -1 | 0 | -2 | -2 | 2 | 2 | -1 |  |
| **Hayles *et al.* (2015)^136^** | EK | 2 | 2 | 2 | 0 | -2 | -2 | 2 | -1 | 3 | 3 |
|  | SD | 2 | 2 | 2 | 0 | -2 | -2 | 2 | -1 | 3 |  |
| **Healy *et al.* (2015)^137^** | EK | -1 | 2 | -1 | 0 | 2 | 2 | 2 | 2 | 8 | 8 |
|  | SD | 2 | 2 | -1 | 0 | 2 | 2 | -1 | 2 | 8 |  |
| **Hill *et al.* (2018)^138^** | EK | -1 | 2 | 2 | 0 | -2 | -2 | -2 | 2 | -1 | -1 |
|  | SD | -1 | 2 | 2 | 0 | -2 | -2 | -2 | 2 | -1 |  |
| **Honarvar *et al.* (2012)^139^** | EK | 2 | 2 | 2 | 0 | 2 | -1 | 2 | 2 | 11 | 8 |
|  | SD | -1 | 2 | 2 | 0 | 2 | -1 | 2 | 2 | 8 |  |
| **Hu *et al.* (2017)^140^** | EK | 2 | 2 | 2 | 0 | 2 | 2 | 2 | 2 | 14 | 14 |
|  | SD | 2 | 2 | 2 | 0 | 2 | 2 | 2 | 2 | 14 |  |
| **Jadoon *et al.* (2017)^141^** | EK | 2 | 2 | -2 | 0 | -2 | -2 | -2 | -1 | -5 | -5 |
|  | SD | 2 | 2 | -2 | 0 | -2 | -2 | -2 | -1 | -5 |  |
| **Kang *et al.* (2015)^142^** | EK | -2 | 2 | 2 | 0 | -2 | -2 | 2 | 2 | 2 | 2 |
|  | SD | -2 | 2 | 2 | 0 | -2 | -2 | 2 | 2 | 2 |  |
| **Kay *et al.* (2012)^143^** | EK | 2 | 2 | -1 | 0 | 2 | 2 | -2 | 2 | 7 | 7 |
|  | SD | 2 | 2 | -1 | 0 | 2 | 2 | -1 | 2 | 8 |  |
| **Kfouri *et al.* (2013)^144^** | EK | -2 | -1 | -2 | 0 | -2 | -2 | -2 | -1 | -12 | -12 |
|  | SD | -2 | 2 | -2 | 0 | -2 | -2 | -2 | -1 | -9 |  |
| **Khan *et al.* (2015)^145^** | EK | 2 | 2 | 2 | 0 | -2 | -2 | 2 | 2 | 6 | 6 |
|  | SD | 2 | 2 | 2 | 0 | -1 | -2 | 2 | 2 | 7 |  |
| **Kouassi *et al.* (2012)^146^** | EK | 2 | 2 | -1 | 0 | 2 | 2 | 2 | 2 | 11 | 11 |
|  | SD | -2 | 2 | -1 | 0 | 2 | 2 | 2 | 2 | 7 |  |
| **Koul *et al.* (2014)^147^** | EK | -2 | 2 | 2 | 0 | -2 | -2 | -1 | 2 | -1 | -4 |
|  | SD | -1 | 2 | -1 | 0 | -2 | -2 | -1 | 2 | -3 |  |
| **Krishnaswamy *et al.* (2018)^148^** | EK | 2 | 2 | -1 | 0 | 2 | 2 | -1 | 2 | 8 | 8 |
|  | SD | 2 | 2 | -1 | 0 | 2 | 2 | -1 | 2 | 8 |  |
| **Kriss *et al.* (2018)^149^** | EK | 2 | 2 | 2 | 0 | 2 | 2 | 2 | 2 | 14 | 14 |
|  | SD | 2 | 2 | -1 | 0 | 2 | 2 | 2 | 2 | 11 |  |
| **Lau *et al.* (2010)^150^** | EK | 2 | 2 | 2 | 0 | 2 | 2 | -2 | 2 | 10 | 10 |
|  | SD | -1 | 2 | 2 | 0 | 2 | 2 | -1 | 2 | 8 |  |
| **Lotter *et al.* (2018)^151^** | EK | 2 | 2 | 2 | 0 | 2 | 2 | 2 | 2 | 14 | 14 |
|  | SD | 2 | 2 | 2 | 0 | 2 | 2 | 2 | 2 | 14 |  |
| **Loubet *et al.* (2016)^152^** | EK | 2 | 2 | 2 | 0 | 2 | 2 | -1 | 2 | 11 | 11 |
|  | SD | 2 | 2 | 2 | 0 | 2 | 2 | -1 | 2 | 11 |  |
| **MacDougall *et al.* (2016)^153^** | EK | 2 | 2 | 2 | 0 | -2 | -2 | 2 | 2 | 6 | 6 |
|  | SD | 2 | 2 | 2 | 0 | -2 | -2 | 2 | 2 | 6 |  |
| **Maher *et al.* (2013)^154^** | EK | 2 | 2 | 2 | 0 | 2 | 2 | -2 | 2 | 10 | 10 |
|  | SD | 2 | 2 | 2 | 0 | 2 | 2 | -2 | 2 | 10 |  |
| **Mak *et al.* (2015)^155^** | EK | 2 | 2 | 2 | 0 | 2 | 2 | 2 | 2 | 14 | 14 |
|  | SD | 2 | 2 | 2 | 0 | 2 | 2 | 2 | 2 | 14 |  |
| **Mak *et al.* (2018)^156^** | EK | -1 | 2 | 2 | 0 | 2 | 2 | 2 | 2 | 11 | 11 |
|  | SD | -1 | 2 | 2 | 0 | 2 | 2 | 2 | 2 | 11 |  |
| **de Mattos *et al.* (2003)^157^** | EK | -1 | 2 | 2 | 0 | -2 | -2 | -2 | 2 | -1 | -1 |
|  | SD | -1 | 2 | 2 | 0 | -2 | -2 | -2 | 2 | -1 |  |
| **Maurici *et al.* (2016)^158^** | EK | 2 | 2 | 2 | 0 | -2 | -2 | -2 | -2 | -2 | -2 |
|  | SD | 2 | 2 | 2 | 0 | -2 | -2 | -2 | -2 | -2 |  |
| **Mayet *et al.* (2017)^159^** | EK | 2 | 2 | 2 | 0 | 2 | 2 | 2 | 2 | 14 | 14 |
|  | SD | 2 | 2 | 2 | 0 | 2 | 2 | 2 | 2 | 14 |  |
| **McCarthy *et al.* (2012)^160^** | EK | -2 | 2 | 2 | 0 | -2 | -2 | 2 | 2 | 2 | 2 |
|  | SD | -2 | 2 | 2 | 0 | -2 | -2 | 2 | 2 | 2 |  |
| **McCarthy *et al.* (2015)^161^** | EK | -1 | 2 | 2 | 0 | -2 | -2 | 2 | 2 | 3 | 3 |
|  | SD | -1 | 2 | 2 | 0 | -2 | -2 | 2 | 2 | 3 |  |
| **McQuaid *et al.* (2018)^162^** | EK | -2 | 2 | 2 | 0 | -2 | -2 | 2 | 2 | 2 | 6 |
|  | SD | 2 | 2 | 2 | 0 | -2 | -2 | 2 | 2 | 6 |  |
| **Mitra & Manna (1997)^163^** | EK | -2 | -2 | -2 | 0 | -2 | -2 | -2 | 2 | -10 | -10 |
|  | SD | -2 | -2 | -2 | 0 | -2 | -2 | -2 | 2 | -10 |  |
| **Mohammed *et al.* (2018)^164^** | EK | 2 | 2 | 2 | 0 | 2 | 2 | -2 | 2 | 10 | 10 |
|  | SD | 2 | 2 | 2 | 0 | 2 | 2 | -2 | 2 | 10 |  |
| **Napolitano *et al.* (2017)^165^** | EK | -1 | 2 | 2 | 0 | 2 | 2 | 2 | 2 | 11 | 14 (SR) |
|  | SD | 2 | 2 | 2 | 0 | 2 | 2 | 2 | 2 | 14 |  |
| **O'Grady *et al.* (2015)^166^** | EK | 2 | 2 | 2 | 0 | -2 | -2 | -2 | -2 | -2 | -2 |
|  | SD | 2 | 2 | 2 | 0 | -2 | -2 | -2 | -2 | -2 |  |
| **Og Son *et al.* (2014)^167^** | EK | -1 | -2 | 2 | 0 | -2 | -2 | 0 | 2 | -3 | -4 |
|  | SD | -1 | -2 | 2 | 0 | -2 | -2 | -1 | 2 | -4 |  |
| **Ozer *et al.* (2010)^168^** | EK | -2 | -2 | 2 | 0 | 2 | 2 | -2 | 2 | 2 | 6 |
|  | SD | -2 | 2 | 2 | 0 | 2 | 2 | -2 | 2 | 6 |  |
| **Ozkaya *et al.* (2011)^169^** | EK | -2 | -2 | -1 | 0 | -2 | -2 | -2 | 2 | -9 | -8 |
|  | SD | -2 | -1 | -1 | 0 | -2 | -2 | -2 | 2 | -8 |  |
| **Puchalski *et al.* (2014)^170^** | EK | 2 | 2 | 2 | 0 | -2 | -2 | 2 | 2 | 6 | 6 |
|  | SD | 2 | 2 | 2 | 0 | -2 | -2 | 2 | 2 | 6 |  |
| **Regan *et al.* (2016)^171^** | EK | -2 | 2 | 2 | 0 | -2 | 2 | 2 | 2 | 6 | 10 |
|  | SD | -2 | 2 | 2 | 0 | 2 | 2 | 2 | 2 | 10 |  |
| **Sakaguchi *et al.* (2011)^172^** | EK | 2 | 2 | 2 | 0 | -2 | -2 | -2 | 2 | 2 | 2 |
|  | SD | 2 | 2 | 2 | 0 | -2 | -2 | -2 | 2 | 2 |  |
| **Siddiqui *et al.* (2017)^173^** | EK | 2 | 2 | 2 | 0 | -2 | -2 | 2 | 2 | 6 | 6 |
|  | SD | 2 | 2 | 2 | 0 | -2 | -2 | 2 | 2 | 6 |  |
| **Silverman & Greif (2001)^174^** | EK | -2 | 2 | 2 | 0 | -2 | -2 | -2 | 2 | -2 | -2 |
|  | SD | -2 | 2 | 2 | 0 | -2 | -2 | -2 | 2 | -2 |  |
| **Song *et al.* (2017)^175^** | EK | -2 | -2 | 2 | 0 | -2 | -2 | -2 | 2 | -6 | -6 |
|  | SD | -1 | -2 | 2 | 0 | -2 | -2 | -2 | 2 | -5 |  |
| **Stark *et al.* (2016)^176^** | EK | -2 | 2 | 2 | 0 | -2 | 2 | 2 | 2 | 6 | 10 (SR) |
|  | SD | -2 | 2 | 2 | 0 | -1 | 2 | 2 | 2 | 7 |  |
| **Steelfisher *et al.* (2011)^177^** | EK | 2 | 2 | 2 | 0 | -1 | -1 | 2 | 2 | 8 | 5 |
|  | SD | -1 | 2 | 2 | 0 | -1 | -1 | 2 | 2 | 5 |  |
| **Strassberg *et al.* (2018)^178^** | EK | -1 | 2 | 2 | 0 | -2 | -2 | -1 | 2 | 0 | 0 |
|  | SD | -1 | 2 | 2 | 0 | -2 | -2 | -1 | 2 | 0 |  |
| **Taksdal *et al.* (2013)^179^** | EK | 2 | -2 | 2 | 0 | 2 | 2 | 2 | 2 | 10 | 10 |
|  | SD | 2 | -2 | 2 | 0 | 2 | 2 | 2 | 2 | 10 |  |
| **Tarrant *et al.* (2013)^180^** | EK | 2 | 2 | 2 | 0 | -2 | 2 | -2 | 2 | 6 | 10 |
|  | SD | 2 | 2 | 2 | 0 | 2 | 2 | -2 | 2 | 10 |  |
| **Tong *et al.* (2008)^181^** | EK | 2 | 2 | 2 | 0 | 2 | 2 | -2 | 2 | 10 | 10 |
|  | SD | 2 | 2 | 2 | 0 | 2 | 2 | -2 | 2 | 10 |  |
| **Tuells *et al.* (2018)^182^** | EK | 2 | 2 | 2 | 0 | 2 | 2 | -2 | 2 | 10 | 10 |
|  | SD | 2 | 2 | 2 | 0 | 2 | 2 | -2 | 2 | 10 |  |
| **Van Lier *et al.* (2012)^183^** | EK | -2 | 2 | 2 | 0 | 2 | 2 | -2 | 2 | 6 | 6 |
|  | SD | -2 | 2 | 2 | 0 | 2 | 2 | -2 | 2 | 6 |  |
| **Varan *et al.* (2014)^184^** | EK | 2 | 2 | 2 | 0 | 2 | 2 | 2 | 2 | 14 | 14 |
|  | SD | 2 | 2 | 2 | 0 | 2 | 2 | 2 | 2 | 14 |  |
| **Vila-Candel *et al.* (2016)^185^** | EK | 2 | 2 | -1 | 0 | -2 | -2 | 2 | 2 | 3 | 3 |
|  | SD | 2 | 2 | -1 | 0 | -2 | -2 | 2 | 2 | 3 |  |
| **White *et al.* (2010)^186^** | EK | -2 | -2 | 2 | 0 | -2 | -2 | 2 | 2 | -2 | -5 |
|  | SD | -2 | -2 | 2 | 0 | -2 | -2 | -1 | 2 | -5 |  |
| **Wilcox *et al.* (2018)^187^** | EK | 2 | -2 | -2 | 0 | -2 | -2 | -2 | 2 | -6 | -6 |
|  | SD | 2 | -2 | -2 | 0 | -2 | -2 | -2 | 2 | -6 |  |
| **Wilcox *et al.* (2019)^188^** | EK | 2 | 2 | 2 | 0 | 2 | 2 | -2 | 2 | 10 | 7 |
|  | SD | -1 | 2 | 2 | 0 | 2 | 2 | -2 | 2 | 7 |  |
| **Wiley *et al.* (2013.a)^189^** | EK | -2 | 2 | 2 | 0 | -2 | 2 | -2 | 2 | 2 | 2 |
|  | SD | -2 | 2 | 2 | 0 | -2 | 2 | -1 | 2 | 3 |  |
| **Wiley *et al.* (2013.b)^190^** | EK | -2 | 2 | 2 | 0 | -2 | 2 | 2 | -2 | 2 | 2 |
|  | SD | -2 | 2 | 2 | 0 | -2 | 2 | 2 | -2 | 2 |  |
| **Ymba & Perry (2003)^191^** | EK | -2 | 2 | 2 | 0 | -2 | -2 | -2 | 2 | -2 | -2 |
|  | SD | -2 | 2 | 2 | 0 | -2 | -2 | -2 | 2 | -2 |  |
| **Yudin *et al.* (2009)^192^** | EK | -2 | 2 | 0 | 0 | -2 | -2 | 2 | 2 | 0 | 0 |
|  | SD | -2 | 2 | 0 | 0 | -2 | -2 | 2 | 2 | 0 |  |
| **Yuen *et al.* (2013)^193^** | EK | 2 | 2 | 2 | 0 | 2 | 2 | -2 | 2 | 10 | 10 |
|  | SD | 2 | 2 | 2 | 0 | 2 | 2 | -2 | 2 | 10 |  |
| **Yun & Xu (2010)^194^** | EK | -2 | -2 | -1 | 0 | -2 | -2 | 2 | 2 | -5 | -4 |
|  | SD | -2 | 2 | -1 | 0 | -2 | -2 | -2 | -2 | -9 |  |

**JBI Ratings – Qualitative Studies**

Key:

*Philosophy:* Is there congruity between the stated philosophical perspective and the research methodology?

*Objectives:* Is there congruity between the research methodology and the research question or objectives?

*Methods:* Is there congruity between the research methodology and the methods used to collect data?

*Analysis:* Is there congruity between the research methodology and the representation and analysis of data?

*Results:* Is there congruity between the research methodology and the interpretation of results?

*Cultural:* Is there a statement locating the researcher culturally or theoretically?

*Influence:* Is the influence of the researcher on the research, and vice- versa, addressed?

*Voice:* Are participants, and their voices, adequately represented?

*Ethics:* Is the research ethical according to current criteria or, for recent studies, and is there evidence of ethical approval by an appropriate body?

*Conclusion:* Do the conclusions drawn in the research report flow from the analysis, or interpretation, of the data?

| **Study ID** | **Reviewer** | **Philosophy** | **Objectives** | **Methods** | **Analysis** | **Results** | **Cultural** | **Influence** | **Voice** | **Ethics** | **Conclusions** | **Total** | **Agreed** |
| --- | --- | --- | --- | --- | --- | --- | --- | --- | --- | --- | --- | --- | --- |
| **Bettinger *et al.* (2016)^104^** | EK | 2 | 2 | 2 | 2 | 2 | -2 | -2 | 2 | 2 | 2 | 12 | 10 |
|  | SD | 2 | 2 | 2 | 2 | 2 | -2 | -2 | 2 | 2 | 2 | 12 |  |
| **Barroso Pereira *et al.* (2011)^195^** | EK | 2 | 2 | 2 | 2 | 2 | -2 | -2 | -1 | -1 | 2 | 10 | 10 |
|  | SD | 2 | 2 | 2 | 2 | 2 | -2 | -2 | -1 | -1 | 2 | 10 |  |
| **Cassady *et al.* (2012)^196^** | EK | 2 | 2 | 2 | 2 | 2 | 2 | -1 | -1 | 2 | 2 | 14 | 14 |
|  | SD | 2 | 2 | 2 | 2 | 2 | 2 | -1 | 2 | 2 | 2 | 17 |  |
| **Collins *et al.* (2014)^197^** | EK | 2 | 2 | 2 | 2 | 2 | 2 | -1 | 2 | 2 | 2 | 17 | 0 |
|  | SD | 2 | 2 | 2 | 2 | 2 | 2 | -1 | 2 | 2 | 2 | 17 |  |
| **Fleming *et al.* (2018)^126^** | EK | -1 | 2 | 2 | -2 | -1 | 2 | -1 | -1 | 2 | 2 | 4 | 3 |
|  | SD | -1 | 2 | 2 | -2 | -1 | 2 | -2 | -1 | 2 | 2 | 3 |  |
| **Gauld *et al.* (2016)^198^** | EK | 0 | 2 | 2 | 2 | 2 | 2 | -2 | -1 | 2 | -1 | 8 | 8 |
|  | SD | 0 | 2 | 2 | 2 | 2 | 2 | -2 | -1 | 2 | -1 | 8 |  |
| **Kharbanda *et al.* (2011)^199^** | EK | -1 | 2 | 2 | 2 | 2 | 2 | -2 | 2 | 2 | -1 | 10 | 10 |
|  | SD | -1 | 2 | 2 | 2 | 2 | 2 | -2 | 2 | 2 | -1 | 10 |  |
| **Larson Williams *et al.* (2018)^200^** | EK | -1 | 2 | 2 | 2 | 2 | -2 | -1 | 2 | 2 | 2 | 10 | 11 |
|  | SD | 2 | 2 | 2 | 2 | 2 | -2 | -1 | 2 | 2 | 2 | 13 |  |
| **Lohiniva *et al.* (2014)^201^** | EK | -1 | 2 | 2 | 2 | 2 | 0 | -2 | 2 | 2 | 2 | 11 | 11 |
|  | SD | -1 | 2 | 2 | 2 | 2 | 0 | -2 | 2 | 2 | 2 | 11 |  |
| **Lohm *et al.* (2014)^202^** | EK | 2 | 2 | 2 | 2 | 2 | -2 | -2 | 2 | 2 | 2 | 12 | 12 |
|  | SD | 2 | 2 | 2 | 2 | 2 | -2 | -2 | 2 | 2 | 2 | 12 |  |
| **Lynch *et al.* (2012)^203^** | EK | 2 | 2 | 2 | 2 | 2 | -2 | -2 | 2 | 2 | 2 | 12 | 12 |
|  | SD | 2 | 2 | 2 | 2 | 2 | -2 | -2 | 2 | 2 | 2 | 12 |  |
| **Maisa *et al.* (2018)^204^** | EK | 2 | 2 | 2 | 2 | 2 | -2 | 2 | 2 | 2 | 2 | 16 | 16 |
|  | SD | 2 | 2 | 2 | 2 | 2 | -2 | 2 | 2 | 2 | 2 | 16 |  |
| **Marsh *et al.* (2014)^205^** | EK | -1 | 2 | 2 | 2 | 2 | -2 | 2 | 2 | 2 | 2 | 13 | 13 |
|  | SD | -1 | 2 | 2 | 2 | 2 | -2 | 2 | 2 | 2 | 2 | 13 |  |
| **McQuaid *et al.* (2016)^206^** | EK | 0 | 2 | 2 | 2 | 2 | -2 | 2 | -1 | 2 | 2 | 11 | 11 |
|  | SD | 0 | 2 | 2 | 2 | 2 | -2 | 2 | -1 | 2 | 2 | 11 |  |
| **Meharry *et al.* (2013)^207^** | EK | -2 | 2 | 2 | 2 | 2 | -2 | 2 | 2 | 2 | 2 | 12 | 12 |
|  | SD | -2 | 2 | 2 | 2 | 2 | -2 | 2 | 2 | 2 | 2 | 12 |  |
| **O'Grady *et al.* (2015)^166^** | EK | 2 | 2 | 2 | 2 | 2 | 2 | 2 | 2 | 2 | 2 | 20 | 20 |
|  | SD | 2 | 2 | 2 | 2 | 2 | 2 | 2 | 2 | 2 | 2 | 20 |  |
| **O'Shea *et al.* (2018)^208^** | EK | 0 | 2 | 2 | 2 | 2 | 2 | 2 | 2 | 2 | 2 | 18 | 18 |
|  | SD | 0 | 2 | 2 | 2 | 2 | 2 | 2 | 2 | 2 | 2 | 18 |  |
| **Richun *et al.* (2018)^209^** | EK | -1 | 2 | 2 | 2 | 2 | 2 | 2 | 2 | 2 | 2 | 17 | 17 |
|  | SD | -1 | 2 | 2 | 2 | 2 | 2 | 2 | 2 | 2 | 2 | 17 |  |
| **Schindler *et al.* (2012)^210^** | EK | 0 | 2 | 2 | 2 | 2 | 2 | 2 | 2 | 2 | 2 | 18 | 18 |
|  | SD | 0 | 2 | 2 | 2 | 2 | 2 | 2 | 2 | 2 | 2 | 18 |  |
| **Wiley *et al.* (2015)^211^** | EK | 2 | 2 | 2 | 2 | 2 | 2 | 2 | 2 | 2 | 2 | 20 | 20 |
|  | SD | 2 | 2 | 2 | 2 | 2 | 2 | 2 | 2 | 2 | 2 | 20 |  |
| **Yuen *et al.* (2016)^212^** | EK | 2 | 2 | 2 | 2 | 2 | -2 | -2 | 2 | 2 | 2 | 12 | 12 |
|  | SD | 2 | 2 | 2 | 2 | 2 | -2 | -2 | 2 | 2 | 2 | 12 |  |

# **Appendix 9. Distribution of included studies by country and vaccine**


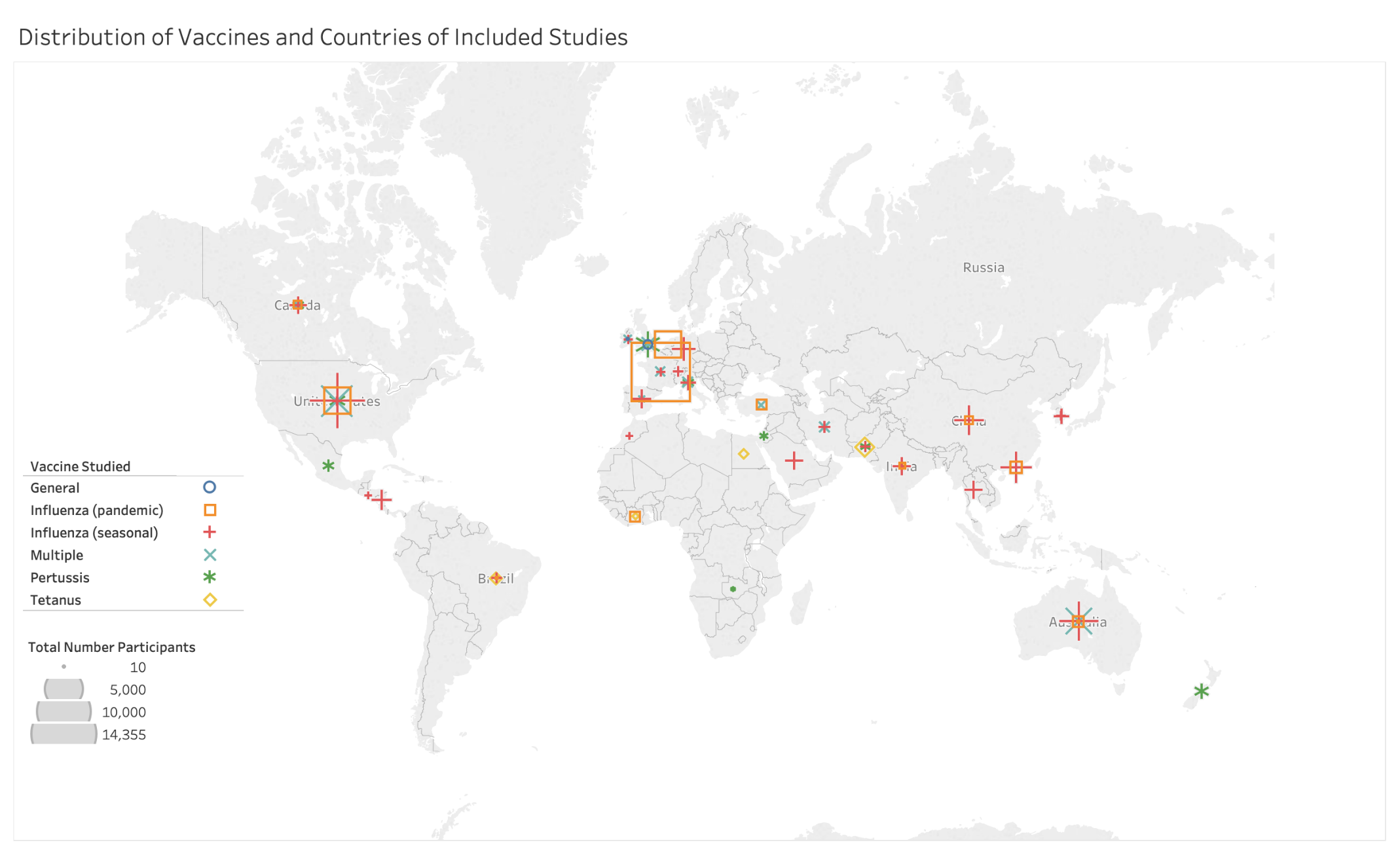


Figure 2 Map of study settings included in this systematic review & meta-analysis


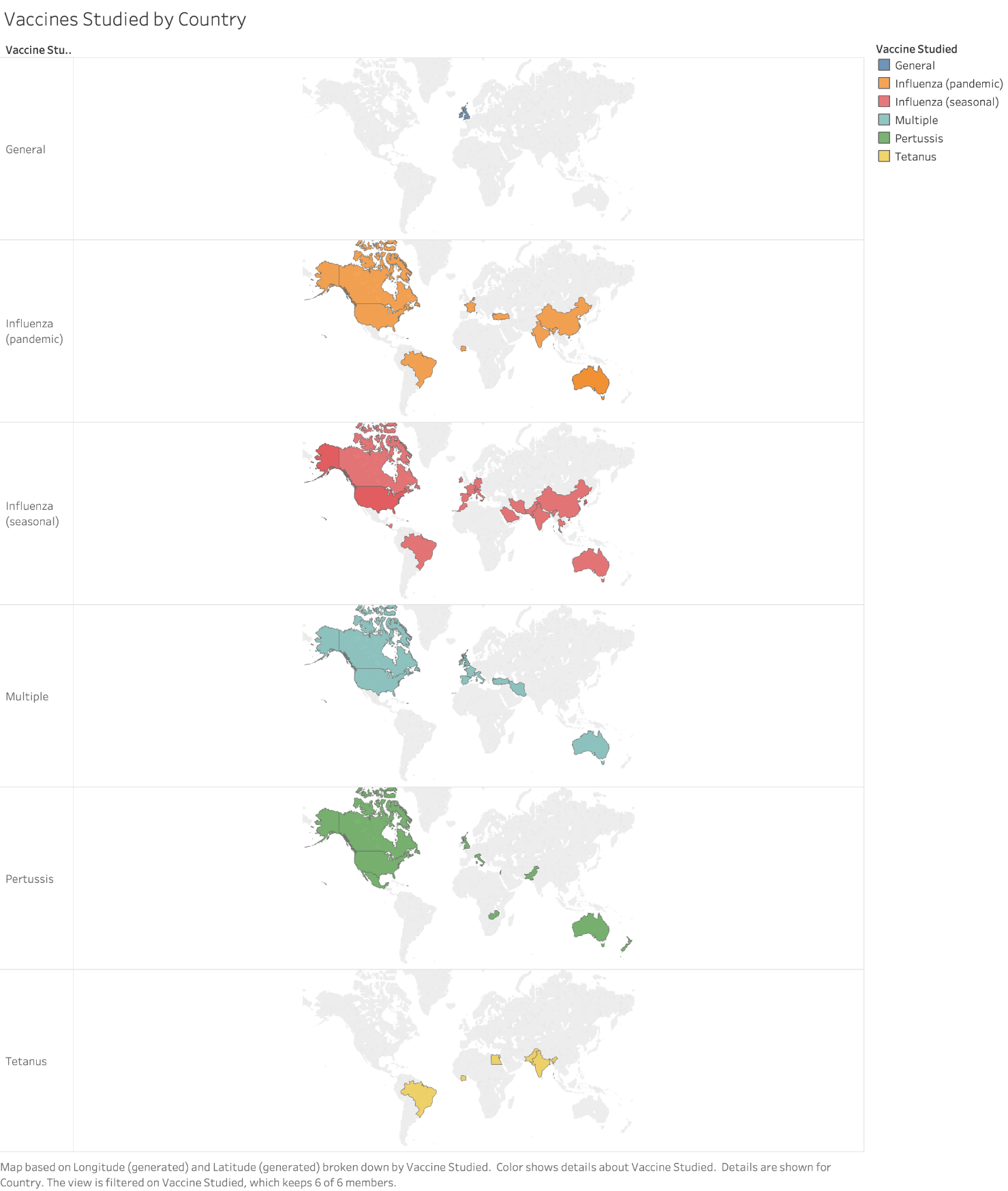


Figure 3 Maps demonstrating which vaccines were studied in each country


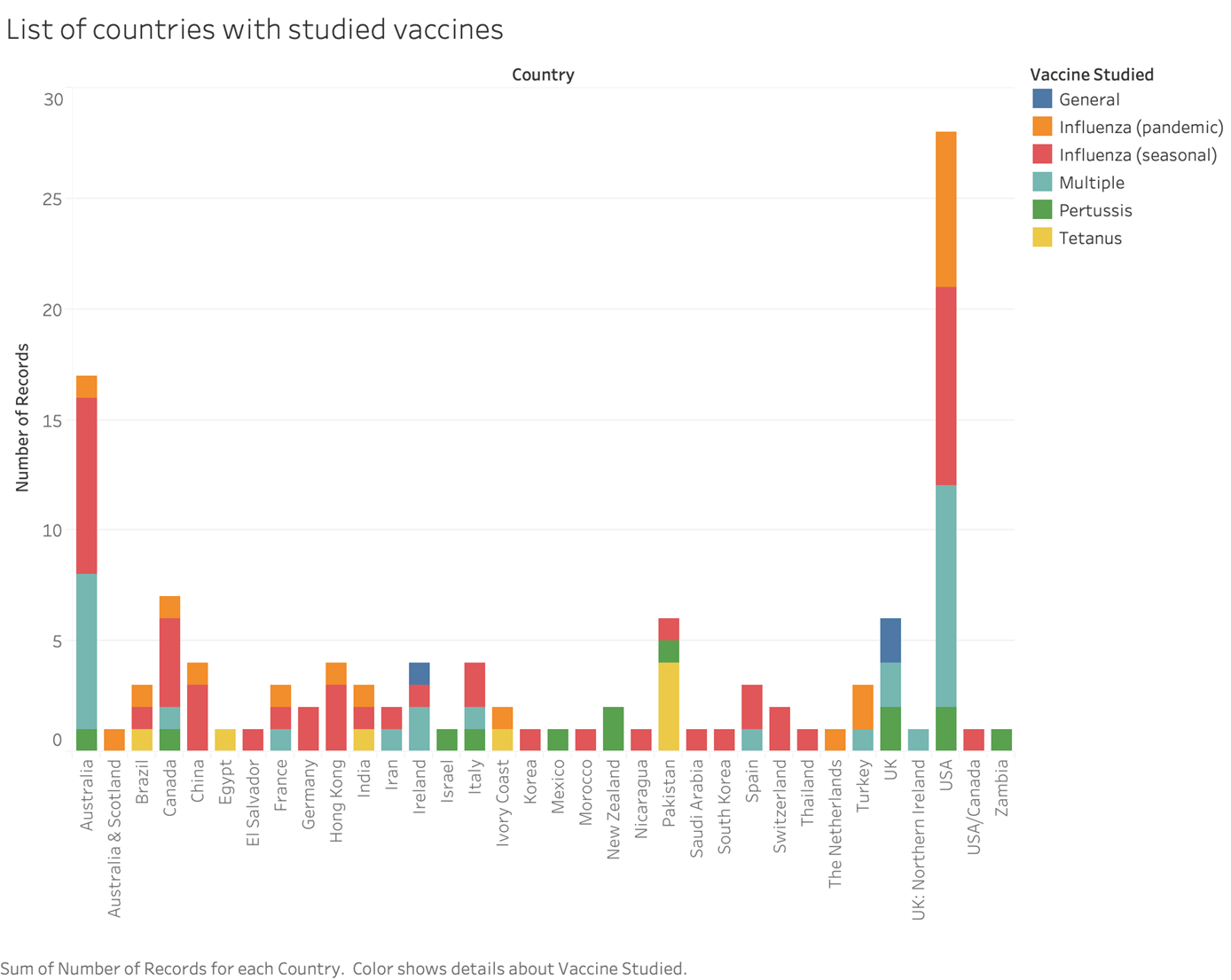


Figure 4 Chart depicting breakdown of studies for each vaccine per country


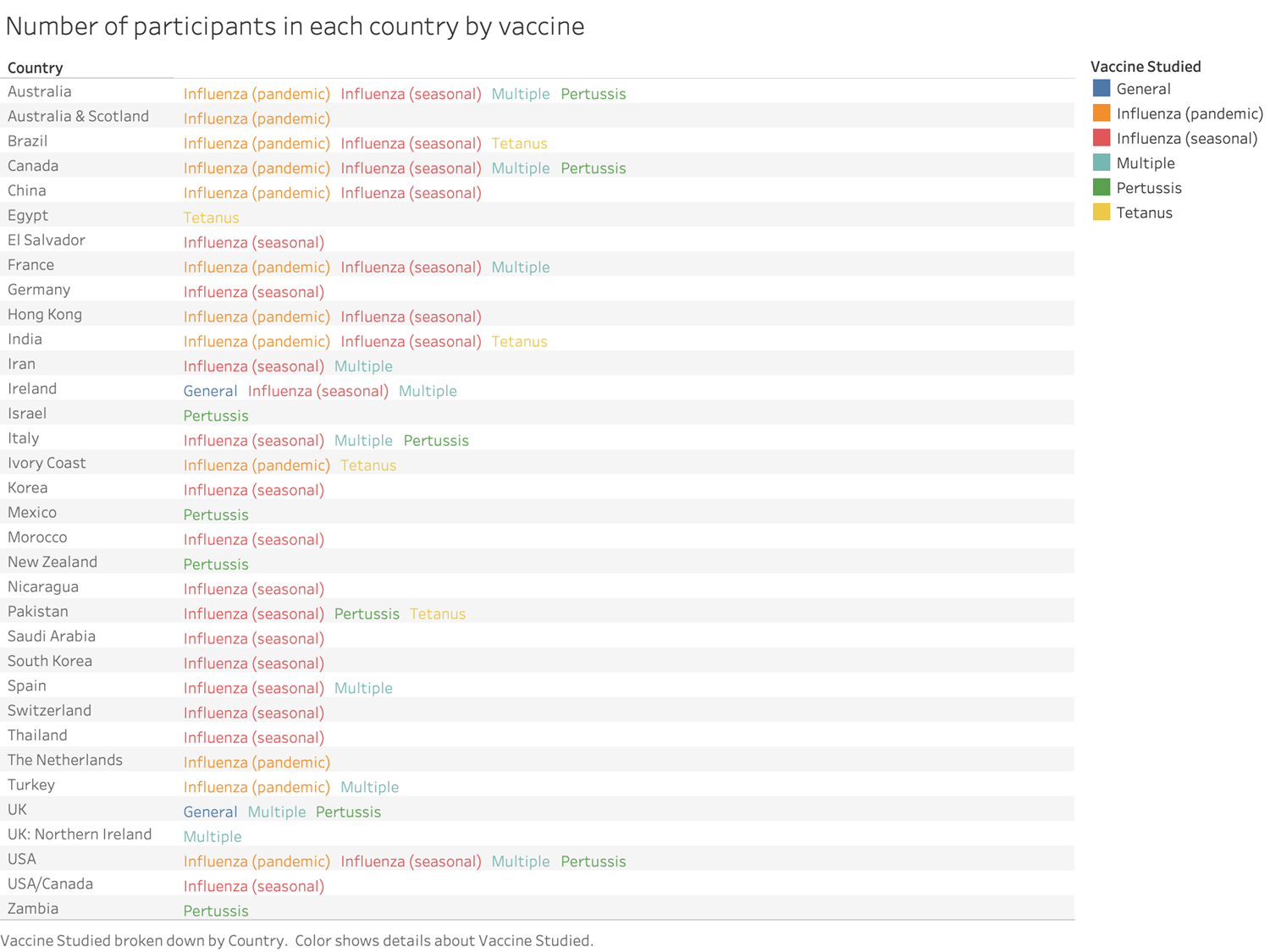


Figure 5 List of vaccines studied in each country


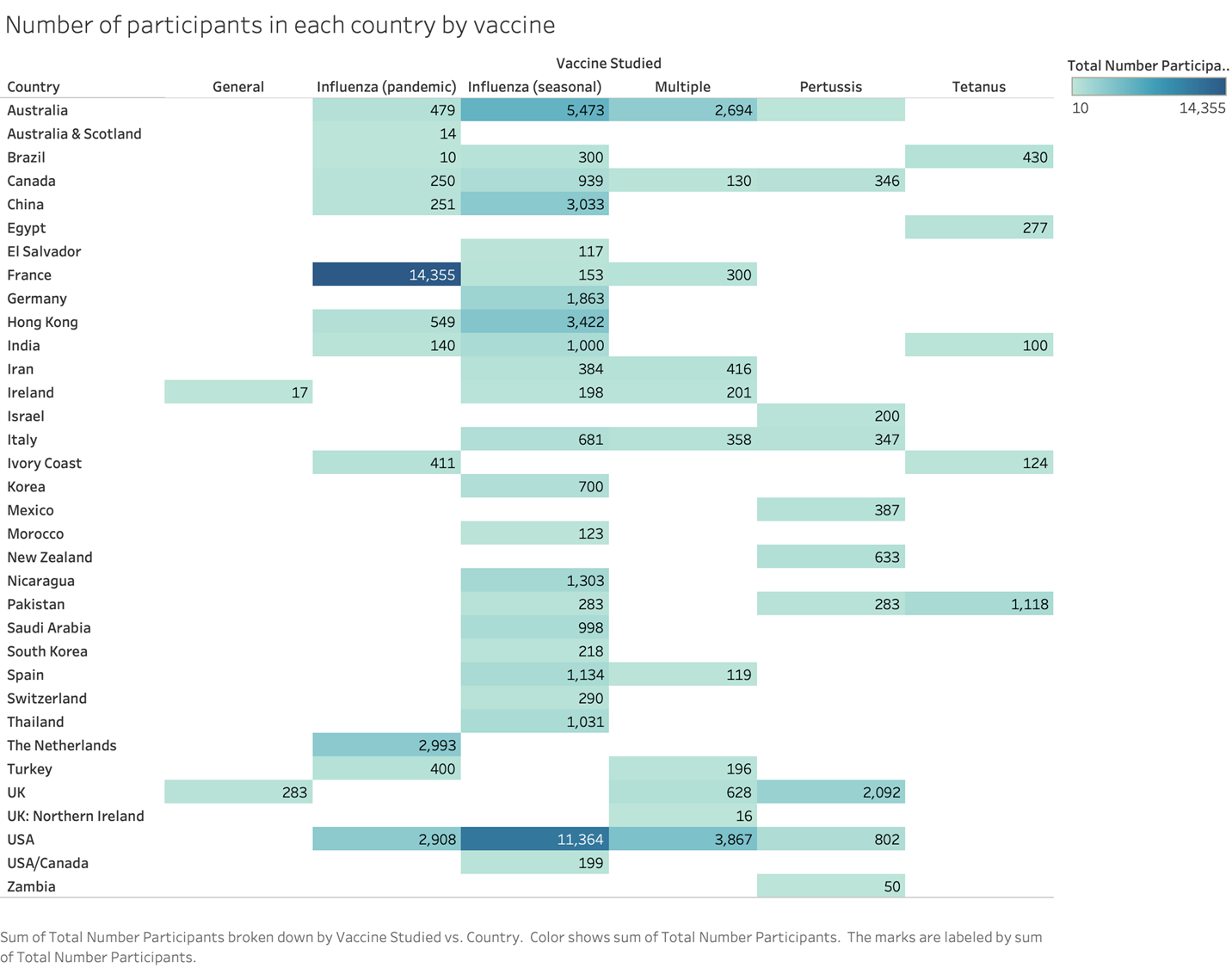


Figure 6 Chart demonstrating number of participants in each country for each type of vaccine

# **Appendix 10. Broad theme definitions.**

**Convenience**

- Statements regarding access, time, availability, cost and logistics of getting vaccinated
- Also includes statements of competing prioritization
- Examples:
  - “Vaccine was unavailable.”
  - “I don’t have time.”
  - “I did not know where/when to get the vaccine.”
  - “I could not afford it.”

**Influence of Personal History**

- Statements regarding the participants’ previous experiences with vaccinations, in pregnancy or in general
- Statements regarding chronic conditions or other health status influencing vaccination decisions
- Example:
  - “I received x vaccine in a previous pregnancy.”
  - “I always get the flu shot.”
  - “I had a bad experience with getting a vaccine.”
  - “I have a chronic illness.”

**Influence of Others**

- Statements regarding the influence of healthcare workers (GPs, midwives, nurses, obstetricians) or other people, family, friends and their recommendation to get the vaccine while pregnant.
- Also includes statements about receiving information from the media, Internet and other sources
- Examples:
  - “If a physician/nurse recommended it, I would get vaccinated.”
  - “Received a recommendation from an HCW.”
  - “My family discouraged vaccination.”
  - “I saw it [disease/vaccine information] on the news.”

**Informed**

- Statements referring to participants’ awareness or knowledge of the specific disease, vaccine or vaccines in pregnancy.
- Also includes knowledge of policy guidelines or awareness of general recommendations to be vaccinated
- Examples:
  - “I have heard of whooping cough.”
  - “I know about the influenza vaccine.”
  - “I am aware of the country’s vaccination policy.”

**Emotion**

- Statements referring to participants’ feelings or emotions regarding the vaccine
- Includes perceptions of rumours and myths
- Example:
  - “I am fearful/worried about the vaccine.”
  - “I don’t trust the vaccine.”
  - “I would regret not getting the vaccine.”

**Perception of Risk**

*Disease Harm*

- Statements referring the participants’ perception of disease harm.
- Includes perceived susceptibility to the disease as well as perceived severity of the disease (for the mother or infant)
- Examples:
  - “It is dangerous for a pregnant woman to contract influenza during pregnancy.”
  - “Influenza can be more dangerous for pregnant women.”
  - “Pregnant women are more susceptible to disease.”
  - “Worried about being sick, contagious or effect on pregnancy.”

*Vaccine Harm or Side Effect*

- Statements referring to the participants’ perception of the vaccine having side effects, causing harm or being unsafe.
- Examples:
  - “I am concerned about the side effects of the influenza vaccine.”
  - “I am concerned for my/the baby’s safety in regards to a vaccine.”
  - “Vaccines have not be adequately tested.”
  - “The vaccine is harmful.”
  - “The vaccine could cause x, y, z (negative consequences).”

**Perception of Benefit**

- Statements referring to the participants’ perception of a vaccine being useful, effective or necessary.
- Includes participants’ perceptions of the benefits of antenatal vaccination.
- Examples:
  - “The vaccine could protect me/the baby.”
  - “The vaccine is effective/ useful.”

# **Appendix 11. Coding process.**

*In analysing the data, we chose not to use a pre-existing framework such as the SAGE Working Group Framework for Vaccine Hesitancy. In developing codes based on the included studies, we aimed to capture the factors specifically relevant for pregnant and recently pregnant women. The following diagram describes how these codes were developed after extracting all the data into Excel.*

*3 studies were mixed methods (counted in quantitative and qualitative)

# **Appendix 12. Meta-analysis sub-category definitions.**

Each meta-analysis has a single exposure with a dichotomous answer (yes/no vs agree/disagree) to the following exposures and outcome (vaccinated/unvaccinated).

| **Factor Sub-categories (23 meta-analyses)** |
| --- |
| ***Informed*** |
| Aware of policy recommendation for vaccination during pregnancy |
| Felt informed to decide on vaccination during pregnancy |
| Aware of general information of vaccination for pregnant women |
| ***Disease severity*** |
| Disease is harmful to pregnancy |
| Disease may result in hospitalization |
| Disease may result in death |
| General perception of disease severity (severe vs mild) |
| ***Disease susceptibility*** |
| Pregnant women at increased risk for disease |
| Contagiousness of the disease |
| Baby at risk for disease |
| ***Vaccine side-effects*** |
| Knowledge of specific vaccine side-effects |
| Concern or perceived danger of vaccine side-effects |
| Perceived probability of vaccine side-effects |
| ***Vaccine harm in the state of pregnancy*** |
| Birth defects |
| Miscarriage or spontaneous abortion |
| Pre-term birth |
| General harm |
| ***Benefit of vaccines*** |
| Vaccine effectively protects/benefits pregnant women |
| Vaccine effectively protects/benefits foetus |
| Vaccine effectively protects/benefits the baby |
| General protection/benefit |
| ***Influence of healthcare professional recommendation*** |
| Healthcare professional recommendation |
| ***Personal history of vaccination*** |
| Vaccine during any previous pregnancy |
| Vaccine anytime outside of pregnancy in adulthood |

**Appendix 13. Pooled questions and responses for meta-analysis**

| **Meta-analyses definition** | **Pooled statements for meta-analysis** |
| --- | --- |
| Aware of policy recommendation | Informed of vaccine recommendations during pregnancy (yes/no) All pregnant women should get flu shot (yes/no) Awareness of national maternal vaccine recommendations (yes/no) It is recommended that all pregnant women get the flu vaccine (yes/no) |
| Felt informed to decide on vaccination during pregnancy | Have received all information to decide on vaccinating (yes/no) Had enough information to make decision about the vaccine (yes/no) |
| Aware of general information of vaccination for pregnant women | Received information on vaccines this pregnancy (yes/no) Had knowledge of vaccines (yes/no) (in pregnancy)  Heard of Tdap vaccine in pregnancy  Heard of Flu vaccine in pregnancy (yes/no) Saw information about vaccination when pregnant (yes/no) |
| Disease is Harmful to Pregnancy | Pregnant women have higher risk of flu complications (yes/no)  Infection with flu can adversely affect pregnancy (yes/no)  H1N1 could be harmful for my baby (agree/disagree) |
| Disease may result in Hospitalisation | Flu can be serious enough to require hospitalization (yes/no)  People with flu maybe admitted to the hospital (agree/disagree) |
| Disease may result in death | Seasonal flu can kill me (agree/disagree) |
| General perception of disease severity (severe vs mild) | H1N1 is serious infection (yes/no)  H1N1 can be harmful for me (agree/disagree)  H1N1 can be harmful for me (disagree/agree)  Perceived severity (high/low)  Not at risk of getting seriously ill from flu (Somewhat or strongly disagree/Strongly or somewhat agree or don't know)  Consequence of flu are more serious in pregnant women (yes/no)  Perceived severity of consequences of flu (mild/severe) |
| Pregnant women are at increased risk of the disease | I am at risk for the flu (agree/disagree)  Perceived susceptibility (low/ high)  I get sick with flu more easily than others my age (yes/no) |
| Contagiousness of the disease | Flu is contagious (True/False, do not know)  Seasonal flu is contagious (yes/no)  Prevalence of whooping cough in community (Common/Neither common nor rare or rare)  H1N1 spreads faster than seasonal flu (Strongly or somewhat agree/strongly or somewhat disagree or don't know)  Concerned about getting sick from H1N1 (Concerned/Not concerned) |
| Baby at risk of contracting disease*  **only present in intention to vaccinate dataset* | Moderately or very concerned about baby getting flu (Moderately or very concerned/not or little concerned)  Concerned about unborn baby getting flu (Very or moderate concern/not concerned)  Infants can contract pertussis (yes/no)  Newborns and infants more likely to contract pertussis (yes/no) |
| Knowledge of specific side effects | H1N1 vaccine can cause fever or other flu symptoms (yes/no)  H1N1 vaccine could lead to GBS (yes/ no) |
| Concern or perceived danger of vaccine side-effects | Perceived severity of vaccine side-effects (Not serious/very serious)  Flu shots have dangerous side-effects during pregnancy (yes/no)  I am concerned about side-effects of flu vaccine (yes/no)  I am concerned about side-effects of flu vaccine (agree/disagree)  A swine flu shot has unpleasant side effects for me (strongly agree, agree/ strongly disagree, disagree, neither agree or disagree) |
| Perceived probability of vaccine side-effects | Perceived probability of vaccine side-effects (low/high score) |
| Birth defects | The flu shot causes birth defects (agree/disagree)  H1N1 vaccine during pregnancy can cause birth defects (agree/disagree) |
| Miscarriage or spontaneous abortion | H1N1 vaccine can increase chance of miscarriage (agree/disagree)  H1N1 vaccine during pregnancy may induce spontaneous abortion (yes/no) |
| Pre-term birth | Flu vaccine can cause pre-term births (true/false) H1N1 vaccine during pregnancy may induce pre-term contractions (agree/disagree) |
| Vaccine General harm | Flu vaccine is dangerous in pregnancy (true/false)  H1N1 vaccine during pregnancy can hurt unborn baby (agree/disagree)  Flu shot is not safe for the baby (yes/no)  Having flu shot during pregnancy could harm my baby (yes/no)  I am concerned about safety of vaccine during pregnancy (yes/no)  Influenza vaccine is safe and does not pose risk to baby (Somewhat or strongly disagree/ Somewhat or strongly agree or neutral)  Concerned about safety of flu vaccine for baby (yes/no)  Vaccine could harm unborn baby (No or don’t know/yes)  Safety of H1N1 vaccine (Somewhat or not safe/very safe)  Believe flu vaccine is unsafe for infant (yes/no)  Baby might get flu in womb if I get H1N1 vaccine (agree/disagree)  Concerned about baby's safety if having flu vaccine during pregnancy (agree/disagree)  Vaccine unsafe/safe  I think swine flu vaccine is harmful for my baby |
| Vaccine effectively protects pregnant women | Perceived vaccine effectiveness for pregnant women (Very or somewhat effective/ not effective or don’t know) The H1N1 vaccine could protect me from the disease (agree/disagree)  Perceived effectiveness of flu vaccine in preventing flu for mother (Very or somewhat effective/ not effective or don’t know)  Belief that flu vaccine protects pregnant women from effects of flu (yes/no)  Vaccine protects mother against flu (yes/no or don’t know)  Believe flu vaccine helps pregnant women (yes/no) |
| Vaccine effectively protects foetus | Benefit of vaccine for fetus (Provides protection/Don't know or no protection the fetus)  Foetus may benefit from H1N1 vaccine while still in the womb (agree/disagree) |
| Vaccine effectively protects the baby | If pregnant women get the flu shot it protects the baby (agree/disagree) Perceived effectiveness of flu vaccine in preventing flu for baby (Very or somewhat effective/ not effective or don’t know)  Belief that flu vaccine protects baby from flu after birth (yes/no)  Vaccine protects newborns from flu (yes/no or don’t know)  Believing vaccine protects infant (yes/no)  Vaccine during pregnancy can have protective effect on baby for first 6 months of life (agree/disagree)  Belief that maternal vaccine is helpful for baby (yes/no)  Vaccine helps the baby (agree/disagree) |
| General protection/benefit | Perception of effectiveness of flu vaccine (Very or somewhat effective/ not effective or don’t know)  Perceived benefit of vaccine (low/high score)  Perceived effectiveness (low/ high score)  Aware of vaccine protection (yes/no)  Vaccine protects 100% against H1N1 flu (yes/no)  Believe vaccine is effective (yes/no)  Perceived benefits (low/high score) |
| Healthcare professional recommendation | Received recommendation for flu vaccine from HCP (yes/no)  HCP advised vaccine (yes/no)  Received vaccine advise from a physician (yes/no)  HCP recommended vaccine during pregnancy (yes/no)  Number of times doctor reminded about flu shot (Never/one or more times)  Provider recommendation (yes/no)  My primary care provider recommended a flu shot (Agree or strongly agree/disagree or strongly disagree or neutral)  HCP recommended dTpa vaccine (yes/no)  HCP recommended Flu vaccine (yes/no)  Recommendation from HCP (yes/no)  Clinician recommendation (yes/no)  Provider recommendation received (yes/no)  ANC provider advise (yes/no)  Recommendation from HCP (yes/no)  HCP recommendation during pregnancy (yes/no)  Provider recommendation received (yes/no)  Vaccine recommendation from HCP (yes/no)  HCP recommended vaccine during pregnancy (yes/no)  Advised by HCP to receive vaccine (yes/no)  HCP advised vaccine (yes/no) |
| Vaccine during any previous pregnancy | Previously vaccine during pregnancy (yes/no)  Vaccine in previous pregnancy (yes/no)  Vaccine in previous pregnancy (yes/no)  Vaccinated during prior pregnancy (yes/no) |
| Vaccine anytime outside of pregnancy in adulthood | Received flu vaccine before this pregnancy (yes/no)  Vaccine during previous flu season (yes/no)  Received seasonal flu vaccine in the past (yes/no)  Ever had the flu shot before (yes/no)  Received flu vaccine before this pregnancy (yes/no)  Previous dTpa (yes/no)  Previous flu vaccine (yes/no)  Received seasonal flu vaccine in the past (yes/no)  Received season flu vaccine before (yes/no)  Ever been vaccinated against flu (yes/no)  Vaccinated in the previous flu season (yes/no)  Received seasonal flu shot before (yes/no)  Received flu vaccine before this pregnancy (yes/no) |
| DTpa = Diphtheria Tetanus, acellular pertussis vaccine, flu = influenza | |

# **Appendix 14. Reasons for exclusion from meta-analysis**

| Study (99 Quant Only, 102 Quant + Mixed) | Informed | Disease Severity | Disease Susceptibility | Vaccine side-effects | Vaccine harm to pregnancy | Benefit of the Vaccine | Healthcare Professional Recommendation | Personal History of Vaccination |
| --- | --- | --- | --- | --- | --- | --- | --- | --- |
| Number of Studies That Assessed Exposure | **61** | **53** | **54** | **63** | **78** | **72** | **69** | **49** |
| Number of Studies Included in Meta-Analysis | **13** | **14** | **11** | **10** | **14** | **18** | **26** | **18** |
| Number of Studies Included in Intention Sensitivity | **4** | **7** | **7** | **2** | **8** | **7** | **4** | **7** |
| Abasi *et al.* 2015^97^ | Reasons vaccination declined/accepted | Reasons vaccination declined/accepted | Reasons vaccination declined/accepted | Reasons vaccination declined/accepted | Reasons vaccination declined/accepted | Reasons vaccination declined/accepted | Exposure not assessed | Exposure not assessed |
| Agricola *et al.* 2016^98^ | Exposure not assessed | Exposure not assessed | Reasons vaccination declined/accepted | Reasons vaccination declined/accepted | Reasons vaccination declined/accepted | Reasons vaccination declined/accepted | Reasons vaccination declined/accepted | Intention to vaccinate |
| Arriola *et al.* 2018^99^ | Insufficient data | Exposure not assessed | Insufficient data | Exposure not assessed | Insufficient data | Included in Meta-analysis | Included in Meta-analysis | Included in Meta-analysis |
| Ashfaq *et al.* 2017^100^ | Did not assess exposure with outcome | Exposure not assessed | Exposure not assessed | Exposure not assessed | Exposure not assessed | Exposure not assessed | Exposure not assessed | Did not assess exposure with outcome |
| Barrett *et al.* 2018^101^ | Included in Meta-analysis | Exposure not assessed | Reasons vaccination declined/accepted | Exposure not assessed | Reasons vaccination declined/accepted | Reasons vaccination declined/accepted | Included in Meta-analysis | Included in Meta-analysis |
| Beigi *et al.* 2009^102^ | Exposure not assessed | Did not assess exposure with outcome | Did not assess exposure with outcome | Did not assess exposure with outcome | Did not assess exposure with outcome | Exposure not assessed | Exposure not assessed | Exposure not assessed |
| Ben Natan *et al.* 2017^103^ | Exposure not assessed | Exposure not discrete | Exposure not discrete | Exposure not assessed | Intention to vaccinate | Exposure not discrete | Exposure not assessed | Exposure not assessed |
| Bettinger *et al.* 2016^104^ | Did not assess exposure with outcome | Exposure not assessed | Exposure not assessed | Exposure not assessed | Did not assess exposure with outcome | Did not assess exposure with outcome | Exposure not assessed | Exposure not assessed |
| Bhaskar *et al.* 2012^105^ | Exposure not assessed | Exposure not assessed | Exposure not assessed | Exposure not assessed | Exposure not assessed | Exposure not assessed | Did not assess exposure with outcome | Exposure not assessed |
| Blanchard-Rohner *et al.* 2012^106^ | Included in Meta-analysis | Included in Meta-analysis | Included in Meta-analysis | Did not assess exposure with outcome | Included in Meta-analysis | Reasons vaccination declined/accepted | Exposure not discrete | Included in Meta-analysis |
| Blondel *et al.* 2012^107^ | Exposure not assessed | Exposure not assessed | Exposure not assessed | Exposure not assessed | Exposure not assessed | Exposure not assessed | Exposure not assessed | Exposure not assessed |
| Boedeker *et al.* 2014^108^ | Reasons vaccination declined/accepted | Included in Meta-analysis | Exposure not assessed | Included in Meta-analysis | Exposure not assessed | Included in Meta-analysis | Included in Meta-analysis | Included in Meta-analysis |
| Boedeker *et al.* 2015^93^ | Included in Meta-analysis | Exposure not assessed | Exposure not discrete | Reasons vaccination declined/accepted | Reasons vaccination declined/accepted | Reasons vaccination declined/accepted | Reasons vaccination declined | Included in Meta-analysis |
| Campbell *et al.* 2015^109^ | Did not assess exposure with outcome | Did not assess exposure with outcome | Exposure not assessed | Did not assess exposure with outcome | Did not assess exposure with outcome | Intention to vaccinate | Did not assess exposure with outcome | Exposure not assessed |
| Castro-Sanchez *et al.* 2018^110^ | Exposure not discrete | Exposure not assessed | Reasons vaccination declined/accepted | Reasons vaccination declined/accepted | Exposure not assessed | Reasons vaccination declined/accepted | Reasons vaccination declined/accepted | Exposure not assessed |
| Celikel *et al.* 2014^111^ | Exposure not assessed | Reasons vaccination declined/accepted | Did not assess exposure with outcome | Reasons vaccination declined/accepted | Reasons vaccination declined/accepted | Exposure not assessed | Reasons vaccination declined/accepted | Exposure not assessed |
| Chamberlain *et al.* 2015^112^ | Exposure not assessed | Intention to vaccinate | Exposure not assessed | Exposure not assessed | Intention to vaccinate | Exposure not assessed | Exposure not assessed | Exposure not discrete |
| Chamberlain *et al.* 2016^113^ | Exposure not assessed | Exposure not assessed | Exposure not assessed | Reasons vaccination declined/accepted | Reasons vaccinated declined/accepted | Exposure not assessed | Exposure not assessed | Reasons vaccination declined/accepted |
| D'Alessandro *et al.* 2018^114^ | Exposure not assessed | Intention to vaccinate | Exposure not assessed | Exposure not discrete | Exposure not discrete | Exposure not assessed | Exposure not assessed | Exposure not assessed |
| Dempsey *et al.* 2016^115^ | Intention to vaccinate | Intention to vaccinate | Intention to vaccinate | Intention to vaccinate | Intention to vaccinate | Intention to vaccinate | Exposure not assessed | Intention to vaccinate |
| Ding *et al.* 2011^116^ | Did not assess exposure with outcome | Exposure not assessed | Did not assess exposure with outcome | Did not assess exposure with outcome | Exposure not assessed | Did not assess exposure with outcome | Did not assess exposure with outcome | Exposure not assessed |
| Ditsungnoen *et al.* 2016^117^ | Exposure not assessed | Intention to vaccinate | Intention to vaccinate | Exposure not assessed | Intention to vaccinate | Intention to vaccinate | Intention to vaccinate | Intention to vaccinate |
| Dlugacz *et al.* 2012^118^ | Exposure not assessed | Included in Meta-analysis | Exposure not assessed | Included in Meta-analysis | Included in Meta-analysis | Exposure not assessed | Included in Meta-analysis | Included in Meta-analysis |
| Donaldson *et al.* 2015^119^ | Reasons vaccination declined/accepted | Reasons vaccination declined/accepted | Reasons vaccination declined/accepted | Reasons vaccination declined/accepted | Reasons vaccination declined/accepted | Exposure not assessed | Reasons vaccination declined/accepted | Exposure not assessed |
| Drees *et al.* 2012^120^ | Exposure not assessed | Reasons vaccination declined/accepted | Reasons vaccination declined/accepted | Reasons vaccination declined/accepted | Reasons vaccination declined/accepted | Reasons vaccination declined/accepted | Included in Meta-analysis | Included in Meta-analysis |
| Drees *et al.* 2013^121^ | Exposure not assessed | Reasons vaccination declined/accepted | Reasons vaccination declined/accepted | Reasons vaccination declined/accepted | Reasons vaccination declined/accepted | Reasons vaccination declined/accepted | Insufficient Data | Insufficient data |
| Edmonds *et al.* 2011^122^ | Exposure not assessed | Intention to vaccinate | Intention to vaccinate | Exposure not assessed | Intention to vaccinate | Exposure not assessed | Exposure not assessed | Intention to vaccinate |
| Eppes *et al.* 2013^123^ | Included in Meta-analysis | Included in Meta-analysis | Included in Meta-analysis | Exposure not assessed | Included in Meta-analysis | Included in Meta-analysis | Exposure not assessed | Included in Meta-analysis |
| Fabry *et al.* 2011^124^ | Did not assess exposure with outcome | Included in Meta-analysis | Included in Meta-analysis | Included in Meta-analysis | Included in Meta-analysis | Included in Meta-analysis | Exposure not assessed | Exposure not assessed |
| Fisher *et al.* 2011^125^ | Reasons vaccination declined/accepted | Exposure not assessed | Exposure not assessed | Reasons vaccination declined/accepted | Reasons vaccination declined/accepted | Reasons vaccination declined/accepted | Exposure not assessed | Exposure not assessed |
| Fleming *et al.* 2018^126^ | Exposure not assessed | Exposure not assessed | Exposure not assessed | Exposure not assessed | Did not assess exposure with outcome | Exposure not assessed | Did not assess exposure with outcome | Did not assess exposure with outcome |
| Fridman *et al.* 2011^127^ | Exposure not assessed | Included in Meta-analysis | Included in Meta-analysis | Included in Meta-analysis | Included in Meta-analysis | Included in Meta-analysis | Exposure not assessed | Included in Meta-analysis |
| Gaudelus *et al.* 2016^128^ | Did not assess exposure with outcome | Exposure not assessed | Exposure not assessed | Reasons vaccination declined/accepted | Reasons vaccination declined/accepted | Reasons vaccination declined/accepted | Reasons vaccination declined/accepted | Did not assess exposure with outcome |
| Goldfarb *et al.* 2011^129^ | Exposure not assessed | Exposure not assessed | Exposure not assessed | Reasons vaccination declined/accepted | Reasons vaccination declined/accepted | Reasons vaccination declined/accepted | Reasons vaccination declined/accepted | Exposure not assessed |
| Gorman *et al.* 2012^130^ | Exposure not assessed | Included in Meta-analysis | Included in Meta-analysis | Included in Meta-analysis | Included in Meta-analysis | Included in Meta-analysis | Included in Meta-analysis | Included in Meta-analysis |
| Gul *et al.* 2016^131^ | Reasons vaccination declined/accepted | Exposure not assessed | Exposure not assessed | Exposure not assessed | Exposure not assessed | Did not assess exposure with outcome | Exposure not assessed | Exposure not assessed |
| Hallissey *et al.* 2018^132^ | Did not assess exposure with outcome | Did not assess exposure with outcome | Exposure not assessed | Exposure not assessed | Exposure not assessed | Exposure not assessed | Exposure not assessed | Exposure not assessed |
| Halperin *et al.* 2014^133^ | Did not assess exposure with outcome | Did not assess exposure with outcome | Did not assess exposure with outcome | Exposure not assessed | Did not assess exposure with outcome | Exposure not assessed | Reasons vaccination accepted/declined | Did not assess exposure with outcome |
| Hasnain & Sheikh 2007^134^ | Reasons vaccination declined/accepted | Exposure not assessed | Exposure not assessed | Reasons vaccination declined/accepted | Exposure not discrete | Reasons vaccination declined/accepted | Reasons vaccination declined | Exposure not assessed |
| Hassan *et al.* 2016^135^ | Insufficient data | Exposure not assessed | Exposure not assessed | Exposure not assessed | Exposure not assessed | Reasons vaccination declined/accepted | Reasons vaccination declined | Exposure not assessed |
| Hayles *et al.* 2015^136^ | Exposure not assessed | Exposure not assessed | Included in Meta-analysis | Exposure not assessed | Included in Meta-analysis | Exposure not assessed | Reasons vaccination declined/accepted | Reasons vaccination declined/accepted |
| Healy *et al.* 2015^137^ | Did not assess exposure with outcome | Exposure not assessed | Exposure not assessed | Reasons vaccination declined/accepted | Reasons vaccination declined/accepted | Reasons vaccination declined/accepted | Did not assess exposure with outcome | Did not assess exposure with outcome |
| Henninger *et al.* 2013^94^ | Included in Meta-analysis | Included in Meta-analysis | Included in Meta-analysis | Included in Meta-analysis | Exposure not assessed | Exposure not assessed | Included in Meta-analysis | Exposure not assessed |
| Henninger *et al.* 2015^95^ | Included in Meta-analysis | Included in Meta-analysis | Included in Meta-analysis | Included in Meta-analysis | Included in Meta-analysis | Included in Meta-analysis | Included in Meta-analysis | Exposure not assessed |
| Hill *et al.* 2018^138^ | Reasons vaccination declined/accepted | Exposure not assessed | Reasons vaccination declined/accepted | Reasons vaccination declined/accepted | Reasons vaccination declined/accepted | Reasons vaccination declined/accepted | Reasons vaccination declined/accepted | Exposure not assessed |
| Honarvar *et al.* 2012^139^ | Exposure not discrete | Exposure not assessed | Reasons vaccination declined/accepted | Reasons vaccination declined/accepted | Reasons vaccination declined/accepted | Reasons vaccination declined/accepted | Exposure not assessed | Exposure not assessed |
| Hu *et al.* 2017^140^ | Exposure not assessed | Intention to vaccinate | Intention to vaccinate | Exposure not assessed | Intention to vaccinate | Intention to vaccinate | Intention to vaccinate | Exposure not assessed |
| Jadoon *et al.* 2016^141^ | Exposure not assessed | Exposure not assessed | Exposure not assessed | Exposure not assessed | Exposure not assessed | Reasons vaccination declined/accepted | Exposure not assessed | Exposure not assessed |
| Kang *et al.* 2015^142^ | Reasons vaccination declined/accepted | Reasons vaccination declined/accepted | Reasons vaccination declined/accepted | Reasons vaccination declined/accepted | Reasons vaccination declined/accepted | Reasons vaccination declined/accepted | Reasons vaccination declined/accepted | Reasons vaccination declined |
| Kay *et al.* 2012^143^ | Exposure not assessed | Included in Meta-analysis | Reasons vaccination decline/accepted | Reasons vaccination declined/accepted | Reasons vaccination declined/accepted | Exposure not assessed | Included into Meta-analysis | Reasons vaccination declined |
| Kfouri & Richtmann 2013^144^ | Exposure not assessed | Exposure not assessed | Exposure not assessed | Exposure not assessed | Exposure not assessed | Included in Meta-analysis | Reasons vaccination declined | Exposure not assessed |
| Khan *et al.* 2015^145^ | Intention to vaccinate | Reasons vaccination declined/accepted | Exposure not assessed | Intention to vaccinate | Exposure not assessed | Intention to vaccinate | Intention to vaccinate | Intention to vaccinate |
| Kouassi *et al.* 2012^146^ | Did not assess exposure with outcome | Exposure not discrete | Intention to vaccinate | Exposure not assessed | Exposure not assessed | Exposure not assessed | Exposure not assessed | Exposure not assessed |
| Koul *et al.* 2014^147^ | Did not assess exposure with outcome | Exposure not assessed | Exposure not assessed | Exposure not assessed | Exposure not assessed | Exposure not assessed | Reasons vaccination declined | Exposure not assessed |
| Krishnaswamy *et al.* 2018^148^ | Included in Meta-analysis | Exposure not assessed | Reasons vaccination declined/accepted | Reasons vaccination declined/accepted | Reasons vaccination declined/accepted | Reasons vaccination declined/accepted | Included in Meta-analysis | Included in Meta-analysis |
| Kriss *et al.* 2018^149^ | Reasons vaccination declined/accepted | Reasons vaccination declined/accepted | Exposure not assessed | Reasons vaccination declined/accepted | Reasons vaccination declined/accepted | Exposure not assessed | Reasons vaccination declined/accepted | Exposure not assessed |
| Lau *et al.* 2010^150^ | Exposure not assessed | Did not assess exposure with outcome | Did not assess exposure with outcome | Insufficient data | Reasons vaccination declined/accepted | Insufficient data | Included in Meta-analysis | Exposure not assessed |
| Lotter *et al.* 2018^151^ | Exposure not assessed | Exposure not assessed | Reasons vaccination declined/accepted | Reasons vaccination declined/accepted | Reasons vaccination declined/accepted | Reasons vaccination declined/accepted | Included in Meta-analysis | Reasons vaccination declined/accepted |
| Loubet *et al.* 2016^152^ | Exposure not assessed | Exposure not assessed | Exposure not assessed | Exposure not assessed | Exposure not assessed | Reasons vaccination declined/accepted | Included in Meta-analysis | Did not assess exposure with outcome |
| MacDougall *et al.* 2016^153^ | Did not assess exposure with outcome | Did not assess exposure with outcome | Did not assess exposure with outcome | Did not assess exposure with outcome | Did not assess exposure with outcome | Exposure not assessed | Reasons vaccination declined/accepted | Exposure not assessed |
| Maher *et al.* 2013^154^ | Included in Meta-analysis | Included in Meta-analysis | Reasons vaccination declined/accepted | Exposure not assessed | Included in Meta-analysis | Included in Meta-analysis | Included in Meta-analysis | Exposure not assessed |
| Mak *et al.* 2015^155^ | Exposure not assessed | Exposure not assessed | Reasons vaccination declined/accepted | Reasons vaccination declined/accepted | Reasons vaccination declined/accepted | Reasons vaccination declined/accepted | Included in Meta-analysis | Reasons vaccination declined/accepted |
| Mak *et al.* 2018^156^ | Exposure not assessed | Exposure not assessed | Exposure not asssessed | Reasons vaccination declined/accepted | Reasons vaccination declined/accepted | Reasons vaccination declined/accepted | Included in Meta-analysis | Reasons vaccination declined/accepted |
| de Mattos *et al.* 2003^157^ | Exposure not assessed | Exposure not assessed | Exposure not assessed | Exposure not assessed | Exposure not assessed | Exposure not assessed | Exposure not assessed | Exposure not assessed |
| Maurici *et al.* 2016^158^ | Did not assess exposure with outcome | Did not assess exposure with outcome | Exposure not assessed | Did not assess exposure with outcome | Did not assess exposure with outcome | Did not assess exposure with outcome | Included in Meta-analysis | Did not assess exposure with outcome |
| Mayet *et al.* 2017^159^ | Did not assess exposure with outcome | Did not assess exposure with outcome | Did not assess exposure with outcome | Did not assess exposure with outcome | Did not assess exposure with outcome | Did not assess exposure with outcome | Did not assess exposure with outcome | Exposure not assessed |
| McCarthy *et al.* 2012^160^ | Reasons vaccination declined/accepted | Exposure not assessed | Reasons vaccination declined/accepted | Reasons vaccination declined/accepted | Reasons vaccination declined/accepted | Reasons vaccination declined/accepted | Reasons vaccination declined/accepted | Reasons vaccination declined/accepted |
| McCarthy *et al.* 2015^161^ | Reasons vaccination declined/accepted | Exposure not assessed | Reasons vaccination declined/accepted | Reasons vaccination declined/accepted | Reasons vaccination declined/accepted | Did not assess exposure with outcome | Reasons vaccination declined/accepted | Reasons vaccination declined/accepted |
| McQuaid *et al.* 2018^162^ | Did not assess exposure with outcome | Did not assess exposure with outcome | Did not assess exposure with outcome | Did not assess exposure with outcome | Did not assess exposure with outcome | Did not assess exposure with outcome | Exposure not assessed | Exposure not assessed |
| Mitra & Manna 1997^163^ | Exposure not assessed | Exposure not assessed | Exposure not assessed | Exposure not assessed | Exposure not assessed | Exposure not assessed | Exposure not assessed | Exposure not assessed |
| Mohammed *et al.* 2018^164^ | Included in Meta-analysis | Exposure not assessed | Reasons vaccination declined/accepted | Exposure not assessed | Exposure not assessed | Reasons vaccination declined/accepted | Included in Meta-analysis | Exposure not assessed |
| Napolitano *et al.* 2017^165^ | Did not assess exposure with outcome | Intention to vaccinate | Exposure not assessed | Exposure not assessed | Reasons vaccination declined/accepted | Intention to vaccinate | Reasons vaccination declined/accepted | Exposure not assessed |
| O’Grady *et al.* 2015^166^ | Exposure not assessed | Did not assess exposure with outcome | Exposure not assessed | Included in Meta-analysis | Included in Meta-analysis | Included in Meta-analysis | Exposure not assessed | Exposure not assessed |
| Og Son *et al.* 2014^167^ | Exposure not assessed | Reasons vaccination declined/accepted | Reasons vaccination declined/accepted | Reasons vaccination declined/accepted | Reasons vaccination declined/accepted | Reasons vaccination declined/accepted | Exposure not assessed | Included in Meta-analysis |
| Ozer *et al.* 2010^168^ | Exposure not discrete | Exposure not discrete | Included in Meta-analysis | Exposure not assessed | Exposure not assessed | Included in Meta-analysis | Exposure not assessed | Exposure not assessed |
| Ozkaya Parlakay *et al.* 2012^169^ | Exposure not assessed | Exposure not assessed | Exposure not assessed | Exposure not assessed | Exposure not assessed | Exposure not assessed | Exposure not assessed | Did not assess exposure with outcome |
| Puchalski *et al.* 2014^170^ | Exposure not assessed | Did not assess exposure with outcome | Did not assess exposure with outcome | Reasons vaccination declined/accepted | Reasons vaccination declined/accepted | Did not assess exposure with outcome | Exposure not assessed | Exposure not assessed |
| Regan *et al.* 2016^171^ | Exposure not assessed | Reasons vaccination declined/accepted | Exposure not assessed | Reasons vaccination declined/accepted | Reasons vaccination declined/accepted | Reasons vaccination declined/accepted | Reasons vaccination declined/accepted | Reasons vaccination declined/accepted |
| Sakaguchi *et al.* 2011^172^ | Exposure not assessed | Reasons vaccination declined/accepted | Exposure not assessed | Reasons vaccination declined/accepted | Reasons vaccination declined/accepted | Reasons vaccination declined/accepted | Reasons vaccination declined/accepted | Exposure not assessed |
| Siddiqui *et al.* 2017^173^ | Intention to vaccinate | Exposure not assessed | Intention to vaccinate | Exposure not assessed | Intention to vaccinate | Intention to vaccinate | Exposure not assessed | Intention to vaccinate |
| Silverman *et al.* 2001^174^ | Exposure not assessed | Did not assess exposure with outcome | Exposure not assessed | Exposure not assessed | Exposure not assessed | Included in Meta-analysis | Included in Meta-analysis | Included in Meta-analysis |
| Song *et al.* 2017^175^ | Exposure not assessed | Exposure not assessed | Exposure not assessed | Reasons vaccination declined/accepted | Reasons vaccination declined/accepted | Reasons vaccination declined/accepted | Included in Meta-analysis | Exposure not assessed |
| Stark *et al.* 2016^176^ | Exposure not assessed | Exposure not assessed | Exposure not assessed | Exposure not assessed | Did not assess exposure with outcome | Did not assess exposure with outcome | Did not assess exposure with outcome | Did not assess exposure with outcome |
| Steelfisher *et al.* 2011^177^ | Included in Meta-analysis | Included in Meta-analysis | Included in Meta-analysis | Exposure not assessed | Included in Meta-analysis | Included in Meta-analysis | Included in Meta-analysis | Included in Meta-analysis |
| Strassberg *et al.* 2018^178^ | Did not assess exposure with outcome | Exposure not assessed | Exposure not assessed | Reasons vaccination declined/accepted | Reasons vaccination declined/accepted | Reasons vaccination declined/accepted | Exposure not discrete | Exposure not discrete |
| Taksdal *et al.* 2013^179^ | Included in Meta-analysis | Exposure not asssessed | Exposure not asssessed | Exposure not assessed | Included in Meta-analysis | Included in Meta-analysis | Included in Meta-analysis | Exposure not assessed |
| Tarrant *et al.* 2013^180^ | Included in Meta-analysis | Included in Meta-analysis | Reasons vaccination declined/accepted | Included in Meta-analysis | Included in Meta-analysis | Included in Meta-analysis | Included in Meta-analysis | Included in Meta-analysis |
| Tong *et al.* 2008^181^ | Exposure not discrete | Exposure not assessed | Exposure not assessed | Included in Meta-analysis | Exposure not assessed | Included in Meta-analysis | Included in Meta-analysis | Included in Meta-analysis |
| Tuells *et al.* 2018^182^ | Reasons vaccination declined/accepted | Exposure not assessed | Exposure not assessed | Reasons vaccination declined/accepted | Reasons vaccination declined/accepted | Reasons vaccination declined/accepted | Did not assess exposure with outcome | Reasons vaccination declined/accepted |
| Ugezu *et al.* 2018^96^ | Did not assess exposure with outcome | Exposure not assessed | Exposure not assessed | Reasons vaccination declined/accepted | Reasons vaccination declined/accepted | Exposure not assessed | Exposure not assessed | Exposure not assessed |
| Van Lier *et al.* 2012^183^ | Insufficient data | Exposure not assessed | Insufficient data | Exposure not assessed | Insufficient data | Insufficient data | Exposure not discrete | Exposure not assessed |
| Varan *et al.* 2014^184^ | Intention to vaccinate | Reasons vaccination declined/accepted | Intention to vaccinate | Reason vaccination declined/accepted | Reason vaccination declined/accepted | Exposure not assessed | Intention to vaccinate | Intention to vaccinate |
| Vila-Candel *et al.* 2016^185^ | Reasons vaccinated declined/accepted | Exposure not assessed | Reasons vaccination declined/accepted | Reasons vaccination declined/accepted | Reasons vaccination declined/accepted | Reasons vaccination declined/accepted | Reasons vaccination declined/accepted | Included in Meta-analysis |
| White *et al.* 2010^186^ | Exposure not assessed | Exposure not assessed | Exposure not assessed | Exposure not assessed | Reasons vaccination declined/accepted | Exposure not assessed | Reasons vaccination declined/accepted | Exposure not assessed |
| Wilcox *et al.* 2018^187^ | Reasons vaccinated declined/accepted | Exposure not assessed | Exposure not assessed | Reasons vaccination declined/accepted | Exposure not assessed | Reasons vaccination declined/accepted | Exposure not assessed | Exposure not assessed |
| Wilcox *et al.* 2019^188^ | Exposure not assessed | Reason vaccination declined/accepted | Exposure not assessed | Exposure not assessed | Exposure not assessed | Reasons vaccination declined/accepted | Exposure not assessed | Exposure not assessed |
| Wiley *et al.* 2013^189^ | Did not assess exposure with outcome | Included in Meta-analysis | Exposure not assessed | Exposure not assessed | Included in Meta-analysis | Included in Meta-analysis | Included in Meta-analysis | Exposure not assessed |
| Wiley *et al.* 2013^190^ | Reasons vaccinated declined/accepted | Exposure not assessed | Exposure not assessed | Reasons vaccination declined/accepted | Reasons vaccination declined/accepted | Exposure not assessed | Exposure not discrete | Exposure not assessed |
| Ymba & Perrey 2003^191^ | Did not assess exposure with outcome | Exposure not assessed | Exposure not assessed | Exposure not assessed | Exposure not assessed | Exposure not assessed | Exposure not assessed | Exposure not assessed |
| Yudin *et al.* 2009^192^ | Did not assess exposure with outcome | Did not assess exposure with outcome | Did not assess exposure with outcome | Did not assess exposure with outcome | Did not assess exposure with outcome | Exposure not assessed | Exposure not assessed | Exposure not assessed |
| Yuen *et al.* 2013^193^ | Included in Meta-analysis | Reasons vaccinated declined/accepted | Included in Meta-analysis | Reasons vaccinate declined/accepted | Reasons vaccinate declined/accepted | Included in Meta-analysis | Included in Meta-analysis | Included in Meta-analysis |
| Yun & Xu 2010^194^ | Exposure not assessed | Exposure not assessed | Exposure not assessed | Reasons vaccinate declined/accepted | Reasons vaccinate declined/accepted | Exposure not assessed | Exposure not assessed | Exposure not assessed |

**Appendix 15. Authors contacted for further data where data was insufficient**

| Author | Study title | Exposures | Response | Data provided |
| --- | --- | --- | --- | --- |
| Arriola et al 2018^99^ | Knowledge, attitudes and practices about influenza vaccination among pregnant women and healthcare providers serving pregnant women in Managua, Nicaragua | Informed, disease susceptibility, vaccine harm to pregnancy | N | N |
| Chamberlain et al 2015^112^ | Factors Associated with Intention to Receive Influenza and Tetanus, Diphtheria, and Acellular Pertussis (Tdap) Vaccines during Pregnancy: A Focus on Vaccine Hesitancy and Perceptions of Disease Severity and Vaccine Safety | Vaccine harm to pregnancy (Influenza only) | Y | Y |
| Drees et al 2013^121^ | Sustained high inﬂuenza vaccination rates and decreased safety concerns among pregnant women during the 2010–2011 inﬂuenza season | Healthcare professional recommendation, personal history of vaccination | N | N |
| Fridman et al 2011^127^ | Predictors of H1N1 vaccination in pregnancy | Vaccine side-effects (p. Flu), vaccine harm to pregnancy (P.flu) | Y | Y |
| Kay et al 2012^143^ | 2009 Pandemic Inﬂuenza A Vaccination of Pregnant Women—King County, Washington State, 2009–2010 | Healthcare professional recommendation- data provided, disease severity- question not pooed | Y | Y |
| Lau et al 2010^150^ | Prevalence of influenza vaccination and associated factors among pregnant women in Hong Kong | Vaccine side-effects, benefit of the vaccine | Y | N |
| Van Lier et al 2012^183^ | Acceptance of vaccination during pregnancy: Experience with 2009 influenza A (H1N1) in the Netherlands | Informed, disease susceptibility, benefit of the vaccine, vaccine harm to pregnancy | N | N |
| Wiley et al 2013^189^ | Uptake of influenza vaccine by pregnant women: a cross-sectional survey. | Benefit of the vaccine | Y | Y |

# **Appendix 16. Most common facilitator/barrier cited in quantitative papers excluded from meta-analysis**

| **Study** | **Most common facilitator/barrier cited** | **Percentage of respondents who provided this factor as the main barrier or facilitator** |
| --- | --- | --- |
| Abasi *et al.* 2015^97^ | Vaccination risks for the fetus concern me | 34.90% |
| Ashfaq *et al.* 2017^100^ | Lack of knowledge | 50% |
| Beigi *et al.* 2009^102^ | Would accept vaccination during pregnancy if recommended by physician | 48.50% |
| Ben Natan *et al.* 2017^103^ | Correlations were found between all HBM constructs | . |
| Bettinger *et al.* 2016^104^ | . | . |
| Campbell *et al.* 2015^109^ | Safety of the vaccine for the baby and for themselves were cited as primary concerns. | 95.1% (baby), 81.8% (self) |
| Castro-Sanchez *et al.* 2018^110^ | They felt that the vaccine was unnecessary, | 25% |
| Celikel *et al.* 2014^111^ | Doctor did not recommend or Doctor did recommend | . |
| Chamberlain *et al.* 2016^113^ | Do not normally get an influenza vaccine | . |
| D'Alessandro *et al.* 2018^114^ | . | . |
| Ding *et al.* 2011^116^ | Received a doctor’s recommendation | 55.40% |
| Donaldson *et al.* 2015^119^ | Not aware and never informed about the vaccine | 51.30% |
| Drees *et al.* 2013^121^ | Provider recommended the inﬂuenza vaccination | . |
| Fisher *et al.* 2011^125^ | Not knowledgeable about the vaccine importance | 25% |
| Gaudelus *et al.* 2016^128^ | HCP advised for vaccination | 49% |
| Goldfarb *et al.* 2011^129^ | Desire to protect baby | 74-86% |
| Gul *et al.* 2016^131^ | Lack of Knowledge | 11.60% |
| Hallissey *et al.* 2018^132^ | . | . |
| Halperin *et al.* 2014^133^ | . | . |
| Hasnain & Sheikh 2007^134^ | Don’t know the importance of vaccine | . |
| Hassan *et al.* 2016^135^ | No recommendation from doctor | 42.60% |
| Healy *et al.* 2015^137^ | Would be immunized if recommended by their physician and safety the most important factor in decision making | 82.80% |
| Hill *et al.* 2018^138^ | Recommended by a health professional and to protect baby | . |
| Honarvar *et al.* 2012^139^ | More than one reason | 38.38 |
| Jadoon *et al.* 2016^141^ | Correlations were found between all HBM constructs | . |
| Kang *et al.* 2015^142^ | May harm the baby | 65% |
| Koul *et al.* 2014^147^ | it had not been recommended | 100% |
| Kriss *et al.* 2018^149^ | Provider recommendation, unsafe for mother or baby | 63% |
| Mayet *et al.* 2017^159^ | . | . |
| McCarthy *et al.* 2012^160^ | In 2010: I wanted to protect the baby (% of vaccinated women), I was worried about vaccine risk to the baby (% of unvaccinated women) | 67% (protect baby), 60% (vaccine risk) |
| McCarthy *et al.* 2015^161^ | In 2014: to protect the baby (% of vaccinated women), did not recall recommendation (% of unvaccinated women) | 89.2% (protect baby), 48.4% (no rec) |
| McQuaid *et al.* 2018^162^ | harm to the baby and speciﬁcally that it may cause a miscarriage | . |
| Mitra & Manna 1997^163^ | Vaccine not available | 71% |
| Puchalski *et al.* 2014^170^ | No answer | 26% |
| Regan *et al.* 2016^171^ | To protect the baby, Was not recommended by any HCP | . |
| Sakaguchi *et al.* 2011^172^ | concern about the risk of H1N1 infection in the fetus and/or themselves was the most cited reason for their decision | 73.10% |
| Song *et al.* 2017^175^ | Fear of harm to the fetus | 83% |
| Stark *et al.* 2016^176^ | . | . |
| Strassberg *et al.* 2018^178^ | HCW recommendation and educational material; Safety concern (e.g. Guillain-Barre, fetal effects, autism) and causal of subsequent illness | OR 8.38 (HCW rec), 16.5% (safety concern), 24.2% (vaccine illness) |
| Tuells *et al.* 2018^182^ | Unawareness | 29.5% |
| Ugezu *et al.* 2018^96^ | . | . |
| Van Lier *et al.* 2012^183^ | . | . |
| White *et al.* 2010^186^ | No HCP recommendation | 63.90% |
| Wilcox *et al.* 2018^187^ | Not included because duplicated population | . |
| Wilcox *et al.* 2019^188^ | Concern for side effects | 21% |
| Wiley *et al.* 2013^190^ | Safety concerns | 33% |
| Ymba & Perrey 2003^191^ | Agreement to be vaccinated was sometimes given unconditionally but often depended on the price of the vaccine | 31% |
| Yudin *et al.* 2009^192^ | . | . |
| Yun *et al.* 2010^194^ | Fear it would affect fetus | 60.6% |

# **Appendix 17. Funnel plot of HCP recommendation for seasonal influenza vaccination**


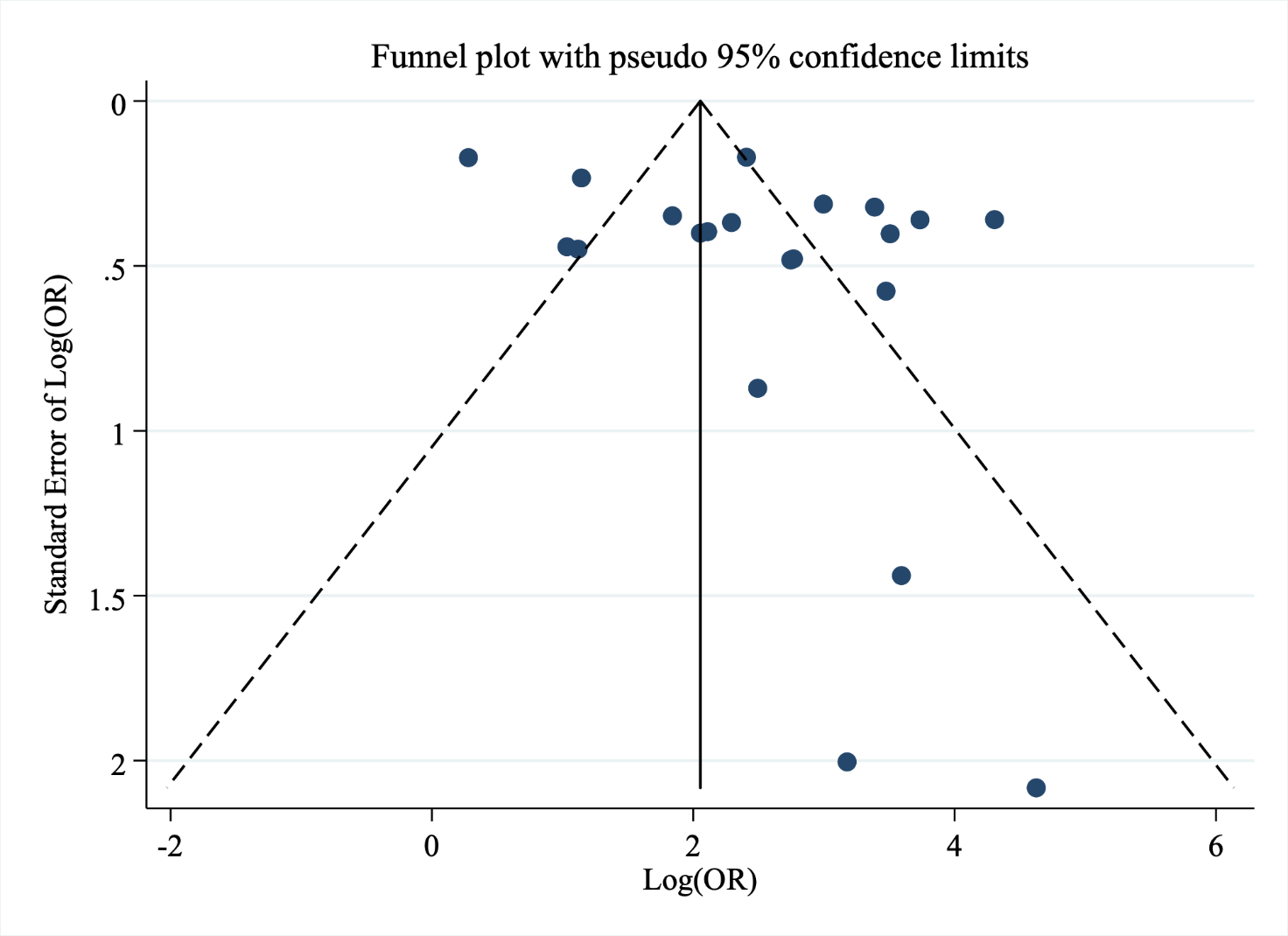


# **Appendix 18. Individual study forest plot for Pandemic Influenza and primary outcome of vaccination status**


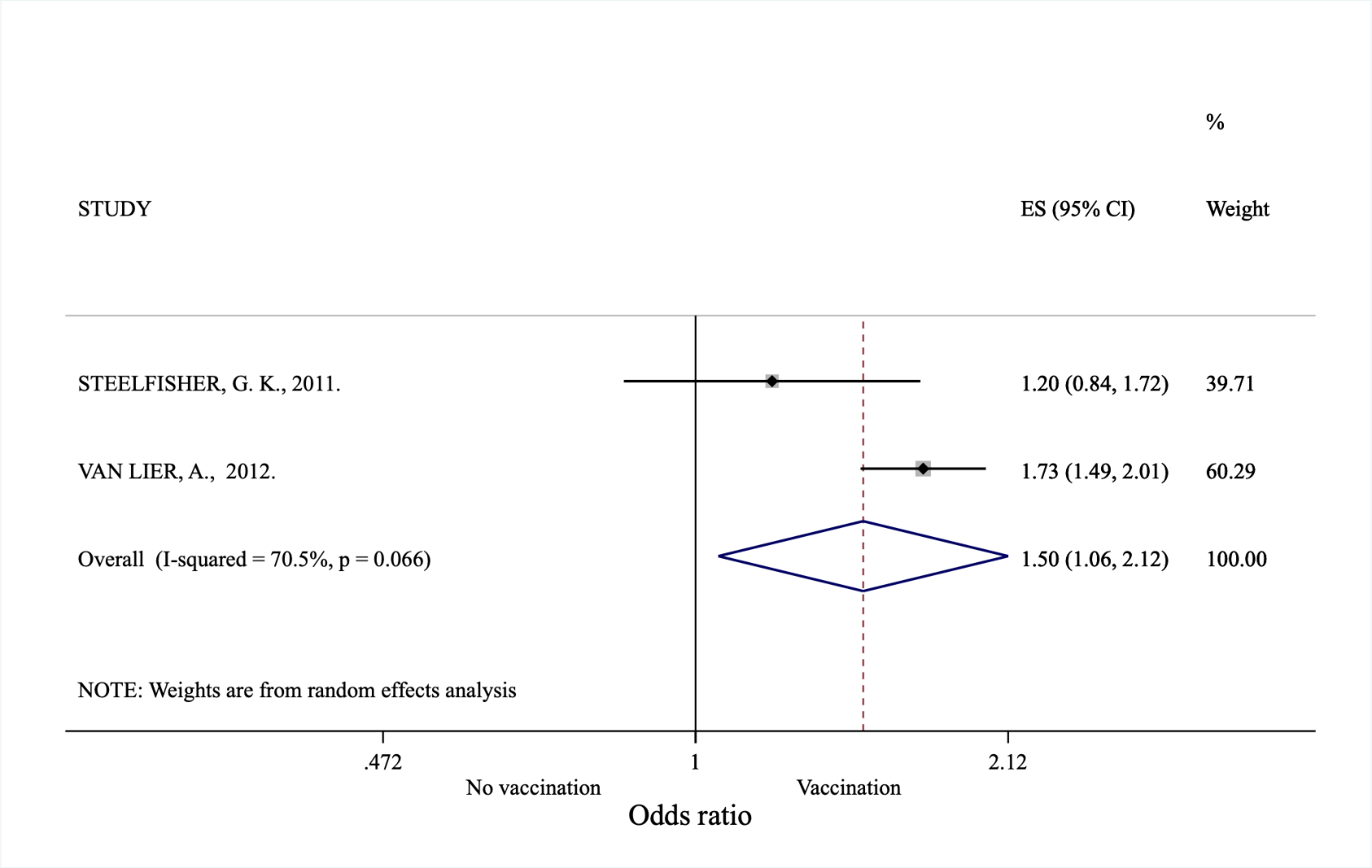


Figure 7 Forest plot for pandemic influenza vaccination and awareness of general information of vaccination for pregnant women (informed)


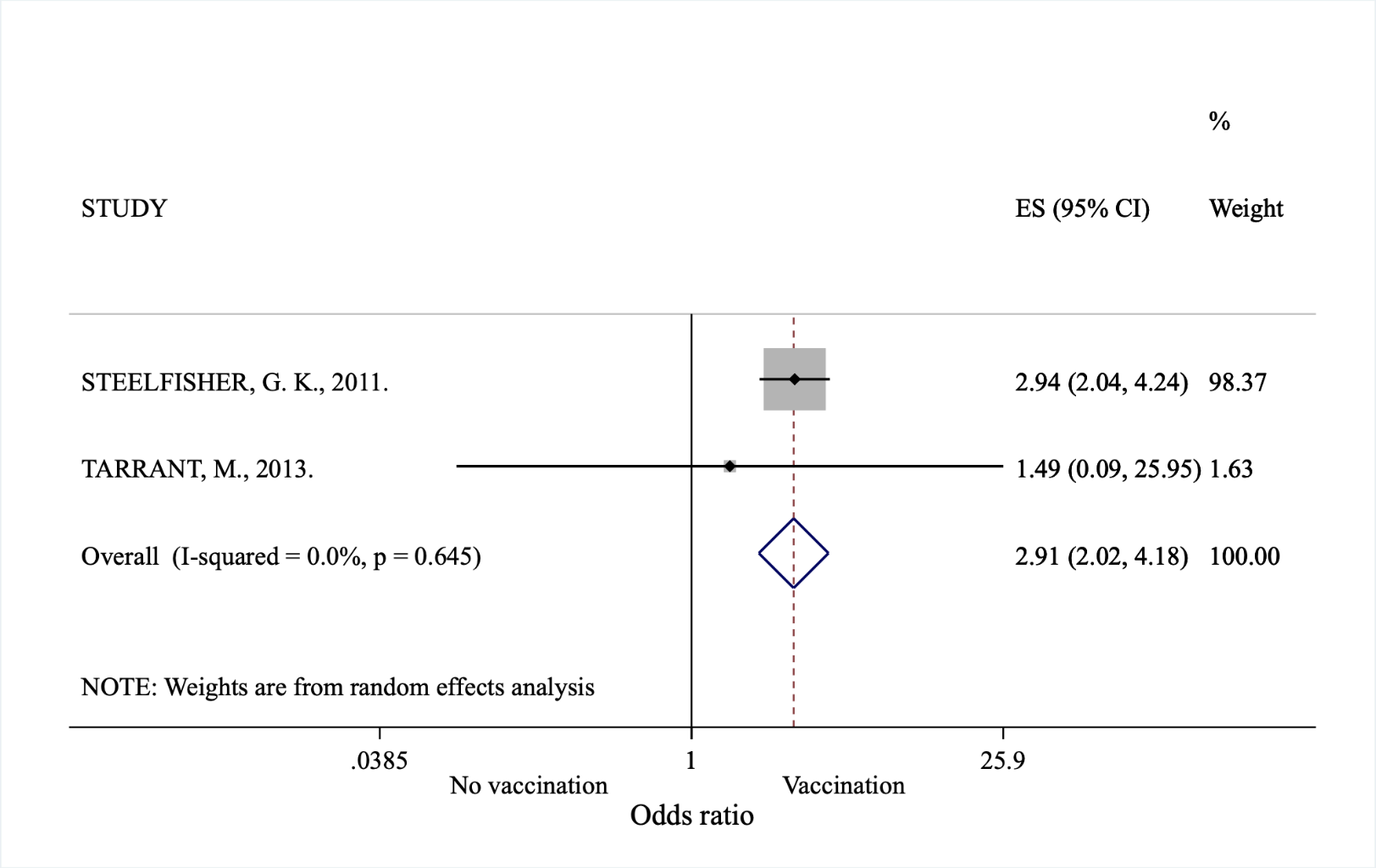


Figure 8 Forest plot for pandemic influenza vaccination and perception that disease may result in hospitalization (disease severity)


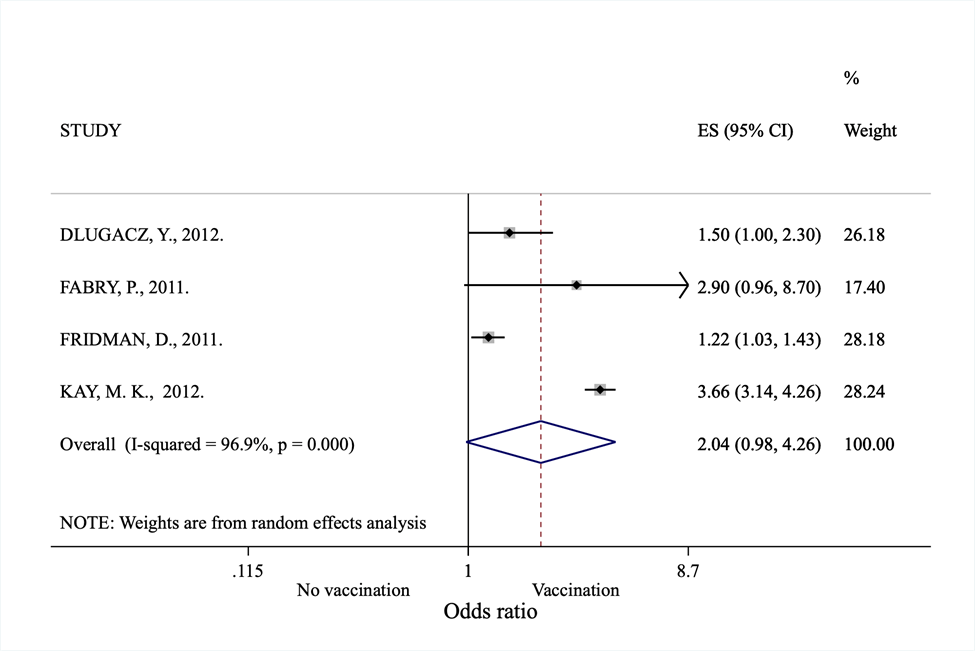


Figure 9 Forest plot for pandemic influenza vaccination and perception of general disease severity (disease severity)


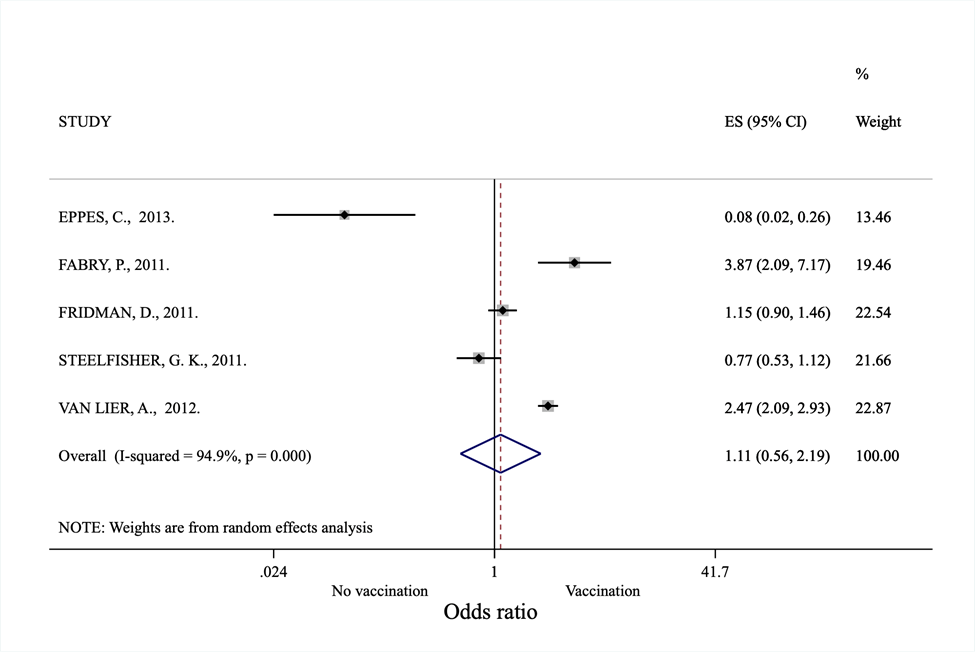


Figure 10 Forest plot for pandemic influenza vaccination and perception that pregnant women are at increased risk for disease (disease susceptibility)


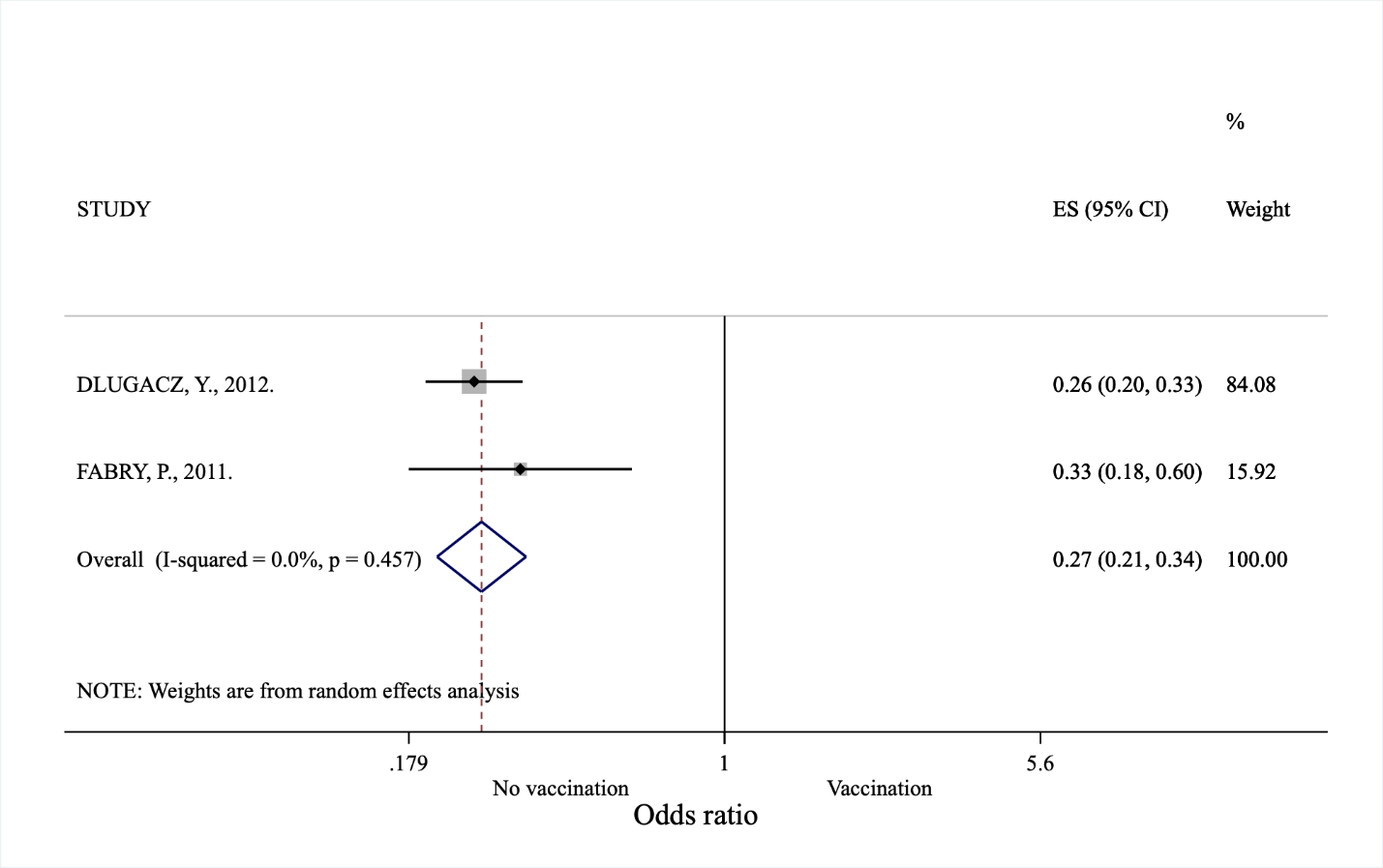


Figure 11 Forest plot for pandemic influenza vaccination and knowledge of specific vaccine side-effects (vaccine side-effects)


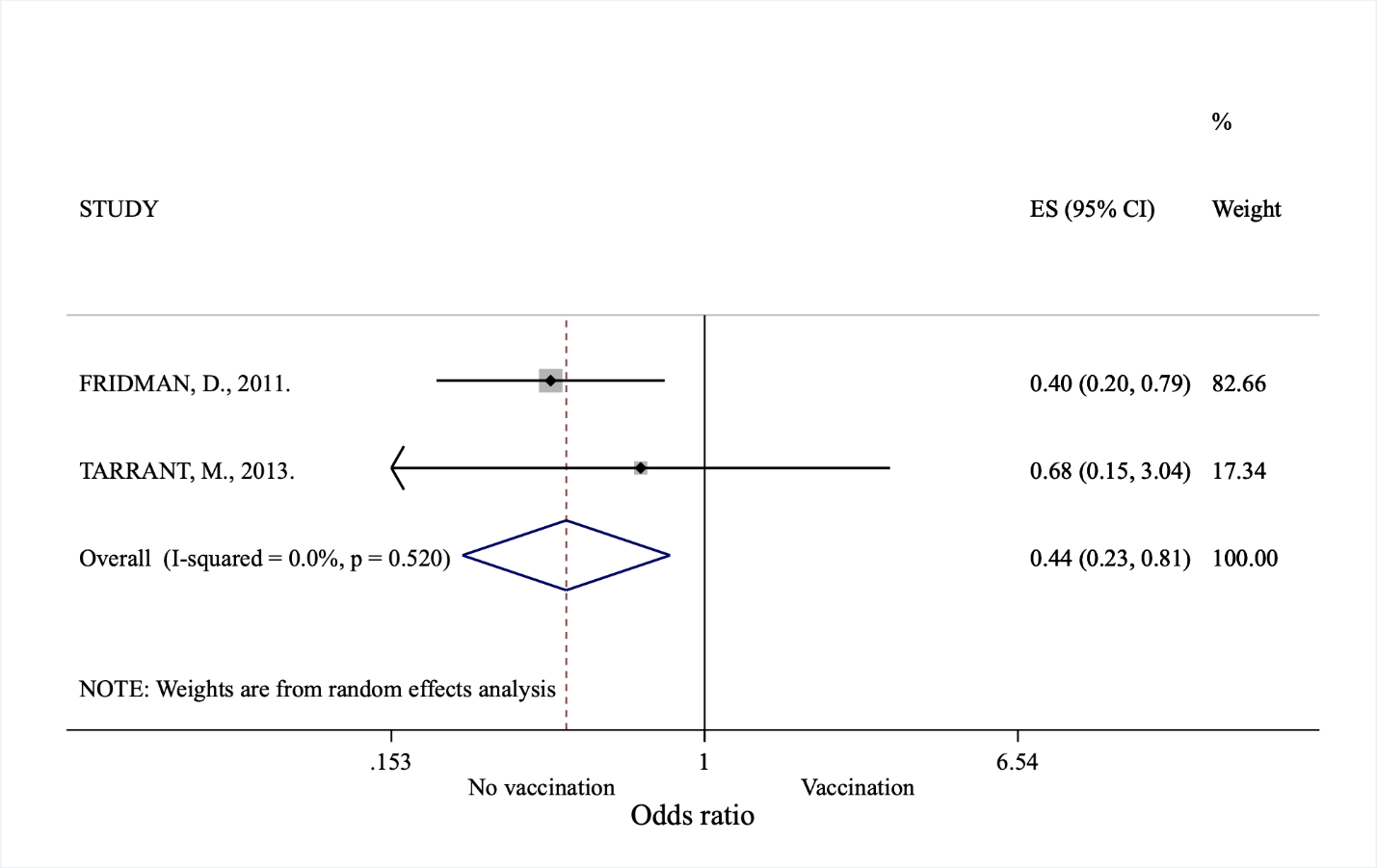


Figure 12 Forest plot for pandemic influenza vaccination and concern for or perceived danger of vaccine side-effects (vaccine side-effects)


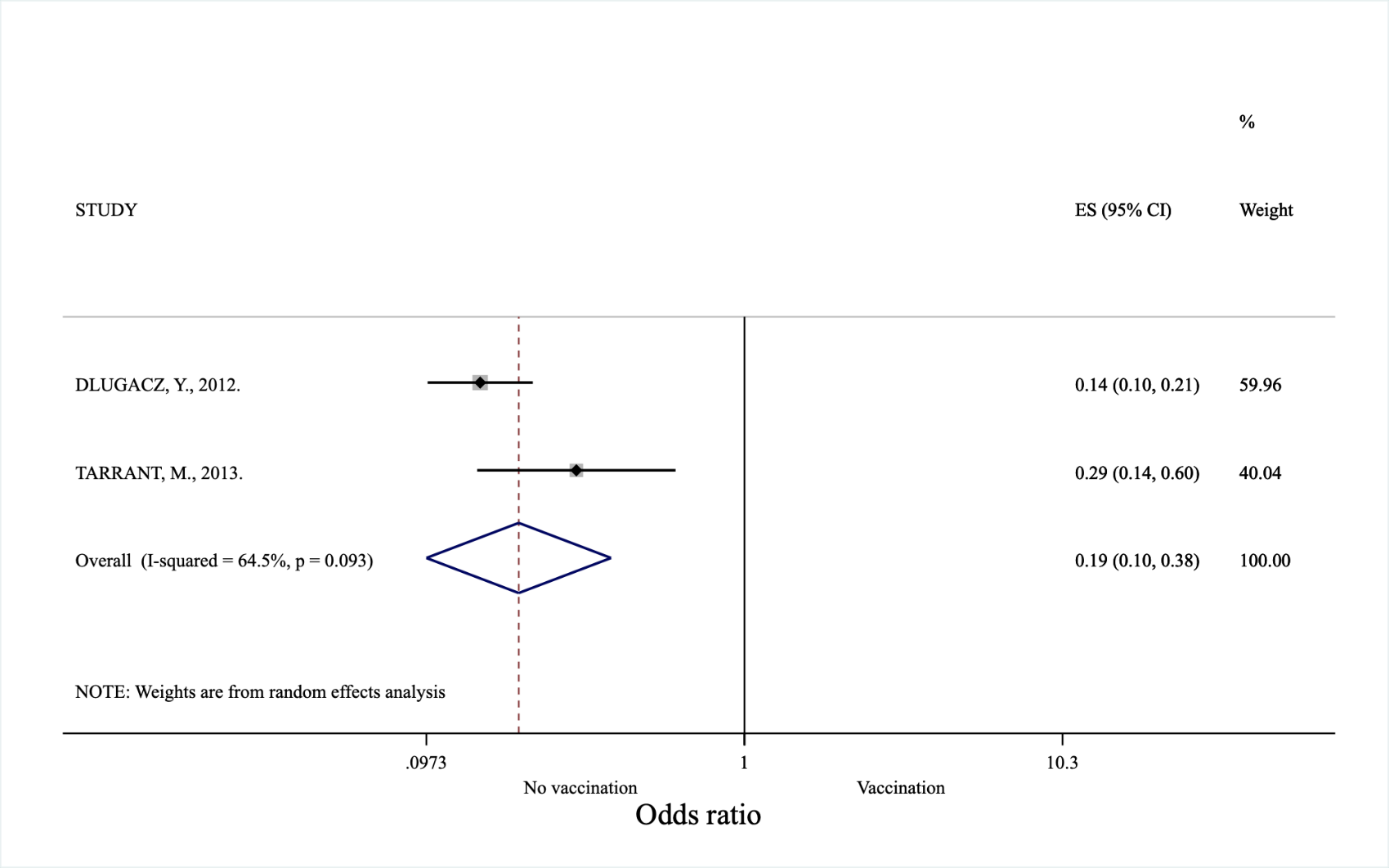


Figure 13 Forest plot for pandemic influenza vaccination and belief that vaccine can cause miscarriage or spontaneous abortion (vaccine harm in state of pregnancy)


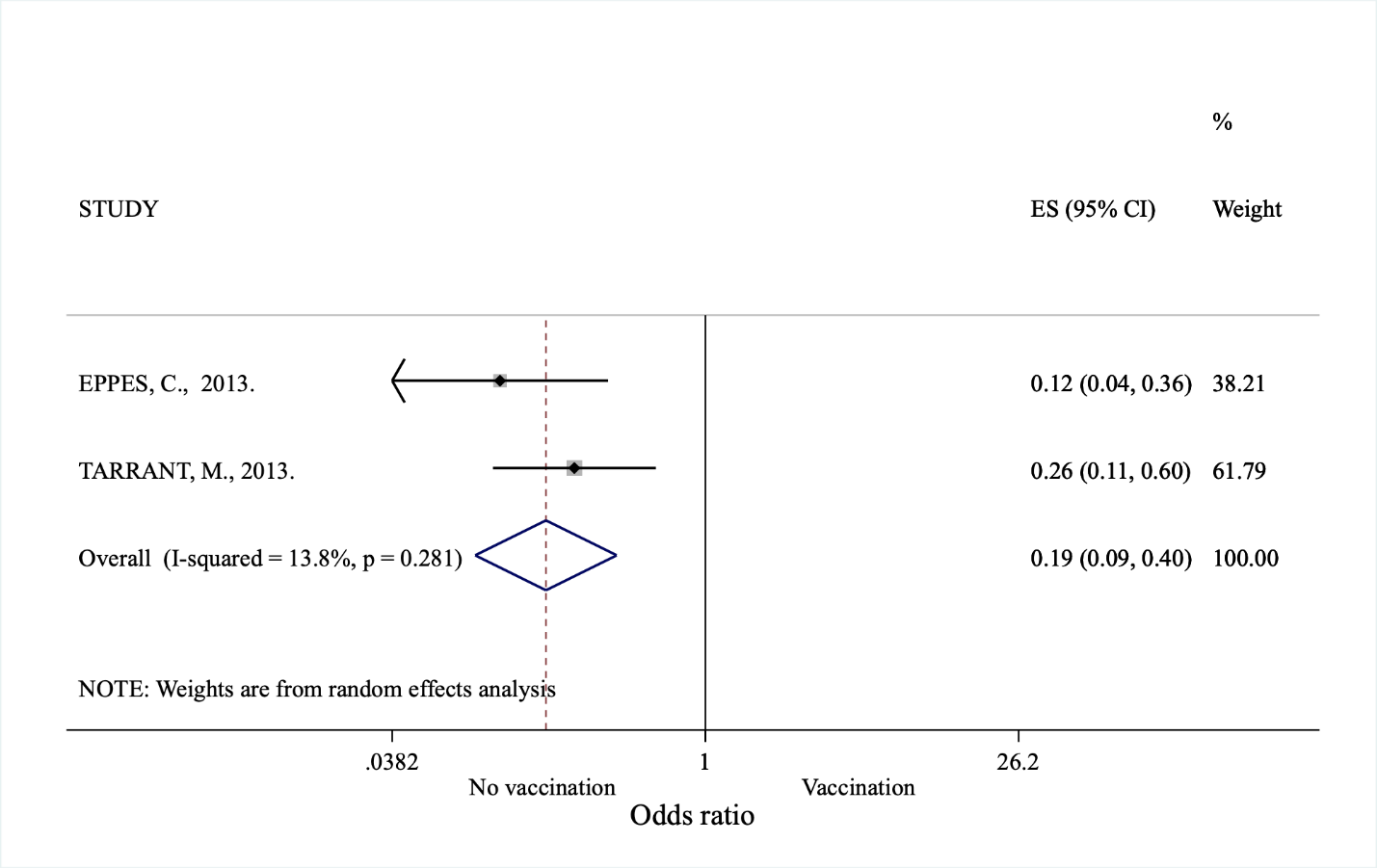


Figure 14 Forest plot for pandemic influenza vaccination and belief that vaccine causes birth defects (vaccine harm in state of pregnancy)


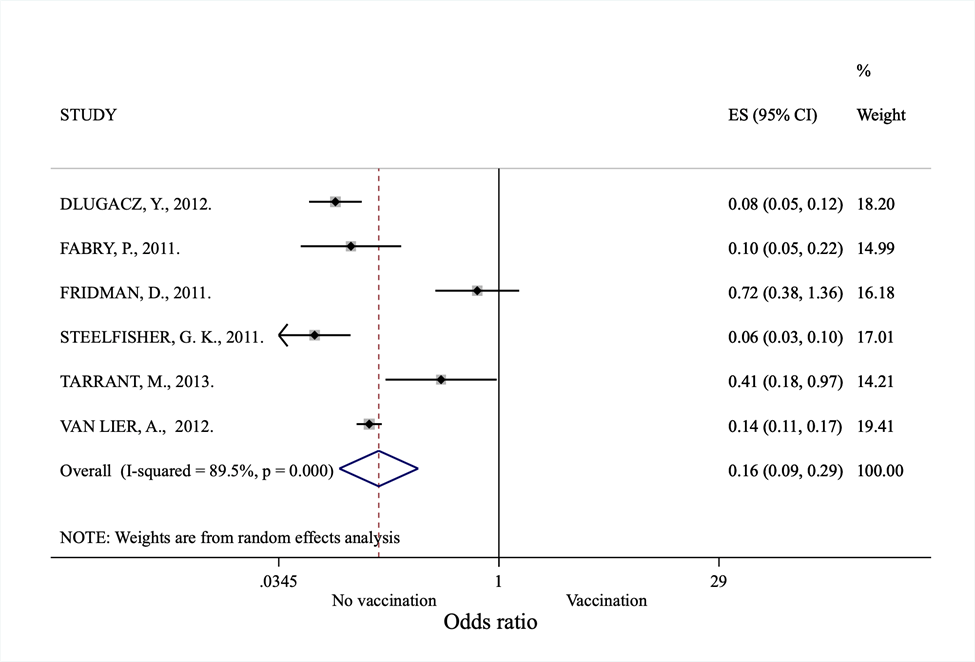


Figure 15 Forest plot for pandemic influenza vaccination and general belief that vaccine causes harm (vaccine harm in state of pregnancy)


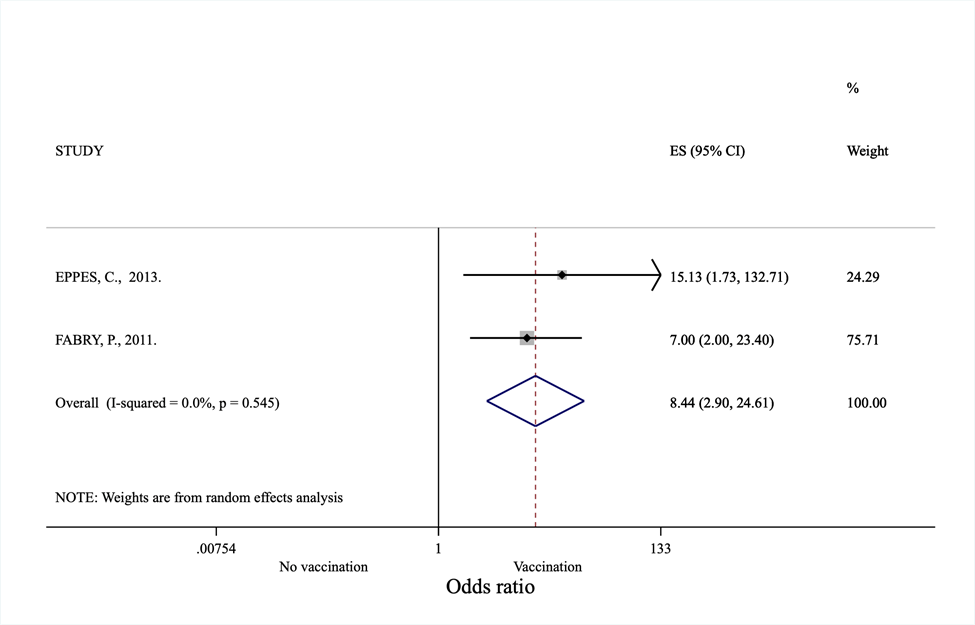


Figure 16 Forest plot for pandemic influenza vaccination and belief that vaccine effectively protects/benefits pregnant women (benefit of vaccines)


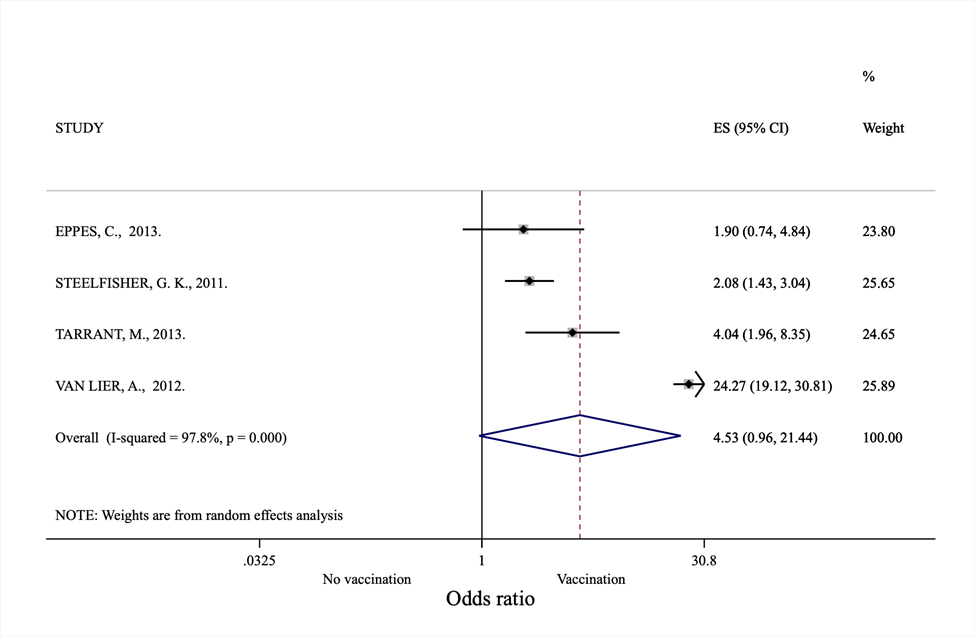


Figure 17 Forest plot for pandemic influenza vaccination and belief that vaccine effectively protects/benefits the baby (benefit of vaccines)


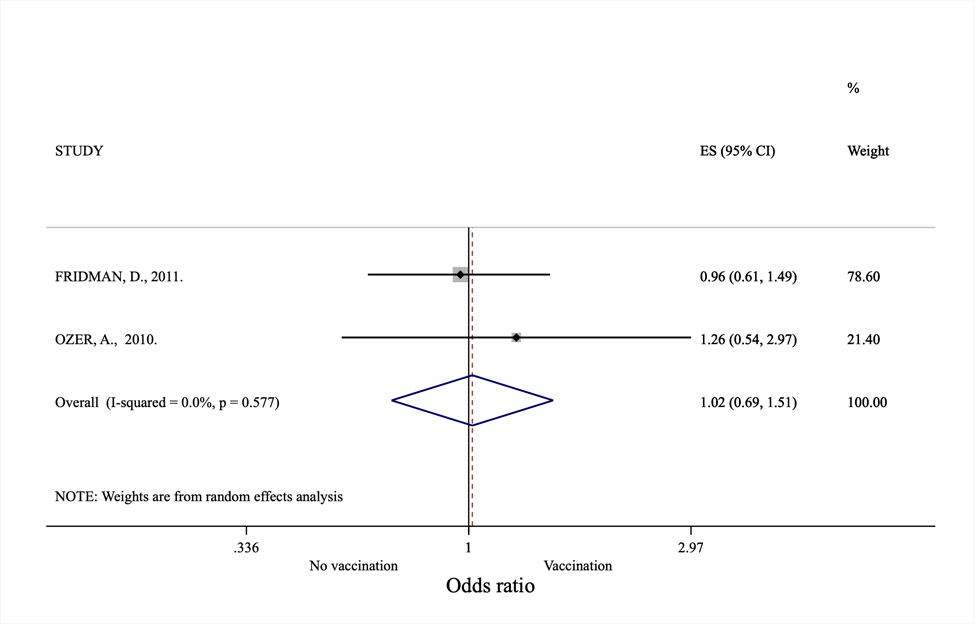


Figure 18 Forest plot for pandemic influenza vaccination and belief that vaccine is generally protective/beneficial (benefit of vaccines)


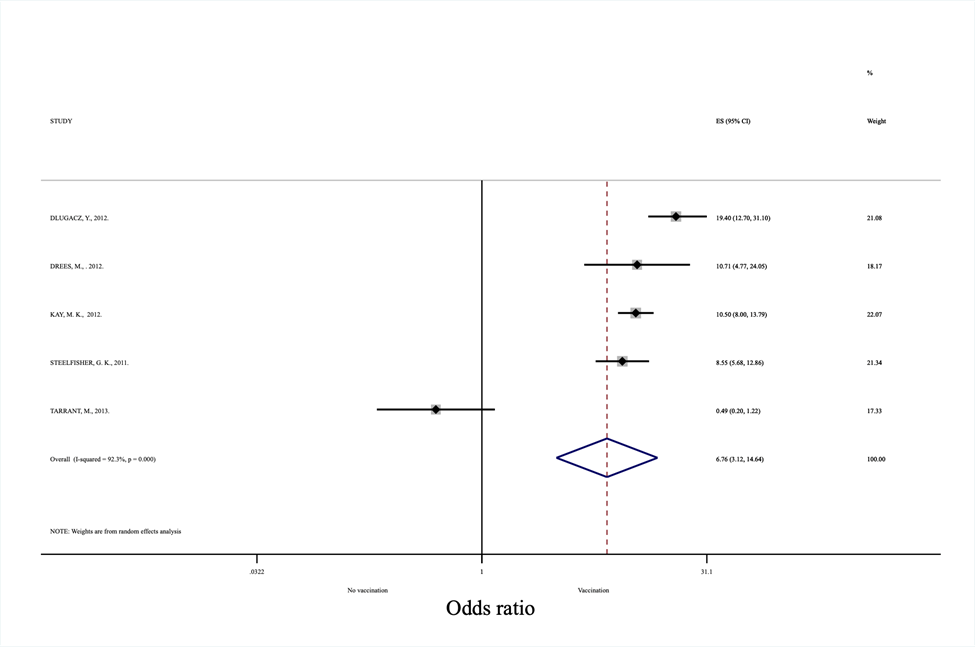


Figure 19 Forest plot for pandemic influenza vaccination and a recommendation from a healthcare professional (influence of HCP recommendation)


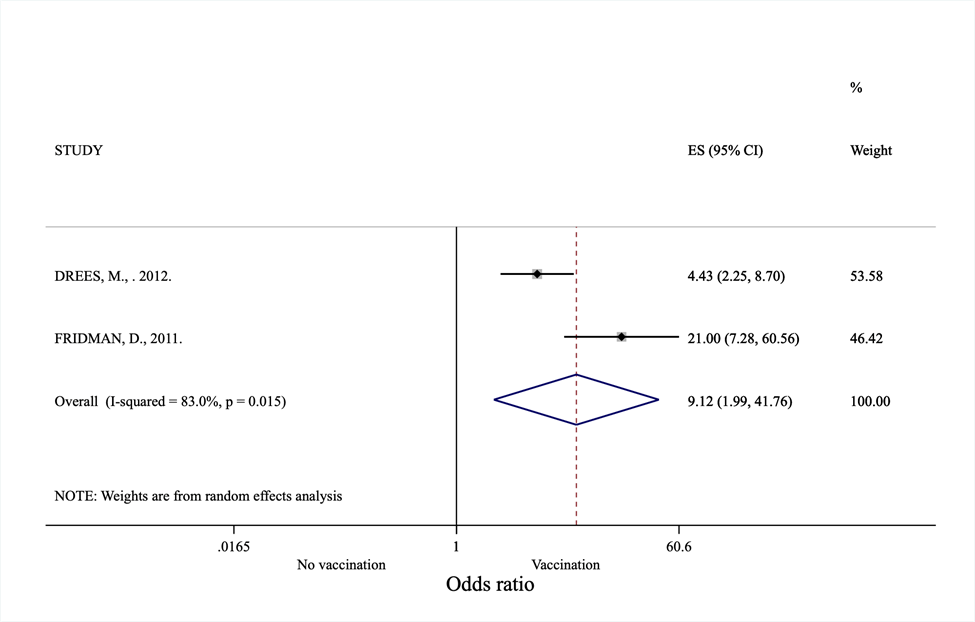


Figure 20 Forest plot for pandemic influenza vaccination and previous vaccination in pregnancy (personal history of vaccination)


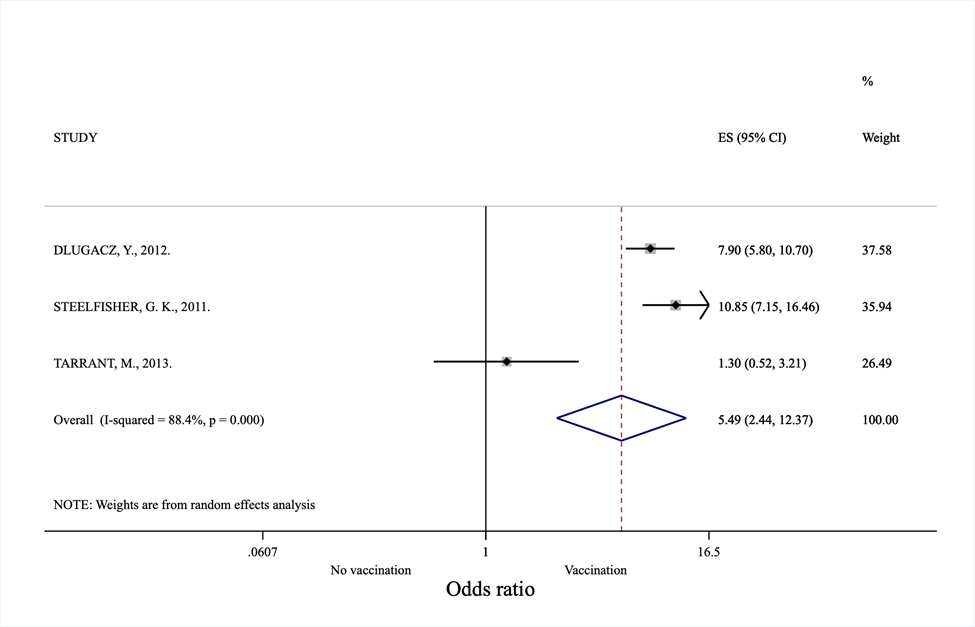


Figure 21 Forest plot for pandemic influenza vaccination and previous vaccination any time outside of pregnancy (personal history of vaccination)

# **Appendix 19. Individual study forest plot for Pertussis – primary outcome**


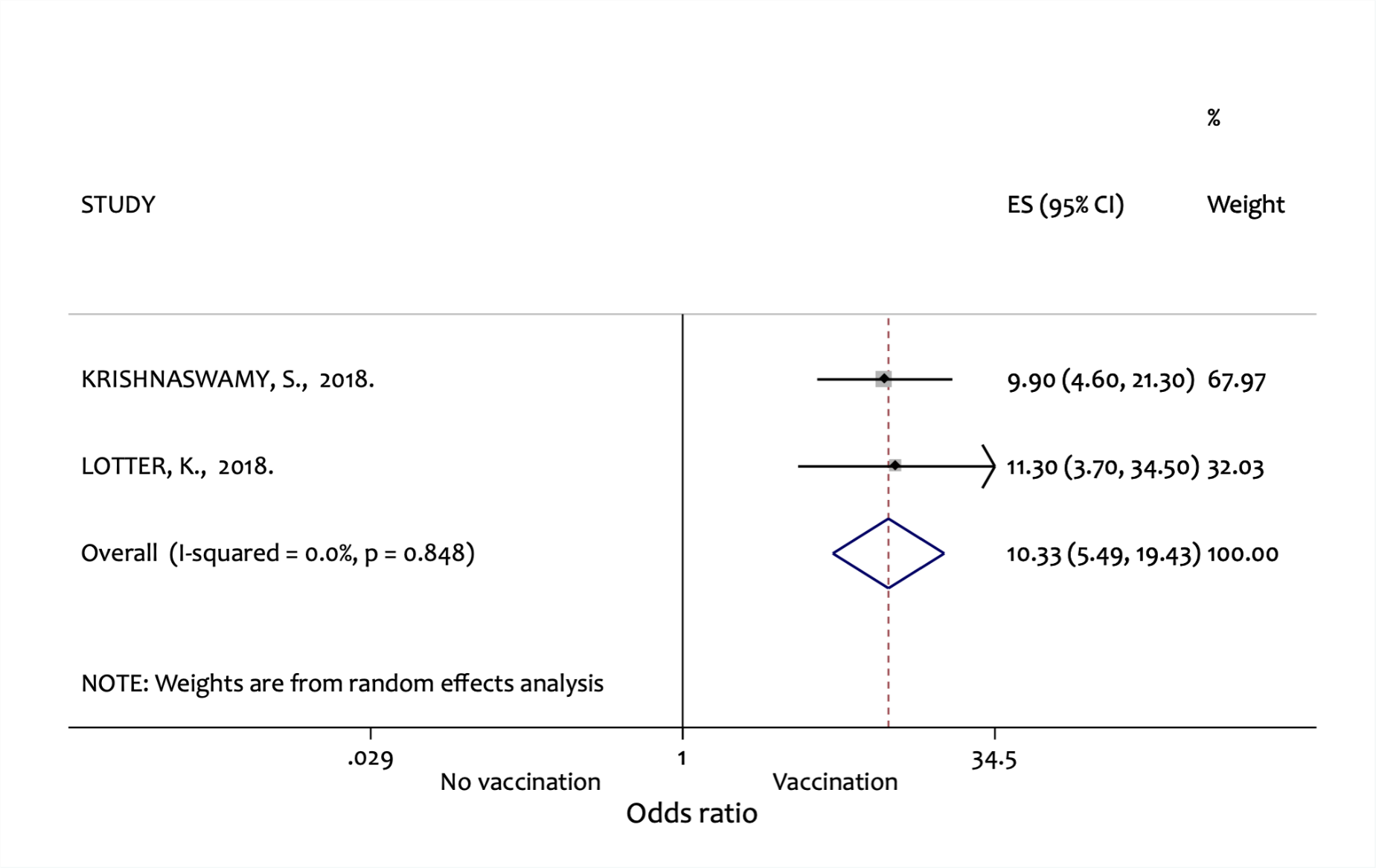


Figure 22 Forest plot for pertussis vaccination and a recommendation from a healthcare professional (influence of HCP recommendation)

# **Appendix 20. Individual study forest plot for Seasonal Influenza – primary outcome**


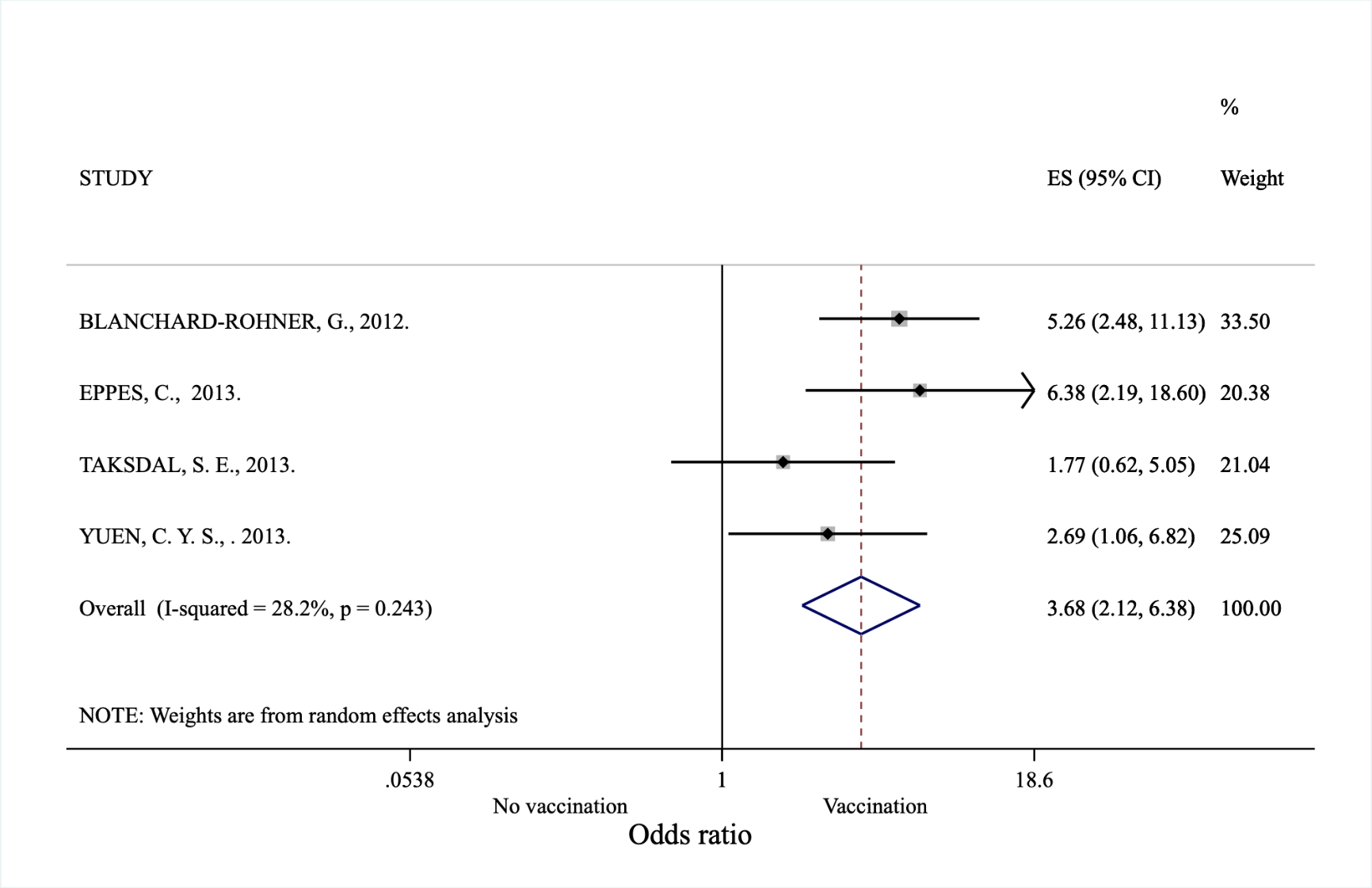


Figure 23 Forest plot for seasonal influenza vaccination and awareness of policy recommendation for vaccination during pregnancy (informed)


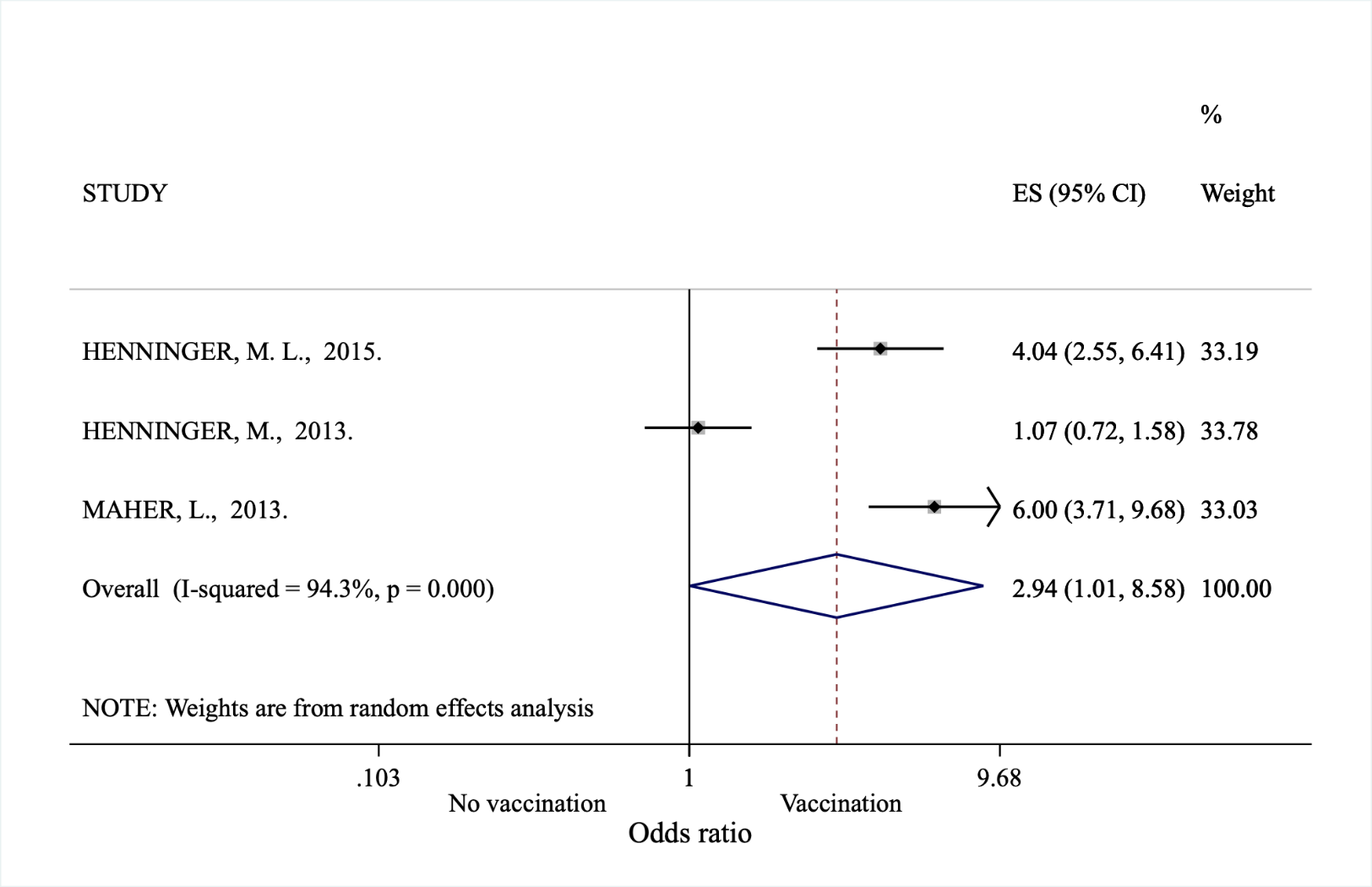


Figure 24 Forest plot for seasonal influenza vaccination and feeling informed to decide on vaccination during pregnancy (informed)


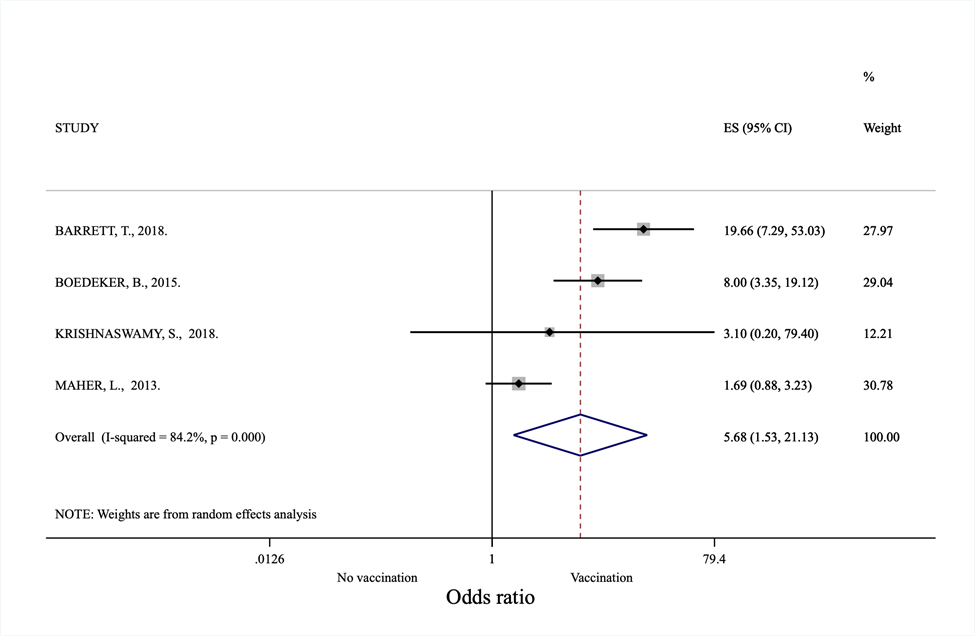


Figure 25 Forest plot for seasonal influenza vaccination and awareness of general information of vaccination for pregnant women (informed)


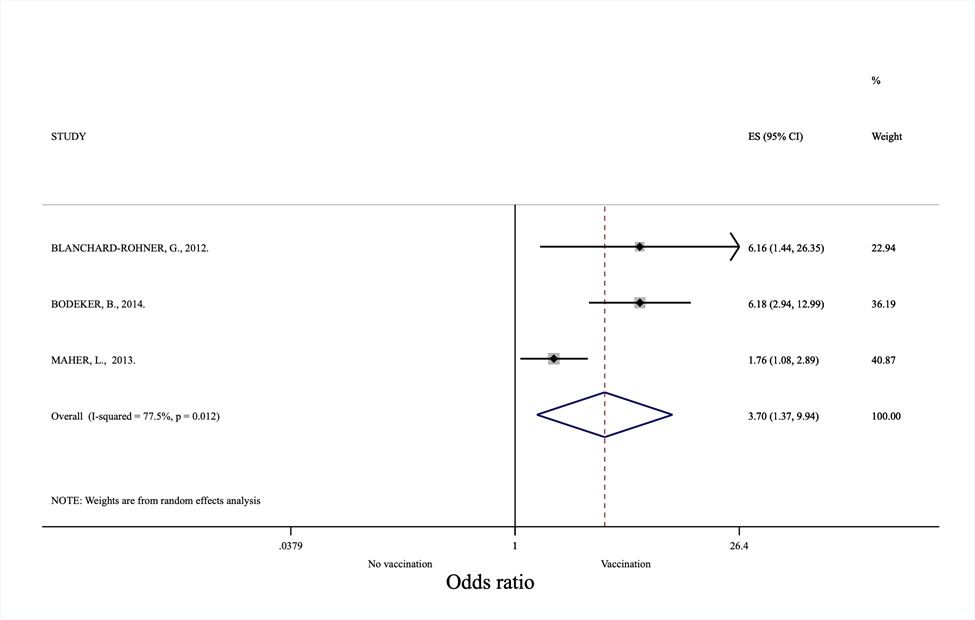


Figure 26 Forest plot for seasonal influenza vaccination and the perception the disease is harmful in pregnancy (disease severity)


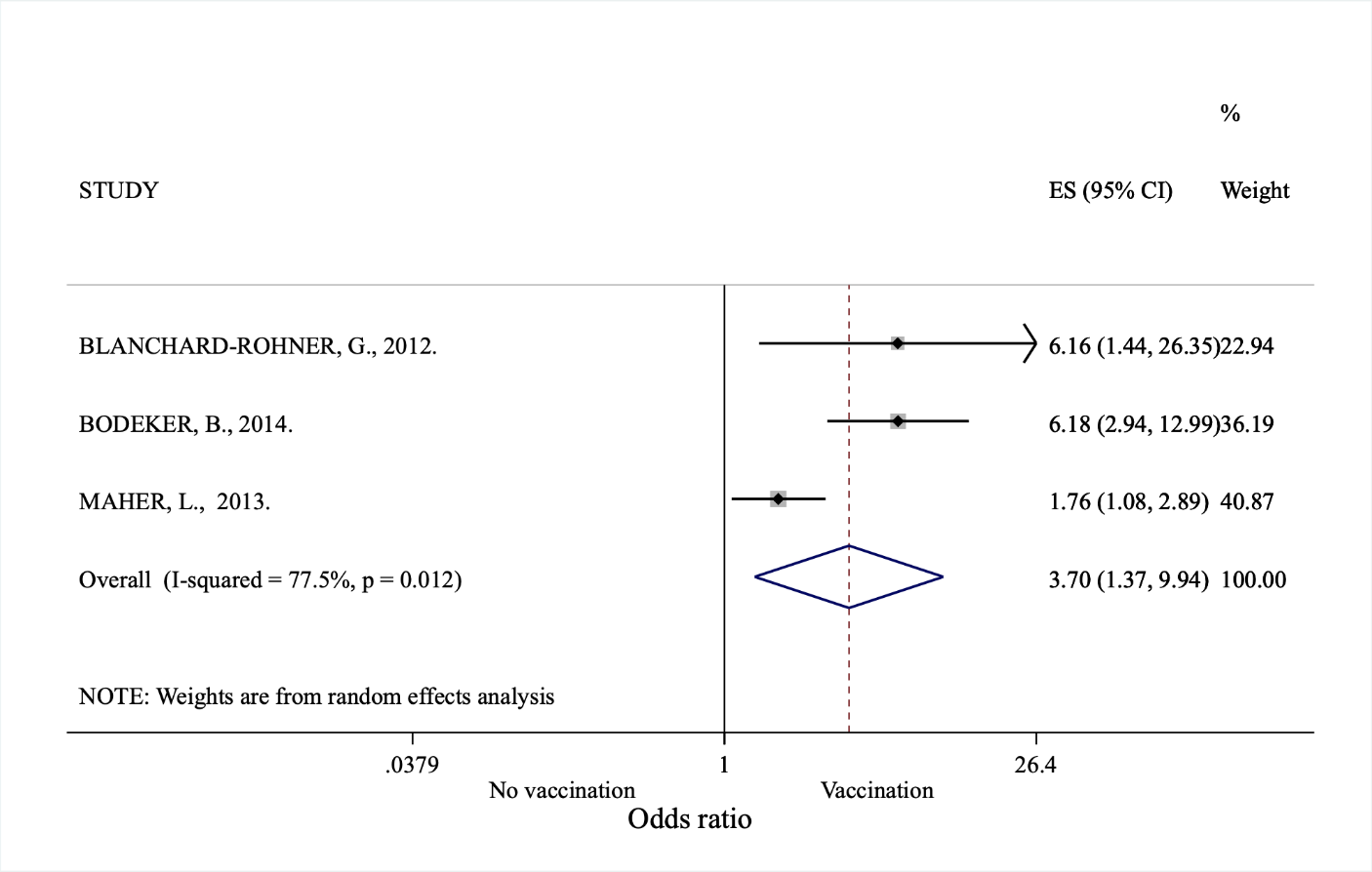


Figure 27 Forest plot for seasonal influenza vaccination and perception that disease may result in hospitalization (disease severity)


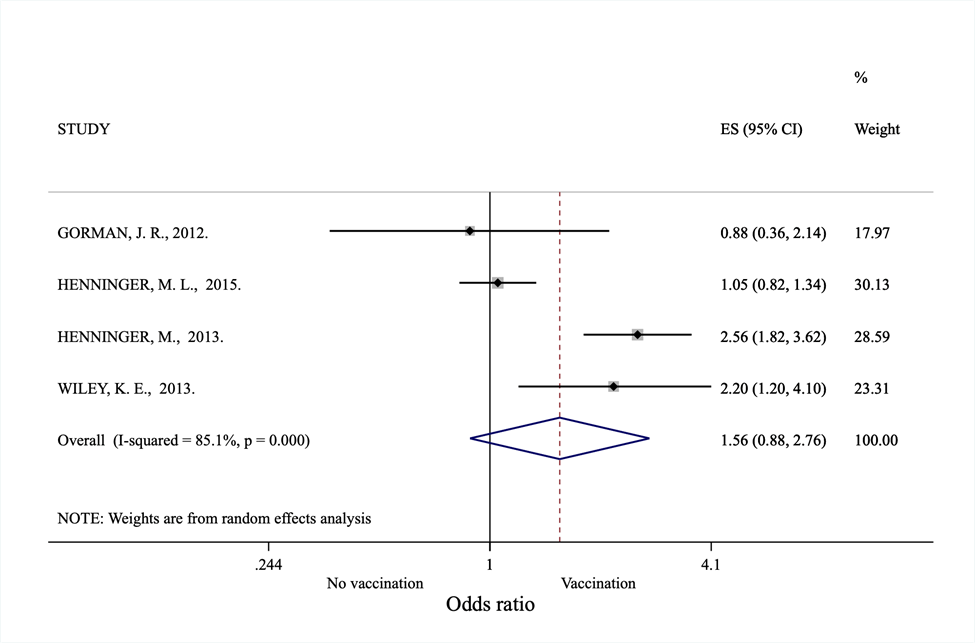


Figure 28 Forest plot for seasonal influenza vaccination and perception of general disease severity (disease severity)


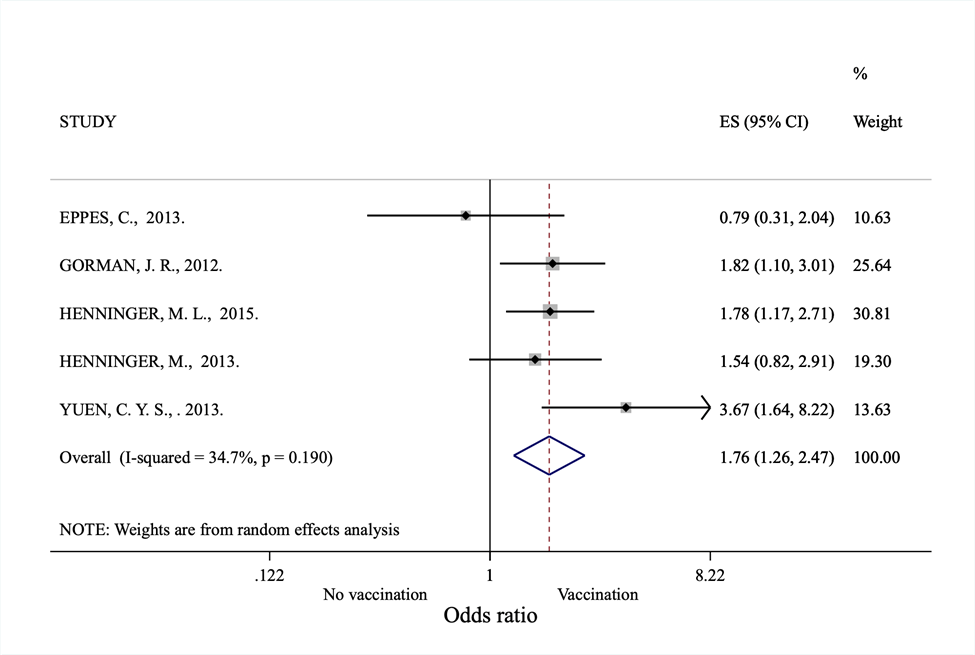


Figure 29 Forest plot for seasonal influenza vaccination and perception that pregnant women are at increased risk for disease (disease susceptibility)


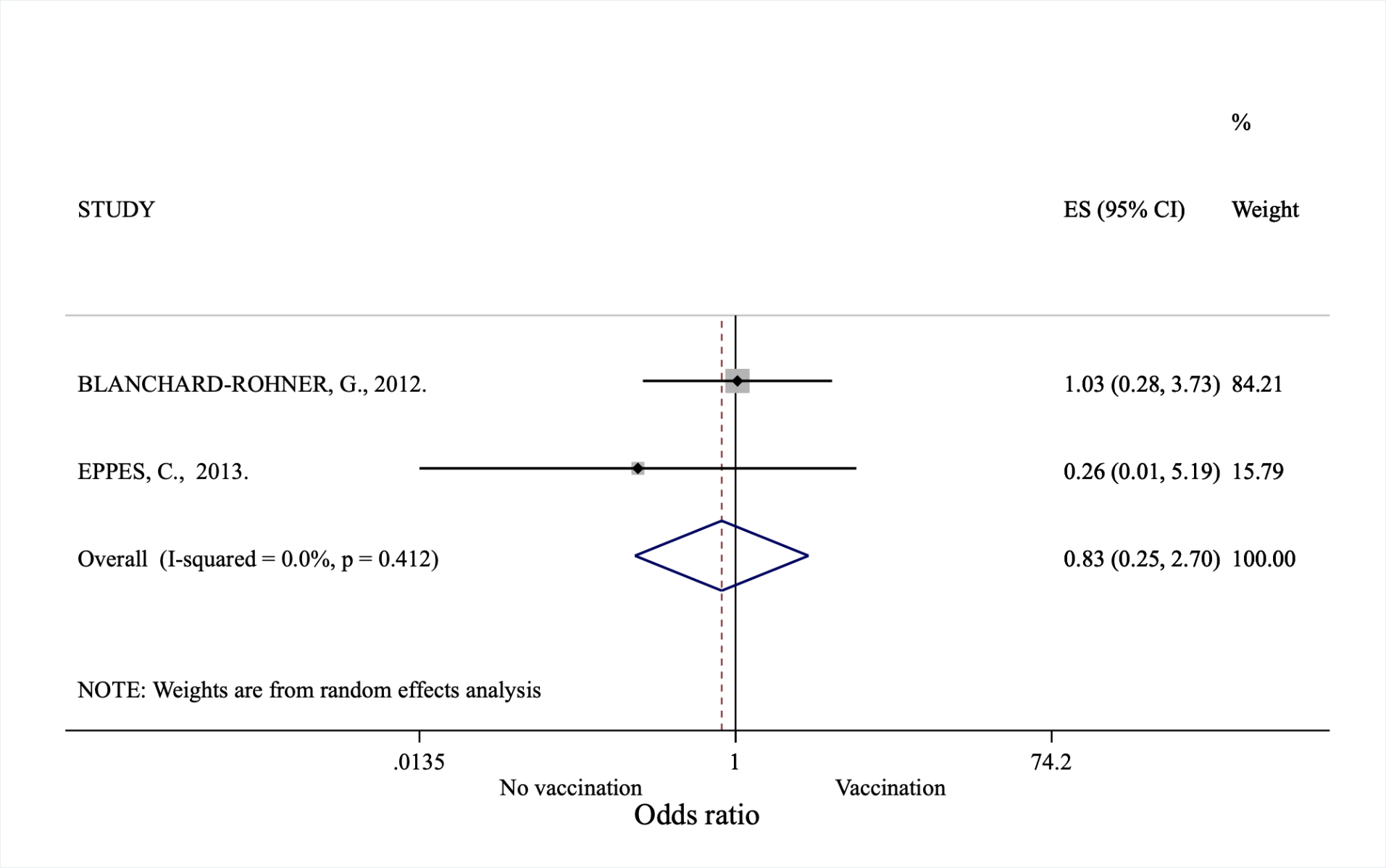


Figure 30 Forest plot for seasonal influenza vaccination and perception of disease contagiousness (disease susceptibility)


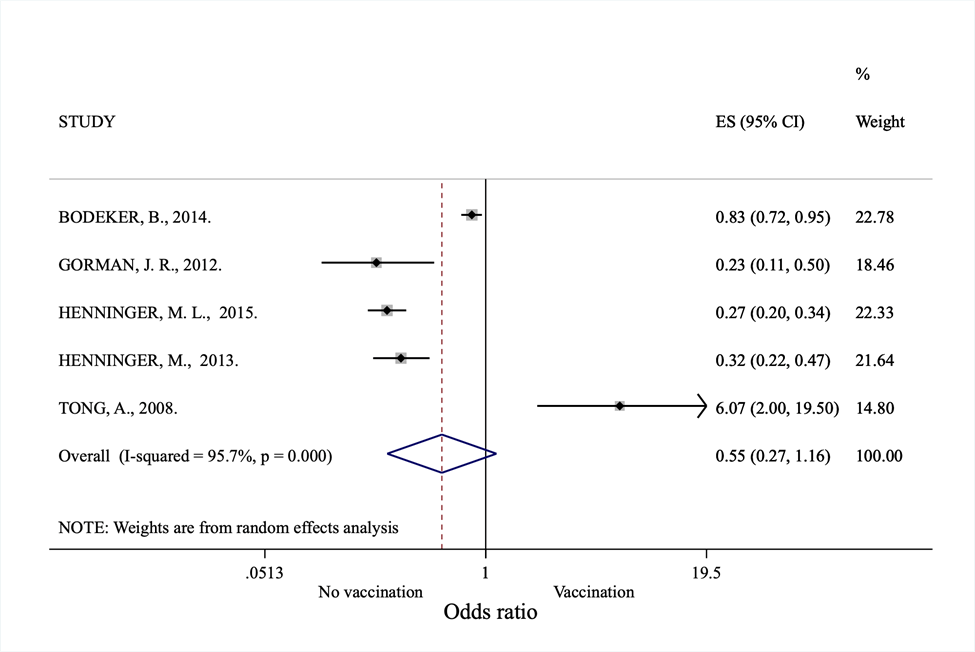


Figure 31 Forest plot for seasonal influenza vaccination and concern for or perceived danger of vaccine side-effects (vaccine side-effects)


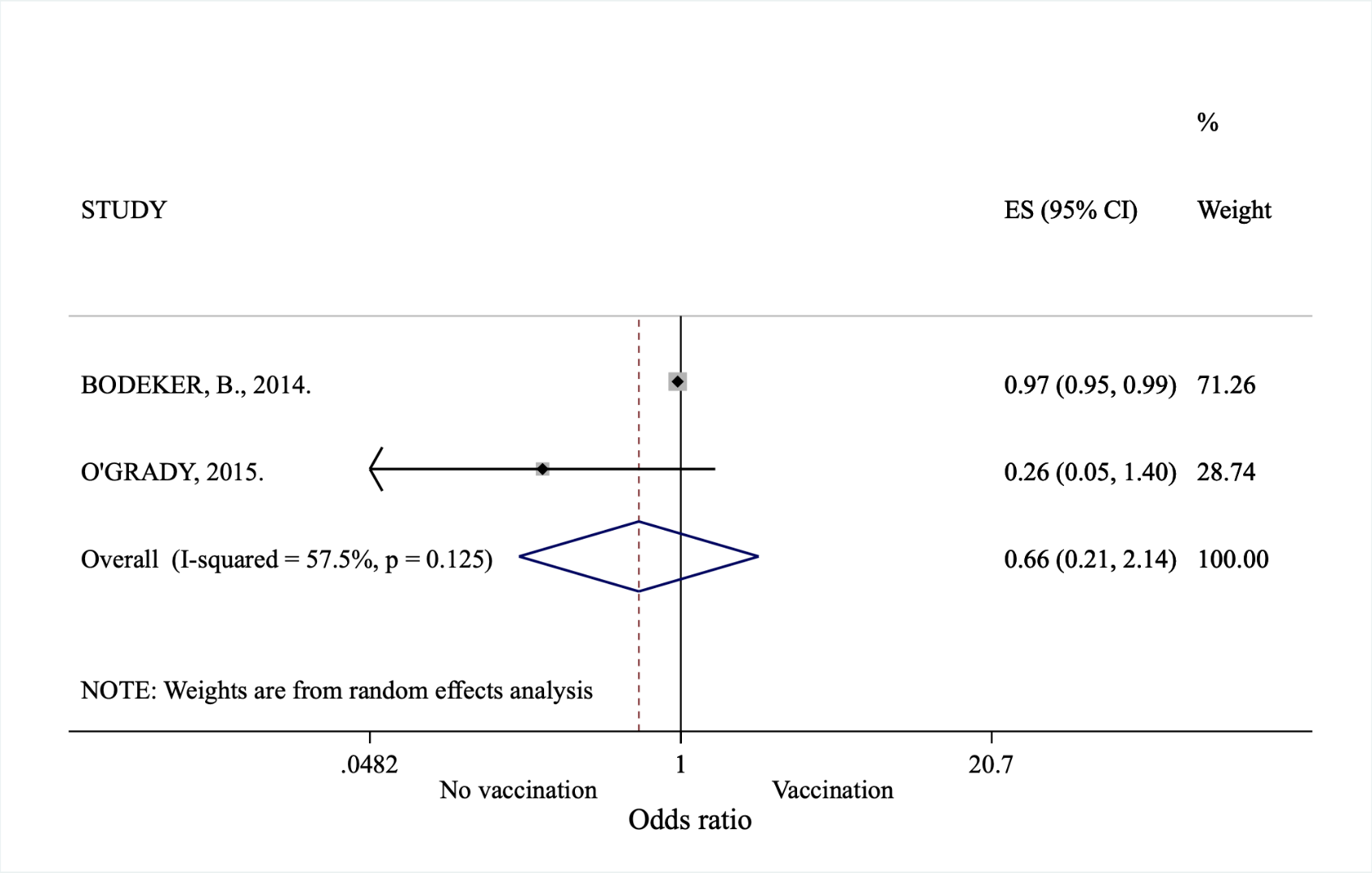


Figure 32 Forest plot for seasonal influenza vaccination and perceived high probability of vaccine side-effects (vaccine side-effects)


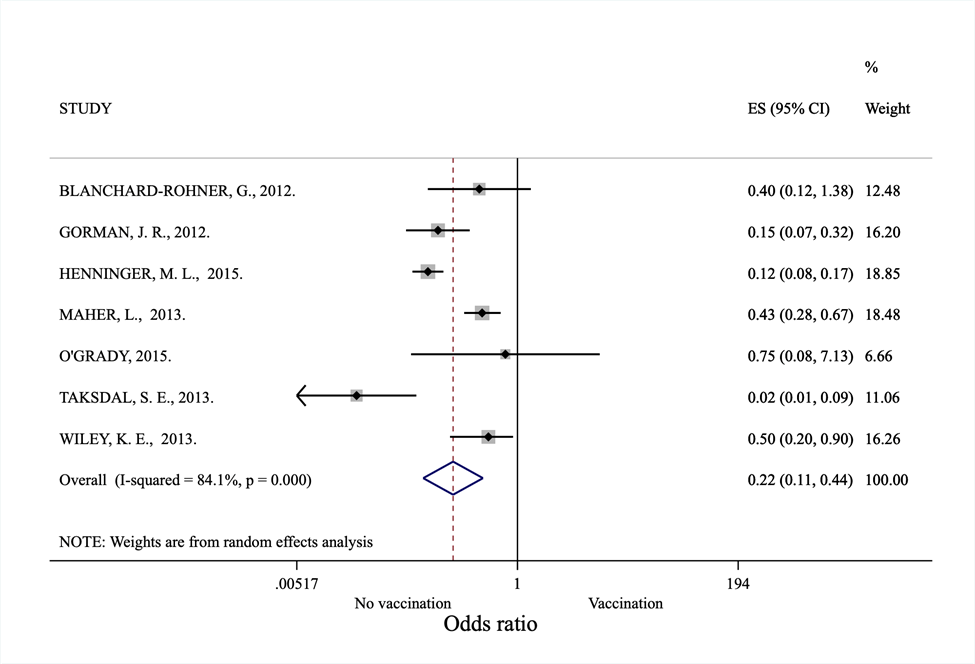


Figure 33 Forest plot for seasonal influenza vaccination and general belief that vaccine causes harm (vaccine harm in state of pregnancy)


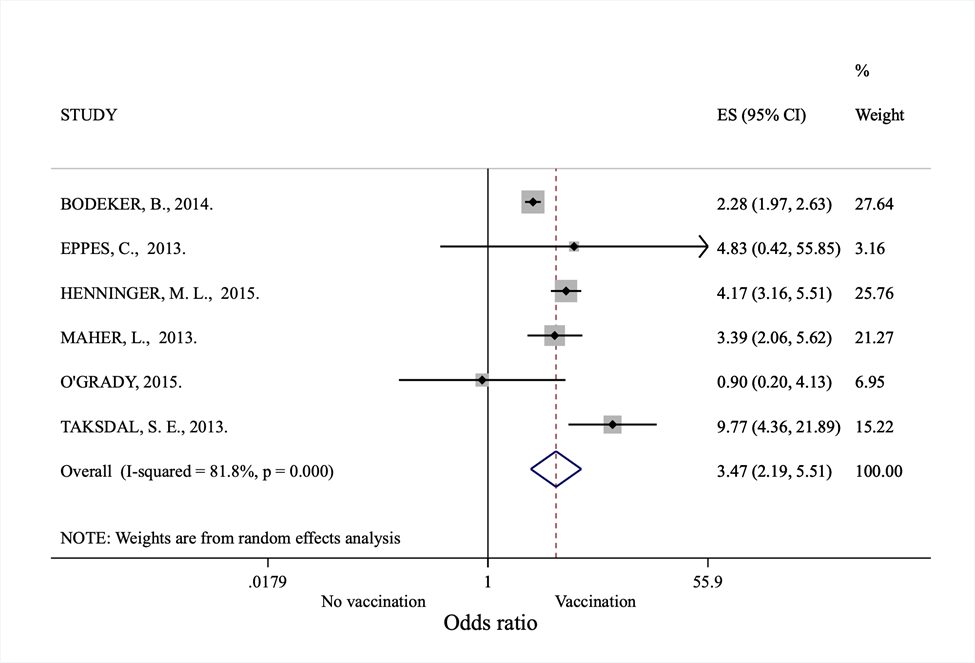


Figure 34 Forest plot for seasonal influenza vaccination and belief that vaccine effectively protects/benefits pregnant women (benefit of vaccines)


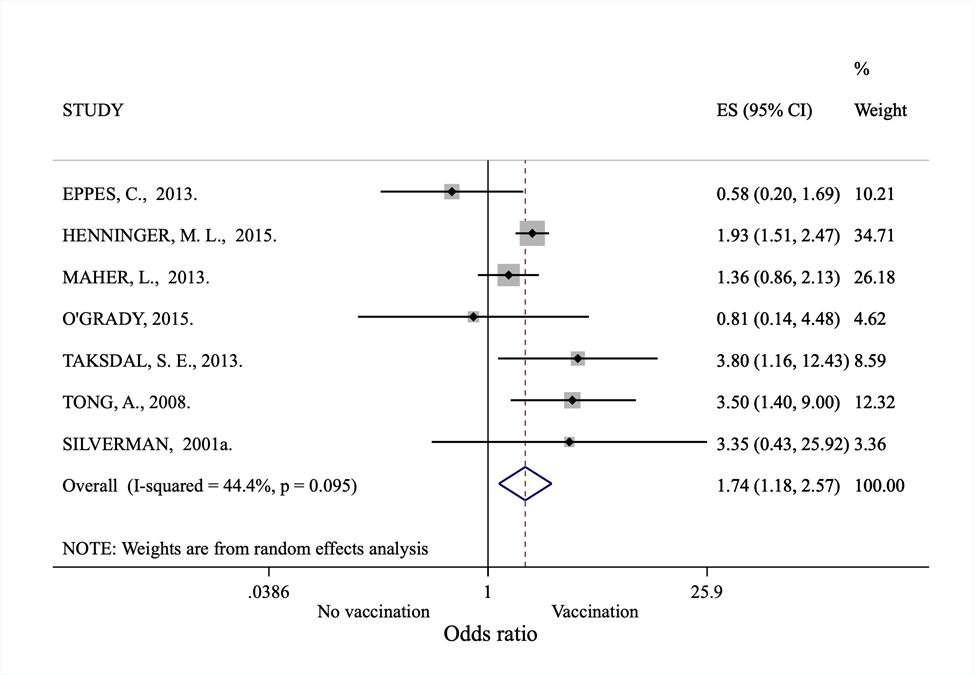


Figure 35 Forest plot for seasonal influenza vaccination and belief that vaccine effectively protects/benefits the baby (benefit of vaccines)


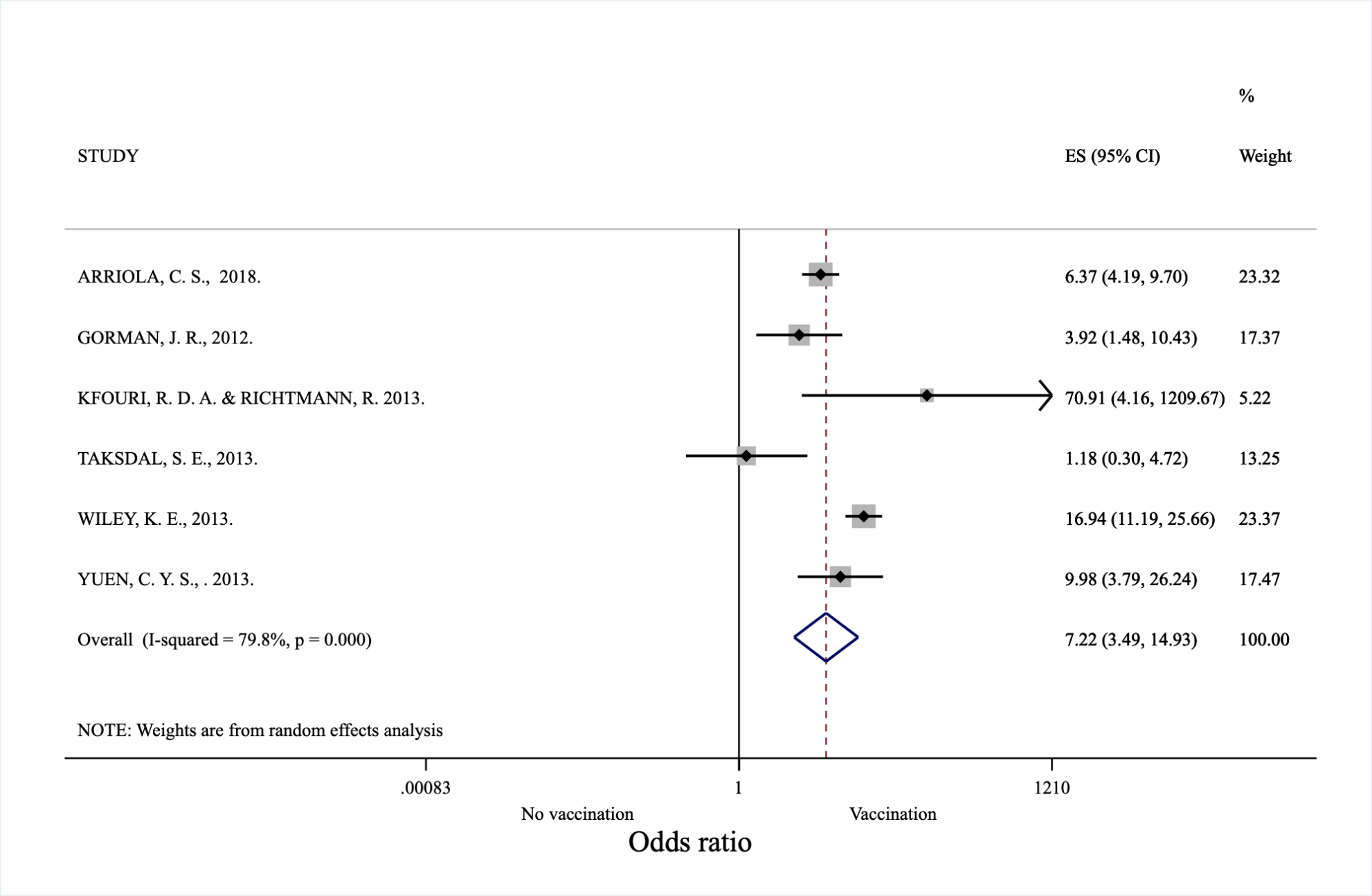


Figure 36 Forest plot for seasonal influenza vaccination and belief that vaccine is generally protective/beneficial (benefit of vaccines)


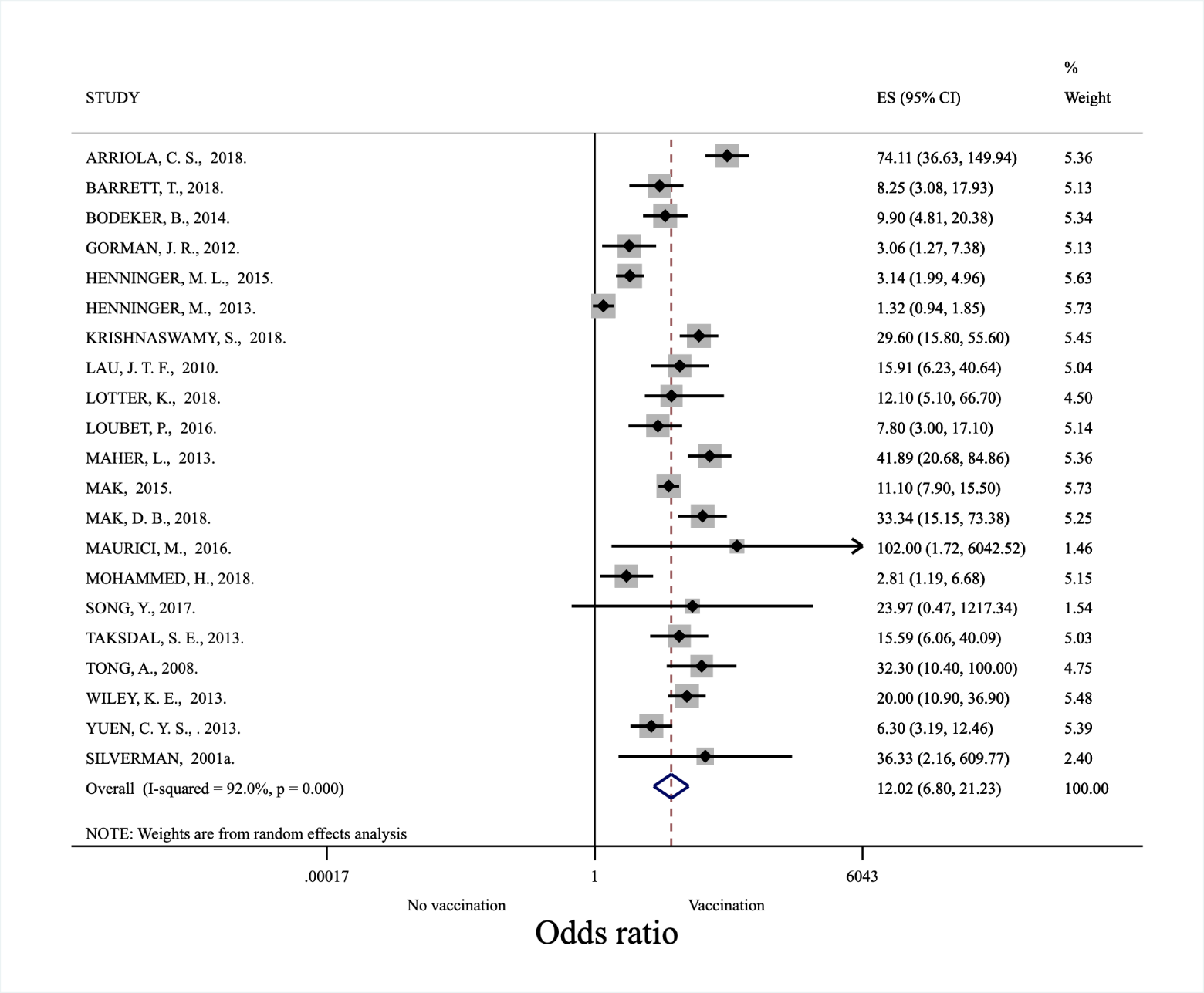


Figure 37 Forest plot for seasonal influenza vaccination and a recommendation from a healthcare professional (HCP recommendation)


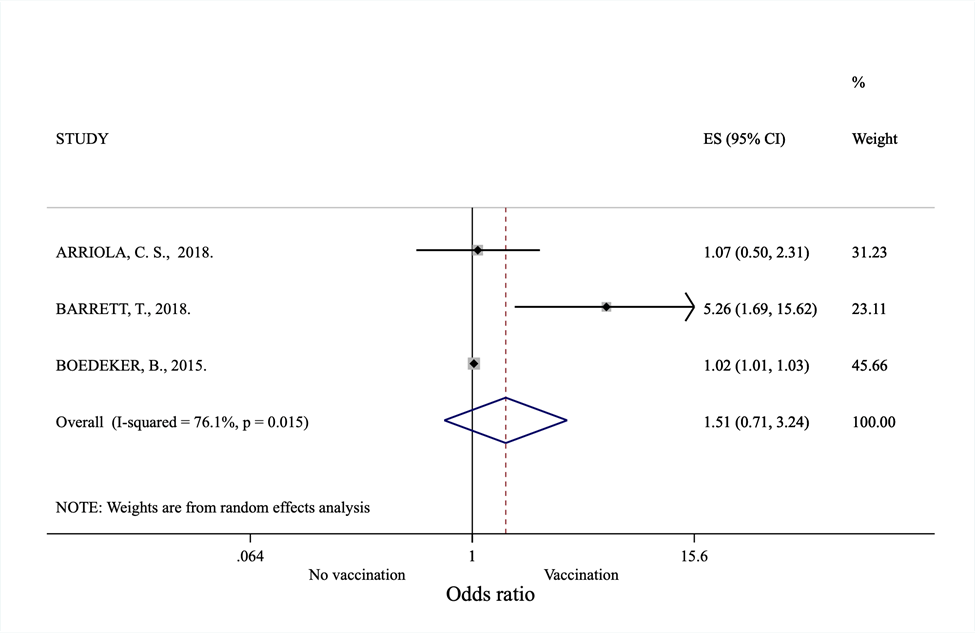


Figure 38 Forest plot for seasonal influenza vaccination and previous vaccination in pregnancy (personal history of vaccination)


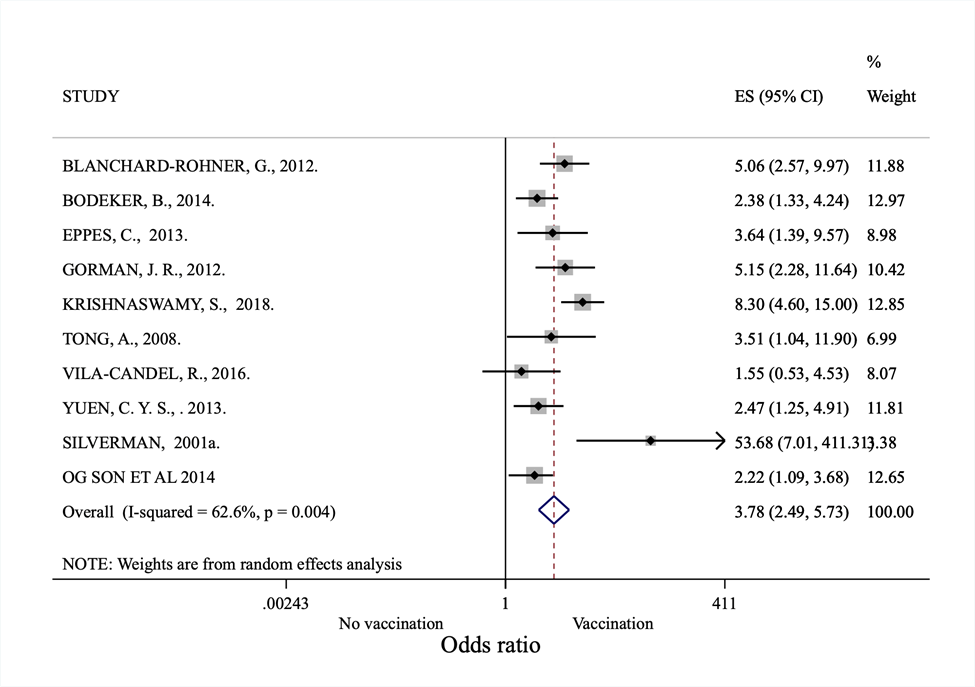


Figure 39 Forest plot for seasonal influenza vaccination and previous vaccination any time outside of pregnancy (personal history of vaccination)

**Appendix 21. Table – Summary ORs from meta-analyses investigating association between beliefs/experiences and vaccination**

| **Vaccine** | **Factor** | **Specific Factor** | **No. of Studies** | **No. of Respondents** | **OR (95% CI)** | **I-squared (%)** |
| --- | --- | --- | --- | --- | --- | --- |
| Pertussis | HCP Recommendation | hcw | 2 | 637 | 10.33 (5.49-19.43) | 0 |
| P.Flu | Awareness & Information | general | 2 | 3498 | 1.50 (1.06-2.12) | 70.46 |
| P.Flu | Disease Severity | general | 4 | 5948 | 2.04 (0.98-4.26) | 96.89 |
| P.Flu | Disease Severity | hospitalisation | 2 | 1060 | 2.91 (2.02-4.18) | 0 |
| P.Flu | Disease Susceptibility | pregnant | 5 | 4044 | 1.11 (0.56-2.19) | 94.86 |
| P.Flu | Vax Side-effect | concern | 2 | 760 | 0.44 (0.23-0.81) | 0 |
| P.Flu | Vax Side-effect | knowledge | 2 | 1325 | 0.27 (0.21-0.34) | 0 |
| P.Flu | Vaccine Harm During Pregnancy | birth | 2 | 629 | 0.19 (0.09-0.40) | 13.82 |
| P.Flu | Vaccine Harm During Pregnancy | general | 6 | 5525 | 0.16 (0.09-0.29) | 89.48 |
| P.Flu | Vaccine Harm During Pregnancy | miscarriage | 2 | 1574 | 0.19 (0.10-0.38) | 64.49 |
| P.Flu | Utility of Vaccines | baby | 4 | 4119 | 4.53 (0.96-21.44) | 97.82 |
| P.Flu | Utility of Vaccines | general | 2 | 526 | 1.02 (0.69-1.51) | 0 |
| P.Flu | Utility of Vaccines | pregnant | 2 | 338 | 8.44 (2.90-24.61) | 0 |
| P.Flu | HCP Recommendation | hcw | 5 | 6898 | 6.76 (3.12-14.64) | 92.28 |
| P.Flu | History of previous vaccination | anytime | 3 | 2387 | 5.49 (2.44-12.37) | 88.44 |
| P.Flu | History of previous vaccination | during | 2 | 442 | 9.12 (1.99-41.76) | 83.04 |
| S.Flu | Awareness & Information | general | 4 | 1193 | 5.68 (1.53-21.13) | 84.15 |
| S.Flu | Awareness & Information | informed | 3 | 2119 | 2.94 (1.01-8.58) | 94.32 |
| S.Flu | Awareness & Information | policy | 4 | 3583 | 3.68 (2.12-6.38) | 28.23 |
| S.Flu | Disease Severity | general | 4 | 2671 | 1.56 (0.88-2.76) | 85.08 |
| S.Flu | Disease Severity | harmful | 3 | 1748 | 3.70 (1.37-9.94) | 77.54 |
| S.Flu | Disease Severity | hospitalisation | 2 | 346 | 0.57 (0.22-1.45) | 0 |
| S.Flu | Disease Susceptibility | contagiousness | 2 | 346 | 0.83 (0.25-2.70) | 0 |
| S.Flu | Disease Susceptibility | pregnant | 5 | 4763 | 1.76 (1.26-2.47) | 34.72 |
| S.Flu | Vax Side-effect | concern | 5 | 3066 | 0.55 (0.27-1.16) | 95.65 |
| S.Flu | Vax Side-effect | probability | 2 | 1076 | 0.66 (0.21-2.14) | 57.47 |
| S.Flu | Vaccine Harm During Pregnancy | general | 7 | 3200 | 0.22 (0.11-0.44) | 84.08 |
| S.Flu | Utility of Vaccines | baby | 7 | 2546 | 1.74 (1.18-2.57) | 44.36 |
| S.Flu | Utility of Vaccines | general | 6 | 5814 | 7.22 (3.49-14.93) | 79.77 |
| S.Flu | Utility of Vaccines | pregnant | 6 | 3144 | 3.47 (2.19-5.51) | 81.79 |
| S.Flu | HCP Recommendation | hcw | 21 | 14099 | 12.02 (6.80-21.23) | 92.04 |
| S.Flu | History of previous vaccination | anytime | 10 | 5768 | 3.78 (2.49-5.73) | 62.6 |
| S.Flu | History of previous vaccination | during | 3 | 2339 | 1.51 (0.71-3.24) | 76.12 |

| P.Flu = Pandemic Influenza, S.Flu = Seasonal Influenza |
| --- |

# **Appendix 22. Table – Summary ORs from quality sensitivity analysis: meta-analyses investigating association between beliefs/experiences and vaccination in studies with JBI score > 10**

| **Vaccine** | **Factor** | **Specific factor** | **No. of studies** | **No. of respondents** | **OR (95% CI)** | **I-squared (%)** |
| --- | --- | --- | --- | --- | --- | --- |
| P.Flu | Disease Severity | Perception of general disease severity | 2 | 462 | 1.57 (0.73-3.41) | 56.89 |
| P.Flu | Disease Susceptibility | Perception that pregnant women are at increased risk for disease | 2 | 462 | 2.04 (0.62-6.68) | 92.23 |
| P.Flu | Vaccine Harm During Pregnancy | Belief that vaccine causes general harm | 3 | 1,007 | 0.32 (0.10-1.02) | 86.24 |
| P.Flu | HCP Recommendation | Recommended by HCP | 2 | 854 | 2.32 (0.11-47.19) | 95.96 |
| P.Flu | History of previous vaccination | History of vaccination in previous pregnancy | 2 | 442 | 9.12 (1.99-41.76) | 83.04 |
| S.Flu | Awareness & Information | Awareness of general information on vaccination for pregnant woman | 2 | 656 | 5.60 (0.51-61.94) | 93.92 |
| S.Flu | Awareness & Information | Felt informed to decide on vaccination during pregnancy | 3 | 2,119 | 2.94 (1.01-8.58) | 94.32 |
| S.Flu | Awareness & Information | Awareness of policy recommendation for vaccination during pregnancy | 3 | 3,498 | 3.18 (1.68-6.01) | 33.76 |
| S.Flu | Disease Severity | Perception of general disease severity | 2 | 1,657 | 1.63 (0.68-3.90) | 94.15 |
| S.Flu | Disease Severity | Perception that disease is harmful to pregnancy | 3 | 1,748 | 3.70 (1.37-9.94) | 77.54 |
| S.Flu | Disease Susceptibility | Perception that pregnant women are at increased risk for disease | 3 | 4,479 | 1.99 (1.30-3.04) | 34.96 |
| S.Flu | Vaccine Harm During Pregnancy | Belief that vaccine causes general harm | 4 | 2,135 | 0.16 (0.06-0.46) | 90.07 |
| S.Flu | Benefit of Vaccines | Belief that vaccine protects/benefits baby | 4 | 2,168 | 1.96 (1.37-2.80) | 40.82 |
| S.Flu | Benefit of Vaccines | Belief that vaccine protects/benefits pregnant woman | 4 | 3,008 | 4.96 (1.98-12.40) | 69.19 |
| S.Flu | Benefit of Vaccines | Belief that vaccine is generally protective/beneficial | 3 | 4,541 | 3.82 (2.33-6.24) | 88.11 |
| S.Flu | HCP Recommendation | Recommended by HCP | 14 | 9,299 | 11.02 (5.48-22.18) | 93.92 |
| S.Flu | History of previous vaccination | History of vaccination outside of pregnancy | 4 | 4,287 | 3.07 (2.11-4.45) | 8.15 |
| S.Flu | History of previous vaccination | History of vaccination in previous pregnancy | 2 | 1,501 | 2.25 (0.47-10.67) | 81.3 |
| P.Flu = Pandemic Influenza, S.Flu = Seasonal Influenza | | | | | | |

# **Appendix 23. Table – Summary ORs from secondary analysis: meta-analyses investigating association between beliefs/experiences and intention to vaccinate**

| **Vaccine** | **Factor** | **Specific Factor** | **No. of Studies** | **No. of Respondents** | **OR (95% CI)** | **I-squared (%)** |
| --- | --- | --- | --- | --- | --- | --- |
| Pertussis | Awareness & Information | general | 2 | 591 | 3.31 (1.96-5.59) | 0 |
| Pertussis | Disease Susceptibility | baby | 2 | 634 | 2.02 (1.30-3.15) | 0 |
| Pertussis | Disease Susceptibility | pregnant | 2 | 531 | 5.13 (1.53-17.15) | 54.36 |
| Pertussis | Vaccine Harm During Pregnancy | general | 2 | 529 | 0.27 (0.03-2.39) | 91.72 |
| Pertussis | Utility of Vaccines | baby | 3 | 958 | 4.67 (2.86-7.62) | 0 |
| Pertussis | History of previous vaccination | during | 3 | 951 | 2.24 (1.42-3.52) | 0 |
| P.Flu | Disease Susceptibility | pregnant | 2 | 584 | 4.52 (2.20-9.29) | 50.68 |
| S.Flu | Disease Severity | general | 2 | 1588 | 0.74 (0.36-1.53) | 64.73 |
| S.Flu | Disease Severity | harmful | 2 | 1588 | 1.58 (0.51-4.90) | 98.05 |
| S.Flu | Disease Susceptibility | baby | 2 | 1879 | 2.97 (0.96-9.23) | 95.65 |
| S.Flu | Disease Susceptibility | pregnant | 2 | 1879 | 2.99 (1.16-7.68) | 94.55 |
| S.Flu | Vaccine Harm During Pregnancy | general | 3 | 2200 | 0.45 (0.25-0.81) | 75.41 |
| S.Flu | Utility of Vaccines | baby | 3 | 2149 | 3.02 (1.05-8.71) | 95.14 |
| S.Flu | Utility of Vaccines | foetus | 2 | 1879 | 2.59 (1.65-4.07) | 74.78 |
| S.Flu | Utility of Vaccines | woman | 3 | 2215 | 2.79 (1.45-5.34) | 84.66 |
| P.Flu = Pandemic Influenza, S.Flu = Seasonal Influenza | | | | | | |

# **Appendix 24. Table – Summary ORs from secondary analysis: meta-analyses investigating association between beliefs/experiences and intention to vaccinate in studies with JBI score > 10**

| **Vaccine** | **Factor** | **Specific factor** | **No. of studies** | **No. of respondents** | **OR (95% CI)** | **I-squared (%)** |
| --- | --- | --- | --- | --- | --- | --- |
| Pertussis | History of previous vaccination | History of vaccination in previous pregnancy | 2 | 734 | 2.55 (1.20-5.39) | 25.75 |
| P.Flu | Disease Susceptibility | Perception that pregnant women are at increased risk for disease | 2 | 584 | 4.52 (2.20-9.29) | 50.68 |
| S.Flu | Disease Severity | Perception of general disease severity | 2 | 1,588 | 0.74 (0.36-1.53) | 64.73 |
| S.Flu | Disease Severity | Perception that disease is harmful to pregnancy | 2 | 1,588 | 1.58 (0.51-4.90) | 98.05 |
| S.Flu | Disease Susceptibility | Perception that baby is at risk for disease | 2 | 1,879 | 2.97 (0.96-9.23) | 95.65 |
| S.Flu | Disease Susceptibility | Perception that pregnant women are at increased risk for disease | 2 | 1,879 | 2.99 (1.16-7.68) | 94.55 |
| S.Flu | Vaccine Harm During Pregnancy | Belief that vaccine causes general harm | 3 | 2,200 | 0.45 (0.25-0.81) | 75.41 |
| S.Flu | Benefit of Vaccines | Belief that vaccine protects/benefits baby | 2 | 1,879 | 1.85 (0.65-5.25) | 95.53 |
| S.Flu | Benefit of Vaccines | Belief that vaccine protects/benefits foetus | 2 | 1,879 | 2.59 (1.65-4.07) | 74.78 |
| S.Flu | Benefit of Vaccines | Belief that vaccine protects/benefits pregnant woman | 3 | 2,215 | 2.79 (1.45-5.34) | 84.66 |
| P.Flu = Pandemic Influenza, S.Flu = Seasonal Influenza | | | | | | |

# **Appendix 25: Table –OR from one study meta-analyses investigating association between beliefs/experiences and vaccination in studies**

| **Vaccine** | **Factor** | **Specific Factor** | **No. of Studies** | **No. of Respondents** | **OR (95% CI)** | **I-squared (%)** |
| --- | --- | --- | --- | --- | --- | --- |
| Pertussis | Awareness & Information | general | 1 | 537 | 0.70 (0.21-2.32) | 100 |
| Pertussis | Awareness & Information | policy | 1 | 180 | 4.43 (1.61-12.21) | 100 |
| Pertussis | Disease Susceptibility | contagiousness | 1 | 346 | 1.08 (0.49-2.39) | 100 |
| Pertussis | Vaccine Harm During Pregnancy | general | 1 | 346 | 0.09 (0.01-1.47) | 100 |
| Pertussis | History of previous vaccination | anytime | 1 | 537 | 1.20 (0.61-2.35) | 100 |
| P.Flu | Awareness & Information | policy | 1 | 549 | 3.64 (1.80-7.36) | 100 |
| P.Flu | Disease Severity | death | 1 | 87 | 2.21 (0.58-8.35) | 100 |
| P.Flu | Disease Severity | harmful | 1 | 250 | 5.73 (2.97-11.06) | 100 |
| P.Flu | Disease Susceptibility | contagiousness | 1 | 314 | 0.60 (0.20-1.80) | 100 |
| P.Flu | Vaccine Harm During Pregnancy | preterm | 1 | 544 | 0.26 (0.12-0.58) | 100 |
| P.Flu | Utility of Vaccines | foetus | 1 | 548 | 8.71 (4.17-18.20) | 100 |
| S.Flu | Disease Severity | death | 1 | 85 | 0.08 (0.02-0.26) | 100 |
| S.Flu | Vaccine Harm During Pregnancy | birth | 1 | 84 | 0.10 (0.03-0.36) | 100 |
| S.Flu | Vaccine Harm During Pregnancy | preterm | 1 | 261 | 2.85 (0.66-12.37) | 100 |
| P.Flu = Pandemic Influenza, S.Flu = Seasonal Influenza | | | | | | |

# **Supplementary Material References**

1. Wilson RJ, Paterson P, Jarrett C, Larson HJ. Understanding factors influencing vaccination acceptance during pregnancy globally: A literature review. Vaccine. 2015;33(47):6420-9

2. Abbott D, Morton K. H1N1 vaccine in pregnancy: The response so far. Archives of Disease in Childhood: Fetal and Neonatal Edition. 2010;95(SUPPL. 1)

3. Adeyemi AB, Enabor OO, Ugwu IA, Bello FA, Olayemi OO. Knowledge of hepatitis B virus infection, access to screening and vaccination among pregnant women in Ibadan, Nigeria. 2013. p. 155-9.

4. Ahluwalia IB, Singleton JA, Jamieson DJ, Rasmussen SA, Harrison L. Seasonal influenza vaccine coverage among pregnant women: Pregnancy Risk Assessment Monitoring System. Journal of Women's Health. 2011 2011/05//;20(5):649-51

5. Armitage EP, Camara J, Bah S, et al. Acceptability of intranasal live attenuated influenza vaccine, influenza knowledge and vaccine intent in The Gambia. Vaccine. 2018;36(13):1772-80

6. Arriola CS, Vasconez N, Thompson M, et al. Factors associated with a successful expansion of influenza vaccination among pregnant women in Nicaragua. Vaccine. 2016 2016/02/17/;34(8):1086-90

7. Ashby M, Roussos-Ross D, De Cesare HPJ, Vidrine S, Floy E. Barriers for Tdap vaccination in pregnancy. Obstetrics and Gynecology. 2017;129(Supplement 1):40S

8. Ashelby LC, Trinder J. Swine flu vaccination: Why won't women have it? Archives of Disease in Childhood: Fetal and Neonatal Edition. 2011;96(SUPPL. 1)

9. Azizi FSM, Kew Y, Moy FM. Vaccine hesitancy among parents in a multi-ethnic country, Malaysia. Vaccine. 2017 2017/05/19/;35(22):2955-61

10. Ballas J, Sachs M, Lee W, et al. Predictors and barriers to influenza and Tdap vaccine uptake among women receiving prenatal care within an urban county hospital system. American Journal of Obstetrics & Gynecology. 2015 2015/01//;212(1, S):S289-S90

11. Barrett T, McEntee E, O'Shea A, et al. Influenza vaccination during pregnancy: Prevalence and barriers to uptake? European Journal of General Practice. 2017;23(1):148

12. Beel ER, Rench MA, Montesinos DP, Mayes B, Healy CM. Knowledge and attitudes of postpartum women toward immunization during pregnancy and the peripartum period. Human Vaccines & Immunotherapeutics. 2013 2013/09//;9(9):1926-31

13. Betsch C, Boedeker B, Schmid P, Wichmann O. How baby's first shot determines the development of maternal attitudes towards vaccination. Vaccine. 2018 2018/05/17/;36(21):3018-26

14. Blanchard-Rohner G, Meier S, Ryser J, et al. A study on the acceptability of the influenza vaccine during pregnancy. Swiss Medical Weekly. 2011 2011/08/13/;141(187):34S

15. Bushar JA, Kendrick JS, Ding H, Black CL, Greby SM. Text4baby influenza messaging and influenza vaccination among pregnant women. American Journal of Preventive Medicine. 2017 2017/12//;53(6):845-53

16. Calvo A, Gonzalez R, Pascale JM, et al. Influenza vaccine knowledge and acceptability. American Journal of Tropical Medicine and Hygiene. 2017;97(5 Supplement 1):217

17. Chamberlain AT, Seib K, Ault KA, et al. Impact of a multi-component antenatal vaccine promotion package on improving knowledge, attitudes and beliefs about influenza and Tdap vaccination during pregnancy. Human Vaccines & Immunotherapeutics. 2016 2016;12(8):2017-24

18. Chan OK, Lao TT, Suen SS, Lau TK, Leung TY. Knowledge on hepatitis B infection among pregnant women in a high endemicity area. Patient Education & Counseling. 2011;85(3):516-20

19. Chan O, Suen S, Lao T, Leung K, Yeung S, Leung T. Determinants of hepatitis B vaccine uptake among pregnant Chinese women in Hong Kong. International Journal of Gynecology & Obstetrics. 2009;106(3):232-5

20. Corben P, Leask J. Vaccination hesitancy in the antenatal period: a cross-sectional survey. BMC Public Health. 2018 2018/05/02/;18

21. Danchin MH, Costa-Pinto J, Attwell K, et al. Vaccine decision-making begins in pregnancy: Correlation between vaccine concerns, intentions and maternal vaccination with subsequent childhood vaccine uptake. Vaccine. 2018;36(44):6473-9

22. Dempsey AF, Pyrzanowski J, Donnelly M, et al. Acceptability of a hypothetical group B strep vaccine among pregnant and recently delivered women. Vaccine. 2014 2014/05/01/;32(21):2463-8

23. Deshpande M, Samin K, Banu N, Armstrong M. Factors affecting uptake of the seasonal influenza vaccine in the pregnant women. BJOG: An International Journal of Obstetrics and Gynaecology. 2013 2013/06//;120(1, SI):417

24. Dickin KL, Binchan RK, Purdue SE, Obinya EA. Perceptions of neonatal tetanus and immunization during pregnancy: A report of focus group discussions in Kaduna, Nigeria. International Quarterly of Community Health Education. 1990 01/01/;11(4):371-83

25. Dow-Clarke RA, MacCalder L, Hessel PA. Health behaviours of pregnant women in Fort McMurray, Alberta. Canadian Journal of Public Health. 1994;85(1):33-6

26. Du Y, He H, Zhu X. Investigation and analysis on the willingness for receiving influenza A(H1N1) vaccine among focus groups in Qingyuan city. Journal of Tropical Medicine (Guangzhou). 2010;10(6):738-40

27. Edet EE, Ikpeme BM, Ndifon WO, Oyo-Ita AE. Factors associated with missed opportunities to immunise with tetanus toxoid at a tertiary health institution in Nigeria. The Central African journal of medicine. 1998;44(8):199-202

28. Fell DB, Yasseen AS, Sprague AE, et al. Characteristics of women who received influenza vaccination in a population-based cohort of pregnant women in ontario. American Journal of Epidemiology. 2011;173(SUPPL. 11):S42

29. Ferdinand A. Boosting uptake of flu vaccination in pregnant women. Practice Nurse. 2012;42(15):12-4

30. Ferndale D, Meuter RFI, Watson B, Gallois C. 'You don't know what's going on in there': a discursive analysis of midwifery hospital consultations. Health, Risk & Society. 2017;19(7-8):411-31

31. Ford AJ, Alwan NA. Use of social networking sites and women's decision to receive vaccinations during pregnancy: A cross-sectional study in the UK. Vaccine. 2018 2018/08/23/;36(35):5294-303

32. Frew PM, Owens LE, Saint-Victor DS, Benedict S, Zhang S, Omer SB. Factors associated with maternal influenza immunization decision-making evidence of immunization history and message framing effects. Human Vaccines & Immunotherapeutics. 2014 2014/09//;10(9):2576-83

33. Gazmararian JA, Orenstein W, Prill M, et al. Maternal knowledge and attitudes toward influenza vaccination: A focus group study in metropolitan Atlanta. Clinical Pediatrics. 2010 2010/11//;49(11):1018-25

34. Haile Z, Chertok I, Teweldeberhan A. Determinants of utilization of sufficient tetanus toxoid immunization during pregnancy: Evidence from the Kenya Demographic and Health Survey, 2008-2009. Journal of Community Health. 2013;38(3):492-9

35. Holbeach N, McCarthy E, Howat P. Influenza vaccination during pregnancy 2014: Cohort of new mothers in a Melbourne level II hospital. Journal of Paediatrics and Child Health. 2015;51(SUPPL. 1):122

36. Howland R, Lu E, Diop H. Influenza vaccination among pregnant women - Massachusetts, 2009-2010. Morbidity and Mortality Weekly Report. 2013;62(43):854-7

37. Hu Y, Chen Y, Wang Y, Song Q, Li Q. Prenatal vaccination education intervention improves both the mothers' knowledge and children's vaccination coverage: Evidence from randomized controlled trial from eastern China. Human Vaccines & Immunotherapeutics. 2017 2017;13(6):1477-84

38. Johm P, Jaiteh F, Clarke E, Grietens KP. Understanding maternal and neonatal vaccination acceptance in the Gambia. Tropical Medicine & International Health. 2017 2017/10//;22(1, SI):342-3

39. Jones TF, Ingram LA, Craig AS, Schaffner W. Determinants of influenza vaccination, 2003-2004: Shortages, fallacies and disparities. Clinical Infectious Diseases. 2004 2004/12/15/;39(12):1824-8

40. Kashiba Y. Cross sectional study on factors associating neonatal tetanus immunization among reproductive aged women in Kasur District of Punjab Province, Pakistan; mothers' perceived belief on neonatal tetanus and tetanus toxoid vaccination. Journal of the National Institute of Public Health. 2007;56(4):418-9

41. Kelly E, Power M, Samelson R, Schulkin J. Factors associated with influenza vaccination proportion during pregnancy. Obstetrics and Gynecology. 2016;127(Supplement 1):57S

42. Kennedy ED, Ahluwalia IB, Ding H, Lu PJ, Singleton JA, Bridges CB. Monitoring seasonal influenza vaccination coverage among pregnant women in the United States. American Journal of Obstetrics & Gynecology. 2012;207(3):S9-S16

43. Kim A, Schiller A, Mellon M, Nelson AL, Stohl HE. Tdap vaccine knowledge and acceptance rates during pregnancy in a los angeles public teaching-hospital clinic. Obstetrics and Gynecology. 2018;131(Supplement 1):175S

44. King CL, Chow MY, Leask J, Wiley KE. Australian caregivers' perceptions of influenza vaccination in pregnancy: A mixed methods exploration. Women and Birth. 2018

45. Kowal SP, Jardine CG, Bubela TM. “If they tell me to get it, I'll get it. If they don't ... “: Immunization decision-making processes of immigrant mothers. Canadian Journal of Public Health. 2015 2015/06//MAY;106(4):E230-E5

46. Kumar A, Unnikrishnan B, Rekha T, et al. Awareness and attitude regarding breastfeeding and immunization practices among primigravida attending a tertiary care hospital in southern India. Journal of Clinical and Diagnostic Research. 2015 2015/03//;9(3):LC01-LC5

47. Lo CY, Bedford H, Winyard P, Palit V. Pregnant women's views and experiences of pertussis vaccination during pregnancy: A study in Taiwan. Archives of Disease in Childhood. 2018;103(Supplement 1):A178

48. Lu AB, Halim AA, Dendle C, et al. Influenza vaccination uptake amongst pregnant women and maternal care providers is suboptimal. 2012. p. 4055-9.

49. Lupton D. 'The best thing for the baby': mothers' concepts and experiences related to promoting their infants' health and development. Health, Risk & Society. 2011;13(7/8):637-51

50. Mak DB, Joyce S, Regan AK, et al. Antenatal influenza vaccine increases by 60% between 2012 and 2013. Internal Medicine Journal. 2014 2014/05//;44(3, SI):36-7

51. Massimi A, Rosso A, Marzuillo C, Vacchio MR, De Vito C, Villari P. Vaccine hesitancy: old defeat or new challenge for public health? A survey on pregnant women in Rome. European Journal of Public Health. 2017 2017/11//;27(3)

52. McAuslane H, Andrews N, Coole L, Wensley A. A survey of maternal pertussis vaccine uptake in England. International Journal of Infectious Diseases. 2014 2014/04//;21(1):434

53. McCarthy CM, Arya A. Swine flu vaccination: Uptake and determinants of vaccination in pregnant women and Hospital staff in Ireland. Archives of Disease in Childhood: Fetal and Neonatal Edition. 2010;95(SUPPL. 1)

54. McCarthy EA, Tapper L, Pollock WE, Sommerville M. Influenza vaccination in pregnancy after the 2009 pandemic: Experience in a Melbourne teaching hospital 2010 to 2014. BJOG: An International Journal of Obstetrics and Gynaecology. 2015;122(SUPPL. 2):289

55. McQuaid F, Jones C, Stevens Z, et al. Factors influencing women's attitudes towards antenatal vaccines, group B Streptococcus and clinical trial participation in pregnancy: an online survey. BMJ OPEN. 2016 2016;6(4)

56. McSwiney C, Gibson L. A survey of the uptake of the pertussis (Tdap) vaccination and awareness among mothers of infants (0-24months) in Cork University Hospital (CUH), and among staff of an antenatal clinic in Cork University Maternity Hospital (CUMH). Irish Journal of Medical Science. 2017 2017/06//;186(6):S173-S4

57. Moniz MH, Vitek WS, Akers A, Meyn LA, Beigi RH. Perceptions and acceptance of immunization during pregnancy. Journal of Reproductive Medicine. 2013 2013/10//SEP;58(9-10):383-8

58. Moukarram H, Nargund A, Photiou A, Kiran TSU. Awareness and acceptance of the pandemic influenza (H1N1v 2009) vaccination among antenatal patients in a district general hospital. Journal of Obstetrics and Gynaecology 2012 2012/08//;32(6):537-9

59. Murphy K, McGeer A, De Souza L, Yudin M, Moore S. Knowledge about H1N1 influenza and the vaccine impacts vaccination uptake among pregnant women. American Journal of Obstetrics & Gynecology. 2011 2011/01//;204(1):S255

60. Naidu MA, Krishnaswamy S, Wallace EM, Giles ML. Pregnant women's attitudes toward antenatal pertussis vaccination. Australian & New Zealand Journal of Obstetrics & Gynaecology. 2017 2017/04//;57(2):235

61. Nassar A, Usta I, Awwad J, Ghulmiyyah L, Khoury S, Ghazeeri G. Attitudes of women towards influenza a (H1N1) vaccination during pregnancy. Journal of Maternal-Fetal and Neonatal Medicine. 2010;23(SUPPL. 1):389

62. Nawa N, Kogaki S, Takahashi K, et al. Analysis of public concerns about influenza vaccinations by mining a massive online question dataset in Japan. Vaccine. 2016 2016/06/08/;34(27):3207-13

63. Odumosu MO, Odumosu MO. Mass media and immunization awareness of pregnant women in a Nigerian community. Canadian Journal of Public Health. 1982;73:105-8

64. O'Leary ST, Pyrzanowski J, Brewer SE, et al. Influenza and pertussis vaccination among pregnant women and their infants' close contacts reported practices and attitudes. Pediatric Infectious Disease Journal. 2015 2015/11//;34(11):1244-9

65. Panda B, Stiller R, Panda A. Influenza vaccination during pregnancy and factors for lacking compliance with current CDC guidelines. Journal of Maternal-Fetal and Neonatal Medicine. 2011 2011/03//;24(3):402-6

66. Patwardhan M, Ponce E, Rios Driscoll C, Gonik B. Global diversity in vaccine acceptance during pregnancy. American Journal of Obstetrics and Gynecology. 2015;213(6):894

67. Payakachat N, Hadden KB, Hanner J, Ragland D. Maternal knowledge of pertussis and Tdap vaccine and the use of a vaccine information statement. Health Education Journal. 2018 2018/04//;77(3):322-31

68. Perry J, Towers CV, Weitz B, Wolfe L. Patient reaction to Tdap vaccination in pregnancy. Vaccine. 2017 2017/05/25/;35(23):3064-6

69. Phoxay C, Okumura J, Nakamura Y, Wakai S. Influence of women's knowledge on maternal health care utilization in Southern Laos. Asia-Pacific Journal of Public Health. 2001;13(1):13-9

70. Pollock W, Hay S, McCarthy E, Nolan T, McDonald S. Pandemic h1n1 influenza vaccination uptake in pregnancy. Journal of Paediatrics and Child Health. 2011;47(SUPPL. 1):45-6

71. Ragland D, Hadden K, Payakachat N. Factors associated with maternal knowledge of pertussis and the Tdap vaccine. Journal of Women's Health. 2016 2016/04//;25(4):A28

72. Rahman M. Tetanus toxoid vaccination coverage and differential between urban and rural areas of Bangladesh. East African Journal of Public Health. 2009;6(1):26-31

73. Roosihermiatie B, Nishiyama M, Nakae K. Factors associated with TT (tetanus toxoid) immunization among pregnant women, in Saparua, Maluku, Indonesia. The Southeast Asian journal of tropical medicine and public health. 2000;31(1):91-5

74. Rupali R, Pragati C, Saini NK, Kannan AT. Assessment of immunization coverage among infants and pregnant women in Narela using Lot quality assurance technique. Indian Journal of Public Health Research and Development. 2014;5(1):169-73

75. Sakaguchi S, Weitzner B, Carey N, Bozzo P, Koren G, Einarson A. Pregnant women and receiving the H1N1 vaccine: Perception of risk and determinants of decision-making. Journal of Population Therapeutics and Clinical Pharmacology. 2010;17(1):e227

76. Sasaki TK, Yoshida A, Kotake K. Attitudes about the 2009 H1N1 influenza pandemic among pregnant japanese women and the use of the japanese municipality as a source of information. Southeast Asian Journal of Tropical Medicine and Public Health. 2013;44(3):388-99

77. Sevilla ME. Vaccination in pregnancy: Tetanus diphtheria and acellular pertussis (Tdap) immunization strategy at Hospital General de Ninos Dr Pedro de Elizalde 2013. Tropical Medicine & International Health. 2015;20(SUPPL. 1):278

78. Shafiq Y, Khowaja AR, Yousafzai MT, Ali SA, Zaidi A, Saleem AF. Knowledge, attitudes and practices related to tetanus toxoid vaccination in women of childbearing age: A cross-sectional study in peri-urban settlements of Karachi, Pakistan. Journal of Infection Prevention. 2017;18(5):232-41

79. Steiner B, Swamy GK, Walter EB. Engaging expectant parents to receive Tdap vaccination. American Journal of Perinatology. 2014 2014/05//;31(5):407-12

80. Stringer M, Ratcliff SJ, Gross R. Acceptance of hepatitis B vaccination by pregnant adolescents. MCN: The American Journal of Maternal Child Nursing. 2006;31(1):54-60

81. Thind A. Determinants of tetanus toxoid immunization in pregnancy in rural Bihar. Tropical Doctor. 2005 2005/04//;35(2):75-7

82. Vilca Yengle LM, Campins Marti M, Cabero Roura L, et al. Influenza vaccination in pregnant women. Coverage, practices and knowledge among obstetricians. Medicina Clinica. 2010 2010/02/13/;134(4):146-51

83. Vitek WS, Akers A, Meyn LA, Switzer GE, Lee BY, Beigi RH. Vaccine eligibility and acceptance among ambulatory obstetric and gynecologic patients. Vaccine. 2011 2011/03/03/;29(11):2024-8

84. Wang X, Qin W, Song W, Yue L, Zhu R, Liu H. Influence of Shandong illegal vaccine selling event on vaccination willingness of pregnant women. China Tropical Medicine. 2018;18(1):63-8

85. Wilson RJ, Larson H, Paterson P. Understanding factors influencing vaccination acceptance during pregnancy in Hackney, London. Lancet. 2016 2016/11//;388(2):112

86. Winslade CG, Heffernan CM, Atchison CJ. Experiences and perspectives of mothers of the pertussis vaccination programme in London. Public Health. 2017 2017/05//;146:10-4

87. Wong CY, Thomas NJ, Clarke M, Boros C, Tuckerman J, Marshall HS. Maternal uptake of pertussis cocooning strategy and other pregnancy related recommended immunizations. Human Vaccines & Immunotherapeutics. 2015 2015/05/04/;11(5):1165-72

88. Yeager DP, Toy EC, Baker IB. Influenza vaccination in pregnancy. American Journal of Perinatology. 1999;16(6):283-6

89. Yudin MH, Salaripour M, Sgro MD. Acceptability and Feasibility of Seasonal Influenza Vaccine Administration in an Antenatal Clinic Setting. Journal of Obstetrics and Gynaecology Canada. 2010;32(8):745-8

90. Yudin MH, Salripour M, Sgro MD. Impact of Patient Education on Knowledge of Influenza and Vaccine Recommendations Among Pregnant Women. Journal of Obstetrics and Gynaecology Canada. 2010;32(3):232-7

91. Moher D, Liberati A, Tetzlaff J, Altman D, Group TP. Preferred Reporting Items for Systematic Reviews and Meta-Analyses: The PRISMA Statement. . PLoS Med. 2009;6(7)

92. Aromataris E, Munn Z, (Editors). Joanna Briggs Institute Reviewer's Manual. 2017.

93. Bodeker B, Betsch C, Wichmann O. Skewed risk perceptions in pregnant women: the case of influenza vaccination. BMC Public Health 2015 2015/12/29/;15

94. Henninger M, Naleway A, Crane B, Donahue J, Irving S. Predictors of seasonal influenza vaccination during pregnancy. Obstetrics & Gynecology. 2013 2013/04//;121(4):741-9

95. Henninger ML, Irving SA, Thompson M, et al. Factors associated with seasonal influenza vaccination in pregnant women. Journal of Women's Health (15409996). 2015;24(5):394-402

96. Ugezu C, Essajee M. Exploring patients' awareness and healthcare professionals' knowledge and attitude to pertussis and influenza vaccination during the antenatal periods in Cavan Monaghan general hospital. Human Vaccines & Immunotherapeutics. 2018 2018;14(4):978-83

97. Abasi E, Tahmasebi H, Tofighi M, Zafari M. Knowledge and practice about influenza vaccination and compliance with influenza immunization among pregnant women in Sari, 2013. Journal of Nursing & Midwifery Sciences. 2015;2(3):25-31

98. Agricola E, Gesualdo F, Alimenti L, et al. Knowledge attitude and practice toward pertussis vaccination during pregnancy among pregnant and postpartum Italian women. Human Vaccines & Immunotherapeutics. 2016 2016;12(8):1982-8

99. Arriola CS, Vasconez N, Bresee J, Ropero AM. Knowledge, attitudes and practices about influenza vaccination among pregnant women and healthcare providers serving pregnant women in Managua, Nicaragua. Vaccine. 2018;36(25):3686-93

100. Ashfaq U, Ambreen S, Ashraf A. Maternal and neonatal tetanus: A case study of Pakistan. International Journal of Advanced Biotechnology and Research. 2017 2017;8(4):259-69

101. Barrett T, McEntee E, Drew R, et al. Influenza vaccination in pregnancy: Vaccine uptake, maternal and healthcare providers' knowledge and attitudes. A quantitative study. BJGP Open. 2018;2(3)

102. Beigi RH, Switzer GE, Meyn LA. Acceptance of a pandemic avian influenza vaccine in pregnancy. Journal of Reproductive Medicine. 2009 2009/06//;54(6):341-6

103. Ben Natan M, El Kravchenko B, Sakashidlo K, Mor S. What drives pregnant women's decisions to accept the pertussis vaccine? Applied Nursing Research. 2017 2017/12//;38:60-3

104. Bettinger JA, Greyson D, Money D. Attitudes and beliefs of pregnant women and new mothers regarding influenza vaccination in British Columbia. Journal of Obstetrics and Gynaecology Canada. 2016 2016/11//;38(11):1045-52

105. Bhaskar E, Thobias S, Anthony S, Kumar V, Navaneethan. Vaccination rates for pandemic influenza among pregnant women: An early observation from Chennai, South India. Lung India. 2012 Jul-Sep;29(3):232-5

106. Blanchard-Rohner G, Meier S, Ryser J, et al. Acceptability of maternal immunization against influenza: the critical role of obstetricians. Journal of Maternal-Fetal & Neonatal Medicine. 2012;25(9):1800-9

107. Blondel B, Mahjoub N, Drewniak N, Launay O, Goffinet F. Failure of the vaccination campaign against A(H1N1) influenza in pregnant women in France: Results from a national survey. Vaccine. 2012 2012/08/17/;30(38):5661-5

108. Bodeker B, Walter D, Reiter S, Wichmann O. Cross-sectional study on factors associated with influenza vaccine uptake and pertussis vaccination status among pregnant women in Germany. Vaccine. 2014;32(33):4131-9

109. Campbell H, Van Hoek AJ, Bedford H, et al. Attitudes to immunisation in pregnancy among women in the UK targeted by such programmes. British Journal of Midwifery. 2015;23(8):566-73

110. Castro-Sanchez E, Vila-Candel R, Soriano-Vidal FJ, Navarro-Illana E, Diez-Domingo J. Influence of health literacy on acceptance of influenza and pertussis vaccinations: a cross-sectional study among Spanish pregnant women. BMJ Open. 2018 2018/09//;8(7)

111. Celikel A, Ustunsoz A, Guvenc G. Determination of vaccination status of pregnant women during pregnancy and the affecting factors. Journal of Clinical Nursing. 2014 2014/08//;23(15-16):2142-50

112. Chamberlain AT, Seib K, Ault KA, et al. Factors associated with intention to receive influenza and tetanus, diphtheria, and acellular pertussis (Tdap) vaccines during pregnancy: A focus on vaccine hesitancy and perceptions of disease severity and vaccine safety. PLoS Currents. 2015;7(OUTBREAKS)

113. Chamberlain AT, Berkelman RL, Ault KA, Rosenberg ES, Orenstein WA, Omer SB. Trends in reasons for non-receipt of influenza vaccination during pregnancy in Georgia, 2004-2011. Vaccine. 2016 2016/03/18/;34(13):1597-603

114. D'Alessandro A, Napolitano F, D'Ambrosio A, Angelillo IF. Vaccination knowledge and acceptability among pregnant women in Italy. Human Vaccines & Immunotherapeutics. 2018;14(7):1573-9

115. Dempsey AF, Brewer SE, Sevick C, Pyrzanowski J, Mazzoni S, O'Leary ST. Tdap vaccine attitudes and utilization among pregnant women from a high-risk population. Human Vaccines & Immunotherapeutics. 2016 2016;12(4):872-8

116. Ding H, Santibanez TA, Jamieson DJ, et al. Influenza vaccination coverage among pregnant women--National 2009 H1N1 Flu Survey (NHFS). American Journal of Obstetrics and Gynecology. 2011;204(6 Suppl 1):S96-106

117. Ditsungnoen D, Greenbaum A, Praphasiri P, et al. Knowledge, attitudes and beliefs related to seasonal influenza vaccine among pregnant women in Thailand. Vaccine. 2016 2016/04/19/;34(18):2141-6

118. Dlugacz Y, Fleischer A, Carney MT, et al. 2009 H1N1 vaccination by pregnant women during the 2009-10 H1N1 influenza pandemic. Obstetrical & Gynecological Survey. 2012 2012/08//;67(8):466-7

119. Donaldson B, Jain P, Holder BS, Lindsey B, Regan L, Kampmann B. What determines uptake of pertussis vaccine in pregnancy? A cross sectional survey in an ethnically diverse population of pregnant women in London. Vaccine. 2015;33(43):5822-8

120. Drees M, Johnson O, Wong E, et al. Acceptance of 2009 H1N1 influenza vaccine among pregnant women in Delaware. American Journal of Perinatology. 2012 2012/04//;29(4):289-94

121. Drees M, Tambourelli B, Denstman A, et al. Sustained high influenza vaccination rates and decreased safety concerns among pregnant women during the 2010-2011 influenza season. Vaccine. 2013 2013/01/02/;31(2):362-6

122. Edmonds BMT, Coleman J, Armstrong K, Shea JA. Risk perceptions, worry, or distrust: What drives pregnant women's decisions to accept the H1N1 vaccine? Maternal & Child Health Journal. 2011 2011/11//;15(8):1203-9

123. Eppes C, Wu A, You W, Cameron KA, Garcia P, Grobman W. Barriers to influenza vaccination among pregnant women. Vaccine. 2013 2013/06/12/;31(27):2874-8

124. Fabry P, Gagneur A, Pasquier JC. Determinants of A (H1N1) vaccination: Cross-sectional study in a population of pregnant women in Quebec. Vaccine. 2011;29(9):1824-9

125. Fisher BM, Scott J, Hart J, Winn VD, Gibbs RS, Lynch AM. Behaviors and perceptions regarding seasonal and H1N1 influenza vaccination during pregnancy. American Journal of Obstetrics and Gynecology. 2011 2011/06//;204(6, 1):S107-S11

126. Fleming JA, Baltrons R, Rowley E, et al. Implementation of maternal influenza immunization in El Salvador: Experiences and lessons learned from a mixed-methods study. Vaccine. 2018;36(28):4054-61

127. Fridman D, Steinberg E, Azhar E, Weedon J, Wilson TE, Minkoff H. Predictors of H1N1 vaccination in pregnancy. American Journal of Obstetrics and Gynecology. 2011 2011/06//;204(6, 1):S124-S7

128. Gaudelus J, Martinot A, Denis F, et al. Vaccination of pregnant women in France. Medecine et Maladies Infectieuses. 2016;46(8):424-8

129. Goldfarb I, Panda B, Wylie B, Riley L. Uptake of influenza vaccine in pregnant women during the 2009 H1N1 influenza pandemic. American Journal of Obstetrics and Gynecology. 2011 2011/06//;204(6, 1):S112-S5

130. Gorman JR, Brewer NT, Wang JB, Chambers CD. Theory-based predictors of influenza vaccination among pregnant women. Vaccine 2012 2012/12/17/;31(1):213-8

131. Gul R, Bibi S, Khan HM, Ayub R, Alam SR, Afridi A. Frequency of tetanus toxiod vaccination in pregnant women attending a tertiary care hospital. Journal of Medical Sciences (Peshawar). 2016;24(4):220-3

132. Hallissey R, O'Connell A, Warren M. Factors that influence uptake of vaccination in pregnancy. Irish Medical Journal. 2018;111(3):1-4

133. Halperin BA, MacKinnon-Cameron D, McNeil S, Kalil J, Halperin SA. Maintaining the momentum: Key factors influencing acceptance of influenza vaccination among pregnant women following the H1N1 pandemic. Human Vaccines & Immunotherapeutics. 2014 2014/12/02/;10(12):3629-41

134. Hasnain S, Sheikh NH. Causes of low tetanus toxoid vaccination coverage in pregnant women in Lahore district, Pakistan. Eastern Mediterranean Health Journal. 2007;13(5):1142-52

135. Hassan AM, Shoman AE, Abo-Elezz NF, Amer MM. Tetanus vaccination status and its associated factors among women attending a primary healthcare center in Cairo governorate, Egypt. Journal of the Egyptian Public Health Association. 2016 2016/09//;91(3):127-34

136. Hayles EH, Cooper SC, Wood N, Skinner SR, Sinn JHK. Pertussis Booster Vaccination in Pregnancy: Women Who had it Compared to Those Who Waited. Procedia in Vaccinology. 2015;9:59-65

137. Healy CM, Rench MA, Montesinos DP, Ng N, Swaim LS. Knowledge and attitiudes of pregnant women and their providers towards recommendations for immunization during pregnancy. Vaccine 2015 2015/10/05/;33(41):5445-51

138. Hill L, Burrell B, Walls T. Factors influencing women's decisions about having the pertussis-containing vaccine during pregnancy. Journal of Primary Health Care 2018 2018/03//;10(1):62-7

139. Honarvar B, Odoomi N, Mahmoodi M, et al. Acceptance and rejection of influenza vaccination by pregnant women in southern Iran: physicians' role and barriers. Human Vaccines & Immunotherapeutics. 2012;8(12):1860-6

140. Hu Y, Wang Y, Liang H, Chen Y. Seasonal influenza vaccine acceptance among pregnant women in Zhejiang Province, China: Evidence based on health belief model. International Journal of Envrionmental Research and Public Health. 2017 2017/12//;14(12)

141. Jadoon A, Rehman MU, Hanif A, Ashar SM, Yasmeen S. Assessment of the vaccination status of pregnant mothers and their infants after birth with tetanus toxoid in District Rawalpindi of Pakistan. Advances in Human Biology. 2016 2016/04//JAN;6(1):12-5

142. Kang HS, De Gagne JC, Kim JH. Attitudes, intentions, and barriers toward influenza vaccination among pregnant Korean women. Health Care for Women International. 2015;36(9):1026-38

143. Kay MK, Koelemay KG, Kwan-Gett TS, Cadwell BL, Duchin JS. 2009 pandemic influenza a vaccination of pregnant women--King County, Washington State, 2009-2010. American Journal of Public Health. 2012;102 Suppl 3:S368-74

144. Kfouri RdA, Richtmann R. Influenza vaccine in pregnant women: immunization coverage and associated factors. Einstein (Sao Paulo, Brazil). 2013;11(1):53-7

145. Khan AA, Varan AK, Esteves-Jaramillo A, et al. Influenza vaccine acceptance among pregnant women in urban slum areas, Karachi, Pakistan. Vaccine 2015 2015/09/22/;33(39):5103-9

146. Kouassi DP, Coulibaly D, Foster L, et al. Vulnerable groups within a vulnerable population: awareness of the A(H1N1)pdm09 pandemic and willingness to be vaccinated among pregnant women in Ivory Coast. Journal of Infectious Diseases. 2012 01/01/;206 Suppl 1:S114-S20

147. Koul PA, Bali NK, Saima A, et al. Poor uptake of influenza vaccination in pregnancy in northern India. International Journal of Gynecology & Obstetrics. 2014;127(3):234-7

148. Krishnaswamy S, Cheng AC, Wallace EM, Buttery J, Giles ML. Understanding the barriers to uptake of antenatal vaccination by women from culturally and linguistically diverse backgrounds: A cross-sectional study. Human Vaccines & Immunotherapeutics. 2018;14(7):1591-8

149. Kriss JL, Albert AP, Carter VM, et al. Disparities in Tdap vaccination and vaccine information needs among pregnant women in the United States. Maternal & Child Health Journal. 2018:No-Specified

150. Lau JTF, Cai Y, Tsui HY, Choi KC. Prevalence of influenza vaccination and associated factors among pregnant women in Hong Kong. Vaccine 2010 2010/07/26/;28(33):5389-97

151. Lotter K, Regan AK, Thomas T, Effler PV, Mak DB. Antenatal influenza and pertussis vaccine uptake among Aboriginal mothers in Western Australia. Australian & New Zealand Journal of Obstetrics & Gynaecology. 2018 2018/08//;58(4):417-24

152. Loubet P, Guerrisi C, Turbelin C, et al. Influenza during pregnancy: Incidence, vaccination coverage and attitudes toward vaccination in the French web-based cohort G-GrippeNet. Vaccine 2016 2016/04/29/;34(20):2390-6

153. MacDougall DM, Halperin BA, Langley JM, et al. Knowledge, attitudes, beliefs, and behaviors of pregnant women approached to participate in a Tdap maternal immunization randomized, controlled trial. Human Vaccines & Immunotherapeutics. 2016 2016;12(4):879-85

154. Maher L, Hope K, Torvaldsen S, et al. Influenza vaccination during pregnancy: Coverage rates and influencing factors in two urban districts in Sydney. Vaccine 2013 2013/11/12/;31(47):5557-64

155. Mak DB, Regan AK, Joyce S, Gibbs R, Effler PV. Antenatal care provider's advice is the key determinant of influenza vaccination uptake in pregnant women. Australian & New Zealand Journal of Obstetrics & Gynaecology. 2015 2015/04//;55(2):131-7

156. Mak DB, Regan AK, Vo DT, Effler PV. Antenatal influenza and pertussis vaccination in Western Australia: a cross-sectional survey of vaccine uptake and influencing factors. BMC Pregnancy and Childbirth. 2018 2018/10/24/;18

157. de Mattos LMBBd, Caiaffa WT, Bastos RR, Tonelli E. Missed opportunities for tetanus immunization of pregnant women in Juiz de Fora, Minas Gerais, Brazil. Rev Panam Salud Publica. 2003;14(5):350-4

158. Maurici M, Dugo V, Zaratti L, et al. Knowledge and attitude of pregnant women toward flu vaccination: a cross-sectional survey. Journal of Maternal-Fetal & Neonatal Medicine. 2016 2016;29(19):3147-50

159. Mayet AY, Al-Shaikh GK, Al-Mandeel HM, Alsaleh NA, Hamad AF. Knowledge, attitudes, beliefs, and barriers associated with the uptake of influenza vaccine among pregnant women. Saudi Pharmaceutical Journal 2017 2017/01//;25(1):76-82

160. McCarthy EA, Pollock WE, Nolan T, Hay S, McDonald S. Improving influenza vaccination coverage in pregnancy in Melbourne 2010-2011. Australian & New Zealand Journal of Obstetrics & Gynaecology. 2012 2012/08//;52(4):334-41

161. McCarthy EA, Pollock WE, Tapper L, Sommerville M, McDonald S. Increasing uptake of influenza vaccine by pregnant women post H1N1 pandemic: a longitudinal study in Melbourne, Australia, 2010 to 2014. BMC Pregnancy and Childbirth. 2015 2015/03/05/;15

162. McQuaid F, Stevens Z, Meddaugh G, et al. Antenatal vaccination against Group B streptococcus: attitudes of pregnant women and healthcare professionals in the UK towards participation in clinical trials and routine implementation. Acta Obstetricia et Gynecologica Scandinavica. 2018;97(3):330-40

163. Mitra J, Manna A. An assessment of missed opportunities for immunization in children and pregnant women attending different health facilities of a state hospital. Indian Journal of Public Health. 1997;41(1):31-2

164. Mohammed H, Clarke M, Koehler A, Watson M, Marshall H. Factors associated with uptake of influenza and pertussis vaccines among pregnant women in South Australia. PLOS ONE. 2018 2018/06/14/;13(6)

165. Napolitano F, Napolitano P, Angelillo IF. Seasonal influenza vaccination in pregnant women: knowledge, attitudes, and behaviors in Italy. BMC Infectious Diseases 2017 2017/01/09/;17

166. O'Grady K-AF, Dunbar M, Medlin LG, et al. Uptake of influenza vaccination in pregnancy amongst Australian Aboriginal and Torres Strait Islander women: a mixed-methods pilot study. BMC Research Notes. 2015;8:169

167. Og Son K, Sung Won Y. Current state of influenza vaccination and factors affecting vaccination rate among pregnant women. Journal of Korean Academy of Nursing. 2014;44(5):534-41

168. Ozer A, Arikan DC, Kirecci E, Ekerbicer HC. Status of pandemic influenza vaccination and factors affecting it in Turkish pregnant women. Journal of Maternal-Fetal and Neonatal Medicine. 2010;23(SUPPL. 1):315

169. Ozkaya Parlakay A, Kara O, Kara A, Ozyuncu O. The perspective of pregnant women on pandemic influenza vaccine before pandemics. Turkiye Klinikleri Journal of Medical Sciences. 2012;32(6):1618-22

170. Puchalski S. Current attitudes and practices among pregnant women toward influenza immunization. Journal of Pregnancy and Child Health. 2015;2:4:2376-127X

171. Regan AK, Mak DB, Hauck YL, Gibbs R, Tracey L, Effler PV. Trends in seasonal influenza vaccine uptake during pregnancy in Western Australia: Implications for midwives. Women and Birth. 2016 2016/10//;29(5):423-9

172. Sakaguchi S, Weitzner B, Carey N, et al. Pregnant women's perception of risk with use of the H1N1 vaccine. Journal of Obstetrics and Gynaecology Canada. 2011;33(5):460-7

173. Siddiqui M, Khan AA, Varan AK, et al. Intention to accept pertussis vaccine among pregnant women in Karachi, Pakistan. Vaccine 2017 2017/09/25/;35(40):5352-9

174. Silverman NS, Greif A. Influenza vaccination during pregnancy - Patients' and physicians' attitudes. Journal of Reproductive Medicine. 2001 2001/11//;46(11):989-94

175. Song Y, Zhang T, Chen L, et al. Increasing seasonal influenza vaccination among high risk groups in China: Do community healthcare workers have a role to play? Vaccine 2017 2017/07/24/;35(33):4060-3

176. Stark LM, Power ML, Turrentine M, et al. Influenza vaccination among pregnant women: Patient beliefs and medical provider practices. Infectious Diseases in Obstetrics & Gynecology. 2016:1-8

177. SteelFisher GK, Blendon RJ, Bekheit MM, et al. Novel pandemic A (H1N1) influenza vaccination among pregnant women: motivators and barriers. American Journal of Obstetrics and Gynecology. 2011 2011/06//;204(6, 1):S116-S23

178. Strassberg ER, Power M, Schulkin J, et al. Patient attitudes toward influenza and tetanus, diphtheria and acellular pertussis vaccination in pregnancy. Vaccine 2018 2018/07/16/;36(30):4548-54

179. Taksdal SE, Mak DB, Joyce S, et al. Predictors of uptake of influenza vaccination--a survey of pregnant women in Western Australia. Australian Family Physician. 2013;42(8):582-6

180. Tarrant M, Wu KM, Yuen CYS, Cheung KL, Chan VHS. Determinants of 2009 A/H1N1 influenza vaccination among pregnant women in Hong Kong. Maternal & Child Health Journal. 2013 2013/01//;17(1):23-32

181. Tong A, Biringer A, Ofner-Agostini M, Upshur R, McGeer A. A cross-sectional study of maternity care providers' and women's knowledge, attitudes, and behaviours towards influenza vaccination during pregnancy. Journal of Obstetrics and Gynaecology Canada. 2008;30(5):404-10

182. Tuells J, Rodriguez-Blanco N, Torrijos JLD, Vila-Candel R, Bonmati AN. Vaccination of pregnant women in the Valencian community during the 2014-15 influenza season: A multicentre study. Revista Espanola de Quimioterapia. 2018;31(4):344-52

183. van Lier A, Steens A, Ferreira JA, van der Maas NAT, de Melker HE. Acceptance of vaccination during pregnancy: Experience with 2009 influenza A (H1N1) in the Netherlands. Vaccine. 2012

184. Varan AK, Esteves-Jaramillo A, Richardson V, Esparza-Aguilar M, Cervantes-Powell P, Omer SB. Intention to accept Bordetella pertussis booster vaccine during pregnancy in Mexico City. Vaccine 2014 2014/02/07/;32(7):785-92

185. Vila-Candel R, Navarro-Illana P, Navarro-Illana E, et al. Determinants of seasonal influenza vaccination in pregnant women in Valencia, Spain. BMC Public Health. 2016;16(1):1173-

186. White SW, Petersen RW, Quinlivan JA. Pandemic (H1N1) 2009 influenza vaccine uptake in pregnant women entering the 2010 influenza season in Western Australia. Medical Journal of Australia. 2010 2010/10/04/;193(7):405-7

187. Wilcox CR, Bottrell K, Paterson P, et al. Influenza and pertussis vaccination in pregnancy: Portrayal in online media articles and perceptions of pregnant women and healthcare professionals. Vaccine. 2018

188. Wilcox CR, Calvert A, Metz J, et al. Determinants of Influenza and Pertussis Vaccination Uptake in Pregnancy: A Multicenter Questionnaire Study of Pregnant Women and Healthcare Professionals. The Pediatric infectious disease journal. 2019 Jun;38(6):625-30

189. Wiley KE, Massey PD, Cooper SC, et al. Uptake of influenza vaccine by pregnant women: a cross-sectional survey. Medical Journal of Australia. 2013;198(7):373-5

190. Wiley KE, Massey PD, Cooper SC, Wood N, Quinn HE, Leask J. Pregnant women's intention to take up a post-partum pertussis vaccine, and their willingness to take up the vaccine while pregnant: A cross sectional survey. Vaccine 2013 2013/08/20/;31(37):3972-8

191. Ymba A, Perrey C. Acceptability of tetanus toxoid vaccine by pregnant women in two health centres in Abidjan (Ivory Coast). 2003. p. 3497-500.

192. Yudin MH, Salaripour M, Sgro MD. Pregnant women's knowledge of influenza and the use and safety of the influenza vaccine during pregnancy. Journal of Obstetrics and Gynaecology Canada. 2009;31(2):120-5

193. Yuen CYS, Fong DYT, Lee ILY, Chu S, Siu ES-m, Tarrant M. Prevalence and predictors of maternal seasonal influenza vaccination in Hong Kong. Vaccine 2013 2013/10/25/;31(45):5281-8

194. Yun X, Xu J. Survey of attitude toward immunization of H1N1 influenza A vaccine of pregnant women in Guangzhou. Journal of Tropical Medicine (Guangzhou). 2010;10(9):1136-40

195. Barroso Pereira BF, Santos Martins MA, de Andrade Barbosa TL, Oliveira e Silva CS, Xavier Gomes LM. Reasons why pregnant women did not vaccinate against Influenza A H1N1. Ciencia & Saude Coletiva. 2013 2013/06//;18(6):1745-52

196. Cassady D, Castaneda X, Ruelas MR, Vostrejs MM, Andrews T, Osorio L. Pandemics and vaccines: Perceptions, reactions, and lessons learned from hard-to-reach latinos and the H1N1 campaign. Journal of Health Care for the Poor and Underserved. 2012 2012/08//;23(3):1106-22

197. Collins J, Alona I, Tooher R, Marshall H. Increased awareness and health care provider endorsement is required to encourage pregnant women to be vaccinated. Human Vaccines & Immunotherapeutics. 2014 2014/10//;10(10):2922-9

198. Gauld NJ, Braganza CS, Babalola OO, Huynh TT, Hook SM. Reasons for use and non-use of the pertussis vaccine during pregnancy: an interview study. Journal of Primary Health Care 2016 2016/12//;8(4):344-50

199. Kharbanda EO, Vargas CY, Castano PM, Lara M, Andres R, Stockwell MS. Exploring pregnant women's views on influenza vaccination and educational text messages. Preventive Medicine. 2011 2011/01//;52(1):75-7

200. Larson Williams A, McCloskey L, Mwale M, et al. "When you are injected, the baby is protected:" Assessing the acceptability of a maternal Tdap vaccine based on mothers' knowledge, attitudes, and beliefs of pertussis and vaccinations in Lusaka, Zambia. Vaccine. 2018;36(21):3048-53

201. Lohiniva A-L, Barakat A, Dueger E, Restrepo S, El Aouad R. A qualitative study of vaccine acceptability and decision making among pregnant women in Morocco during the A (H1N1) pdm09 pandemic. PLOS ONE. 2014;9:96244-

202. Lohm D, Flowers P, Stephenson N, Waller E, Davis MDM. Biography, pandemic time and risk: Pregnant women reflecting on their experiences of the 2009 influenza pandemic. Health (London, England : 1997). 2014;18(5):493-508

203. Lynch MM, Mitchell EW, Williams JL, et al. Pregnant and recently pregnant women's perceptions about Influenza A pandemic (H1N1) 2009: Implications for public health and provider communication. Maternal & Child Health Journal. 2012 2012/11//;16(8):1657-64

204. Maisa A, Milligan S, Quinn A, et al. Vaccination against pertussis and influenza in pregnancy: A qualitative study of barriers and facilitators. Public Health. 2018 2018/09//;162:111-7

205. Marsh H, Malik F, Shapiro E, Omer S, Frew P. Message framing strategies to increase influenza immunization uptake among pregnant African American women. Maternal & Child Health Journal. 2014;18(7):1639-47

206. McQuaid F, Pask S, Locock L, et al. Attitudes towards antenatal vaccination, Group B streptococcus and participation in clinical trials: Insights from focus groups and interviews of parents and healthcare professionals. Vaccine 2016 2016/07/25/;34(34):4056-61

207. Meharry P, Colson E, Grizas A, Stiller R, Vázquez M. Reasons why women accept or reject the trivalent inactivated influenza vaccine (TIV) during pregnancy. Maternal & Child Health Journal. 2013;17(1):156-64

208. O'Shea A, Cleary B, McEntee E, et al. To vaccinate or not to vaccinate? Women's perception of vaccination in pregnancy: A qualitative study. BJGP Open. 2018;2(2)

209. Richun L, Ruiqian X, Chong Y, Rainey J, Ying S, Greene C. Identifying ways to increase seasonal influenza vaccine uptake among pregnant women in China: A qualitative investigation of pregnant women and their obstetricians. Vaccine 2018 2018/05/31/;36(23):3315-22

210. Schindler M, Blanchard-Rohner G, Meier S, de Tejada BM, Siegrist CA, Burton-Jeangros C. Vaccination against seasonal flu in Switzerland: The indecision of pregnant women encouraged by healthcare professionals. Revue D Epidemiologie et de Sante Publique. 2012 2012/12//;60(6):447-53

211. Wiley KE, Cooper SC, Wood N, Leask J. Understanding pregnant women's attitudes and behavior toward influenza and pertussis vaccination. Qualitative Health Research. 2015 2015/03//;25(3):360-70

212. Yuen CYS, Dodgson JE, Tarrant M. Perceptions of Hong Kong Chinese women toward influenza vaccination during pregnancy. Vaccine 2016 2016/01/02/;34(1):33-40
