# Supplementary material for: Factors that influence vaccination decision-making among pregnant women: A systematic review and meta-analysis
Source: PLoS One. 2020 Jul 9;15(7):e0234827. doi: 10.1371/journal.pone.0234827 (PMC7347125; doi:10.1371/journal.pone.0234827)
Supplement: Attachment — Submitted filename: Response to Reviewers.doc [file pone.0234827.s005.doc]

**Responses to Reviewer Comments**

**Reviewer 1 Comment 1:** The authors have done a very nice job of addressing all the submitted reviewer comments and provided appropriate and responsive modifications to the manuscript. The additional details and content that have been added are very helpful in providing important context to this review. The one comment I will make is in reference to the authors comment that there are no stated maternal vaccination targets available. While this is specific to the US, there is the Healthy People 2020 target of achieving influenza vaccination coverage of 80% among pregnant women. This is not presented as an ultimate target, but does suggest that there is a target goal. If the authors think this is useful, it might be worth using to help substantiate the idea that 70% coverage is considered "low".

**Response:** Thank you, this is a very helpful suggestion. We have added the statement on Line 77-78 and adapted our references:

*“The United States Healthy People 2020 campaign sets a target to achieve influenza vaccination coverage of 80% among pregnant women [4].”*

**Reviewer 3 Comment 2:**  Nicely written and important work, and concerns of the reviewers were sufficiently addressed. Only minor comments: a) Please provide references in line 106. b) Motivational interviewing techniques are strategies that can increase vaccine acceptance. Please include this strategy in your perspectives in the discussion.

**Response**:

a) References have now been provided for line 106. References [11-15] are inserted.

b) To include interventions such as motivational interviewing we have adapted Line 554-556 and adapted our references:

*“Interventions recommended to improve maternal vaccination uptake have ranged from text reminders for prospective mothers to educational videos and motivational interviewing techniques for HCPs [110-112].”*

[110] Gagneur A. Motivational interviewing: A powerful tool to address vaccine hesitancy. Can Commun Dis Rep. 2020, 46(4): 93-97. doi:10.14745/ccdr.v46i04a06.
